# Decadentate Acyclic Chelators for Lanthanum Radiopharmaceuticals

**DOI:** 10.1021/acs.jmedchem.5c01558

**Published:** 2025-08-19

**Authors:** Antía Freire-García, Yasniel Babi Araujo, Melinda Wuest, Balázs Szilágyi, Enikő Madarasi, Laura Valencia, Saray Argibay-Otero, Aurora Rodríguez-Rodríguez, David Esteban-Gómez, Gyula Tircsó, Frank Wuest, Carlos Platas-Iglesias

**Affiliations:** † CICA - Centro Interdisciplinar de Química e Bioloxía and Departamento de Química, Facultade de Ciencias, 16737 Universidade da Coruña, 15071 A Coruña, Galicia, Spain; ‡ Department of Oncology, 3158 University of Alberta, Edmonton, Alberta T6G 1Z2, Canada; § Department of Physical Chemistry, Faculty of Science and Technology, University of Debrecen, H-4032 Debrecen, Hungary; ∥ Doctoral School of Chemistry, University of Debrecen, H-4032 Debrecen, Hungary; ⊥ Departamento de Química Inorgánica, Facultad de Ciencias, 16784 Universidade de Vigo, As Lagoas, Marcosende, 36310 Pontevedra, Spain

## Abstract

Two decadentate acyclic chelators bearing four picolinic acid groups appended on either an ethylenediamine (H_4_TPAEN) or a *trans*-1,2-cyclohexyldiamine (H_4_TPADAC) unit were explored as candidates for lanthanum-based radiopharmaceutical development. The two chelators form ten-coordinated complexes with La^3+^ in the solid state, as evidenced by the corresponding X-ray structures and solution NMR studies. The La^3+^ complexes of TPAEN^4–^ and TPADAC^4–^ are characterized by high thermodynamic stability constants of log *K*_LaL_ = 19.16(8) and 19.55(1), respectively. Kinetics studies indicate that the complexes dissociate following the acid-catalyzed and Cu^2+^-assisted pathways. Quantitative radiolabeling of both chelators with [^135^La]­La^3+^ was achieved at pH ∼ 4–5 using straightforward protocols and low concentrations of the chelator (3 μM). Both *in vitro* and *in vivo* studies indicate that the [^135^La]­La^3+^ complex of TPAEN^4–^ is significantly more stable than the TPADAC^4–^ analogue, with the former remaining intact and stable even after 60 min *in vivo* when injected to healthy mice.

## Graphical abstract

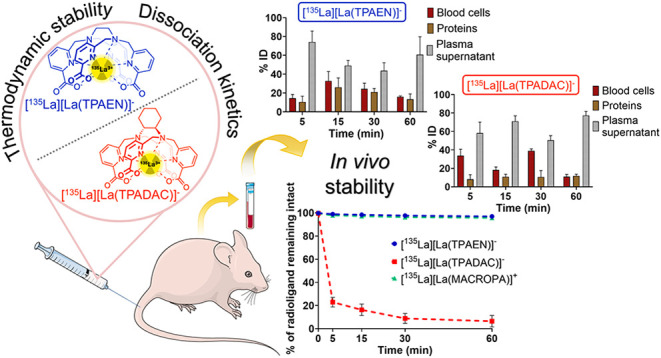

## Introduction

Lanthanum radioisotopes are promising candidates for the development of radiopharmaceuticals thanks to their interesting decay properties and the possibility to combine therapeutic and diagnostic approaches.^1,2^ Indeed, both ^132^La and ^133^La are positron emitters with great potential in positron emission tomography (PET) due to the availability of production routes in medical cyclotrons^3–7^ and their moderate half-lives of 4.6 and 3.9 h, respectively.^8^ Another short-lived positron-emitter lanthanum radioisotope, ^134^La (*t*_1/2_ = 6.5 min), can be obtained from ^134^Ce (*t*_1/2_ = 3.2 d),^9^ paving the way for the use of the ^134^Ce/^134^La pair as an *in vivo* generator.^10^ These positron emitting radioisotopes have been proposed as diagnostic pairs for the ^225^Ac and ^135^La radiotherapeutics.^2,5,10–15^ The latter is an Auger-Meitner emitter with *t*_1/2_ = 19.5 h suitable for therapeutic applications.^16^

The coordination chemistry in aqueous media of La^3+^ and the remaining lanthanide ions (Ln^3+^) is conditioned by their hard acid character according to Pearson’s classification^17,18^ and their tendency to reach high coordination numbers, often 8–9.^19,20^ Stable complexation of the Ln^3+^ is often achieved with derivatives of macrocyclic chelator DOTA, which has dominated the medical applications based on these metal ions.^21,22^ However, DOTA derivatives often experience slow complexation kinetics, which pose some limitations for radiopharmaceutical development.^23,24^ Furthermore, La^3+^ is the ion of the lanthanide series with the largest ionic radius,^25^ which in some cases results in the formation of ten- or even 11-coordinate complexes.^26–29^ As a result, hexadentate chelators such as H_3_NOTA and DiAmSar are not well suited for lanthanide complexation. Enlarging the cavity of H_4_DOTA to give H_4_TETA was also found to be detrimental in terms of the stability of lanthanide complexes.^30^ In addition, the Ac^3+^ ion is the largest among the trivalent metal ions of the periodic table (excluding the transactinide elements). Thus, the development of theranostic pairs based on La and Ac radioisotopes requires designing new chelators with adequate properties. So far, macrocyclic chelators such as H_2_MACROPA, H_4_PYTA and H_4_DO3Apic have been established as promising chelators for these radioisotopes, with H_2_MACROPA being considered the leading chelator for Ac^3+^ complexation (Chart 1).^1,27,31^ Nevertheless, the nature of the chelating unit may have a large impact in the performance of bifunctional chelators, and thus expanding the range of chelators for the complexation of La^3+^ is highly important for radiopharmaceutical development. Given the huge commercial investment in theranostic agents in nuclear medicine and oncology,^32^ developing new efficient chelators for La radioisotopes is key to successfully support both therapeutic and diagnostic uses in precision medicine.

**Chart 1:**
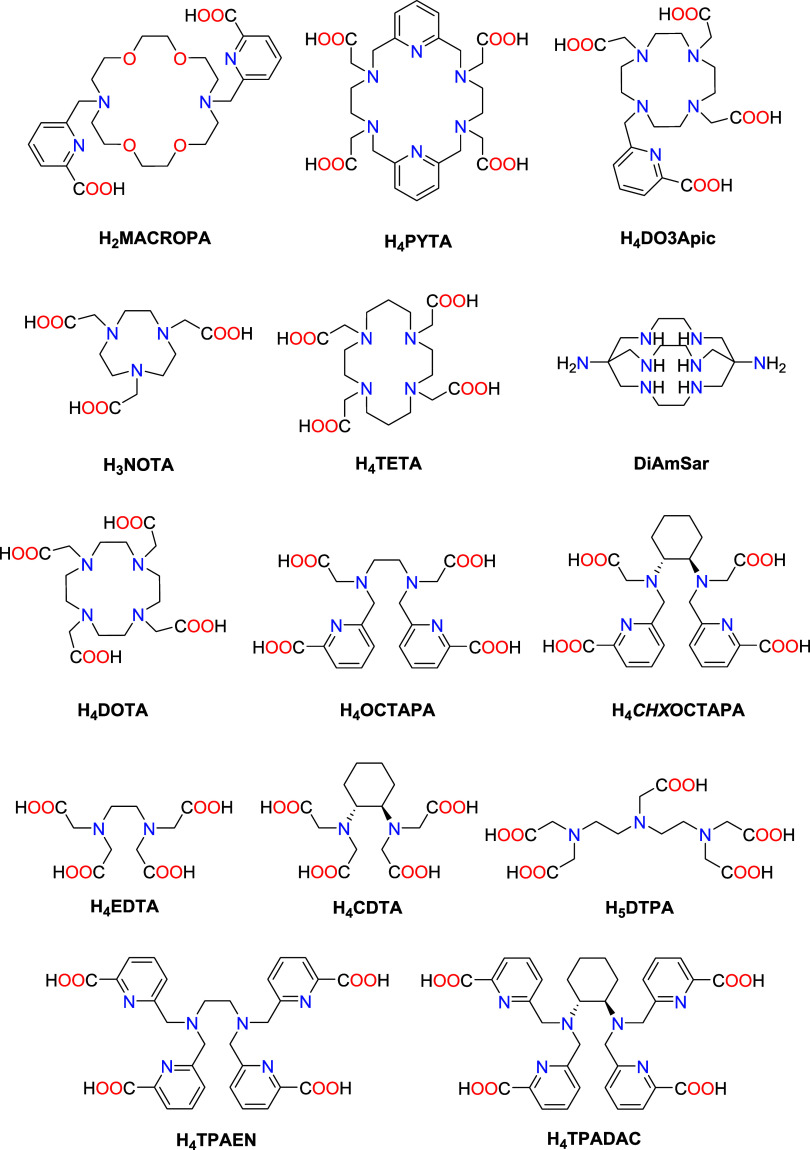
Chelators Discussed in This Work

The octadentate acyclic ligand H_4_OCTAPA has been established as an interesting candidate for the design of ^177^Lu, ^90^Y and ^111^In radiopharmaceuticals.^33–38^ Furthermore, the lanthanide complexes of the rigidified derivative H_4_ *CHX*OCTAPA were found to be considerably more inert with respect to complex dissociation than the corresponding complexes with H_4_OCTAPA.^39,40^ Considering the large ionic radii of La^3+^ and Ac^3+^, we sought to modify these acyclic chelators to incorporate up to ten donor atoms. This lead us to identify H_4_TPAEN, first reported by Mazzanti for the formation of highly luminescent Eu^3+^ and Tb^3+^ complexes, as a potential candidate.^41^ Furthermore, we envisaged that the rigidified H_4_TPADAC chelator could increase the inertness of the complexes, as observed for H_4_OCTAPA derivatives of the small Ln^3+^ ions (Gd^3+^ and Lu^3+^).^39,40^ This chelator was recently described by Adewuyi et al. to prepare circularly polarized Ln^3+^ emitting complexes.^42^ However, the coordination chemistry of La^3+^ with H_4_TPAEN and H_4_TPADAC and their potential for the development of radiopharmaceuticals remains unexplored.

Herein, we provide a detailed investigation on the coordination chemistry of H_4_TPAEN and H_4_TPADAC with La^3+^, including a detailed structural study using NMR spectroscopy and single-crystal X-ray diffraction. We also report a thermodynamic study that afforded the stability constants of the complexes, as well as an investigation of their dissociation kinetics. Finally, we present ^135^La radiolabeling studies as well as *in vitro* and *in vivo* stability studies, which support further development of these chelators for La^3+^-based radiopharmaceuticals.

## Results and Discussion

### Synthesis and Characterization of the Chelators

The preparation of ligands H_4_TPAEN and H_4_TPADAC was achieved by alkylation of the amine precursors with 6-(chloromethyl)­picolinic acid, using slight modifications of the literature procedures (Scheme S1, Supporting Information).^41,42^

Ligand protonation constants were determined in 0.15 M NaCl using potentiometric titrations (Figures S1–S2, Supporting Information). The results are compared with those reported previously for OCTAPA^4–^ and *CHX*OCTAPA^4–^ in Table 1, while the speciation diagrams are shown in Figure S3 (Supporting Information). The first two protonation constants of these ligands, characterized by log *K*_1_^H^ and log *K*_2_^H^, correspond to the protonation of amine groups, while the remaining protonation constants are ascribed to the carboxylate groups of picolinate and acetate moieties.^33,43,44^

**Table 1:** Protonation Constants of TPAEN^4–^ and TPADAC^4–^ ligands (*I* = 0.15 M NaCl, 25 °C) and Values Reported in the Literature for Related Ligands

	**TPAEN** ^**4‑**^	**TPADAC** ^**4‑**^	**OCTAPA** ^**4‑**^	***CHX*** **OCTAPA** ^**4‑**^	**DTPA** ^**5‑**^ ^e^	**EDTA** ^**4‑**^ ^f^	**CDTA** ^**4‑**^ ^f^
log *K*_1_^H^	7.87(1)	10.79(1)	8.52^a^/8.58^b^	9.52^c^/9.23^d^	9.93	9.17	9.36
log *K*_2_^H^	5.14(1)	4.95(1)	5.40^a^/5.43^b^	5.51^c^/5.40^d^	8.37	5.99	5.95
log *K*_3_^H^	3.89(1)	4.06(1)	3.65^a^/3.75^b^	3.99^c^/3.94^d^	4.18	2.73	3.62
log *K*_4_^H^	3.28(1)	3.46(1)	2.97^a^/3.08^b^	3.43^c^/2.24^d^	2.71	2.01	2.57
log *K*_5_^H^	2.55(1)	2.94(1)	1.66^a^/2.21^b^	1.59^c^/1.82^d^	2.00	1.38	1.49
log *K*_6_^H^	0.89(8)	0.67(6)	1.61^b^	0.61^c^/1.91^d^			
Σ log *K*_i_^H^ (*i* = 1–5)	22.73	26.20	22.20	24.04	27.19	21.27	22.99

a Data from ref 43.

b Data from ref 33.

c Data from ref 40.

d Data from ref 34.

e Data from ref 50.

f Data from ref 51.

This series of ligands present significant differences in the values of the first protonation constant log *K*_1_^H^, which involves one of the amine N atoms. The replacement of acetate groups of EDTA^4–^ by picolinate pendant arms provokes a decrease of log *K*_1_^H^, an effect that has been attributed to the stronger electron withdrawing effect of the picolinate units compared with acetate groups.^44^ Thus, the log *K*_1_^H^ values follow the order EDTA^4–^ > OCTAPA^4–^ > TPAEN^4–^. A comparison of the first protonation constants reported for EDTA^4–^ and CDTA^4–^ indicates that the cyclohexyl derivative possesses a slightly higher log *K*_1_^H^ value (Δlog *K*_1_^H^ = 0.19), which can be attributed to the inductive effect of the added alkyl groups.^45^ This effect is more pronounced (Δlog *K*_1_^H^ = ∼1.0) when comparing OCTAPA^4–^ with its cyclohexyl analogue, and then increases dramatically for the decadentate derivatives (Δlog *K*_1_^H^ = 2.9). However, all remaining protonation constants determined for TPAEN^4–^ and TPADAC^4–^ are rather similar. This suggests that the monoprotonated form of TPADAC^4–^ is very likely stabilized by intramolecular hydrogen bonds involving the protonated amine N atom and heteroatoms of the picolinate groups. The second protonation constant of both chelators, characterized by log *K*_2_^H^, can be attributed to the protonation of the second amine nitrogen atom, while the remaining protonation processes involve the carboxylate groups of the picolinate units. This is confirmed by the X-ray structure of [H_5_TPAEN]­Cl·3H_2_O, which evidence protonation of the amine N atoms and three carboxylate groups (Figure 1). A rather intricate network of hydrogen bonds involves the amine NH groups, carboxylate groups, water molecules and chloride anions.

**Figure 1:**
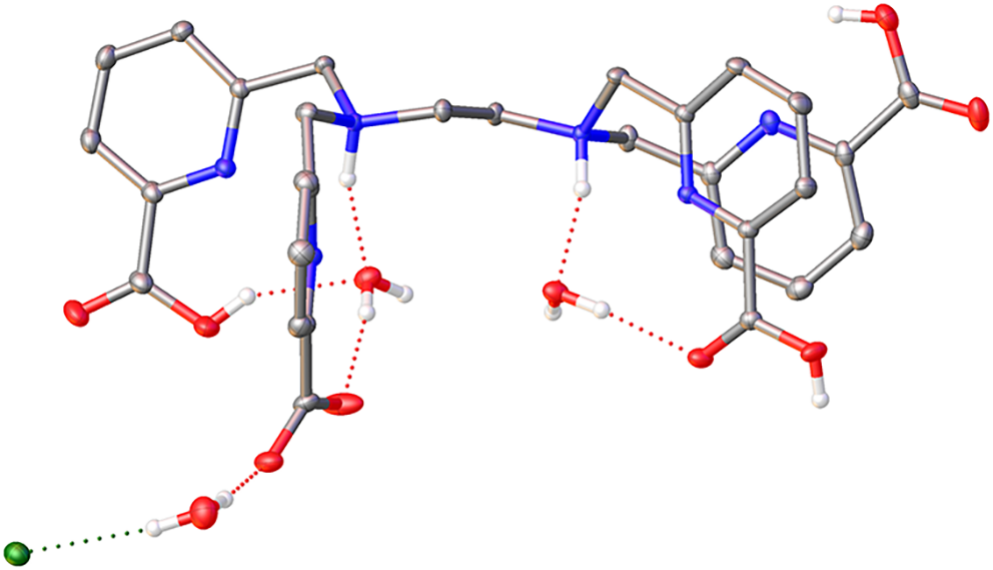
X-ray crystal structure of [H_5_TPAEN]­Cl·3H_2_O plotted with ellipsoids at the 50% probability level. Hydrogen atoms bonded to C atoms are omitted for simplicity.

The ^1^H and ^13^C NMR spectra of H_4_TPAEN recorded at acidic pH (pD = 2.8) are well-defined, displaying five and eight signals, respectively (Figures S4–S9, Supporting Information). In contrast, the ^1^H and ^13^C NMR spectra recorded for H_4_TPADAC at 298 K (pD = 2.2) display broad signals characteristic of fluxional behavior (Figures S10–S17, Supporting Information). The ^13^C NMR spectrum recorded at 278 K displays duplicate signals for the picolinate groups, pointing to a *C*_2_ symmetry of the ligand in which picolinate groups are equivalent in pairs (Figure 2). These signals broaden on increasing temperature, reach coalescence and finally become sharp at 338 K once the fast exchange regime is reached. At high temperature the ^13^C NMR spectrum displays 10 signals, with the four picolinate groups being equivalent. This suggests that the presence of intramolecular hydrogen bonds and the rigidity of the cyclohexyl spacer introduces a significant energy barrier for the inversion of the amine N atoms.^46–49^ This is confirmed by the ^1^H NMR spectrum recorded at high pH (pD = 12.8), in which the four picolinate units are magnetically equivalent (Figure S16, Supporting Information).

**Figure 2:**
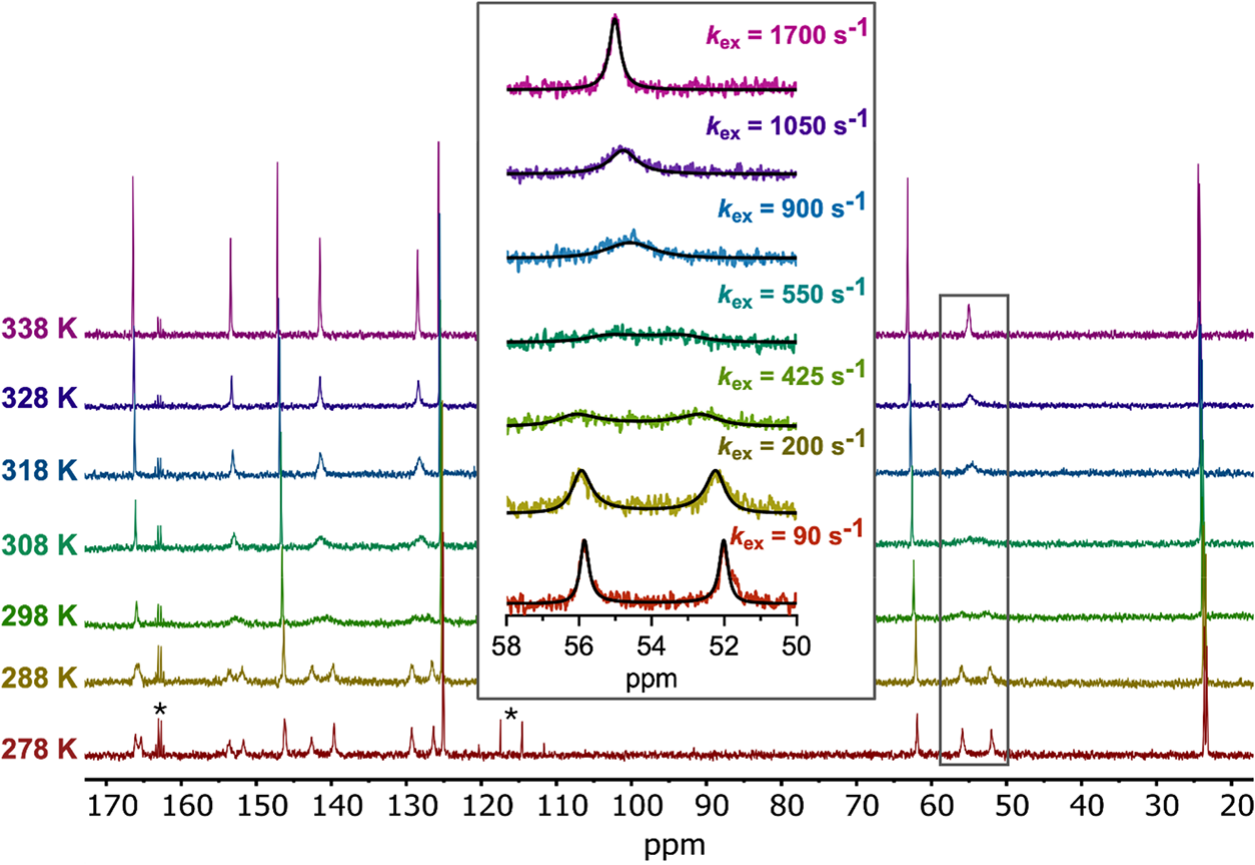
^13^C NMR spectra recorded for H_4_TPADAC in D_2_O solution (pD = 2.2, 100.6 MHz) at different temperatures. The inset shows the line width analysis of the resonances of the methylenic protons of the picolinate groups and the fits of the data to determine the rate constants (*k*_ex_) for the amine interconversion process. The asterisk denotes signals due to residual trifluoroacetic acid.

The two signals observed for the methylenic protons of the picolinate groups are observed at 52.0 and 55.9 ppm at 278 K and pD 2.2. The dynamic process observed for these signals is a simple interconversion between two sites with equal populations. Thus, rate constants for amine N atom inversion were obtained by line width analysis in the temperature range 278–338 K (Figure 2). The rate constants were subsequently used to determine the activation parameters for the interconversion process using an Eyring plot (Figure S18, Supporting Information). This analysis afforded values of the enthalpy and entropy of activation of Δ*H* ^‡^ = 33.8 ± 2.8 kJ mol^–1^ and Δ*S* ^‡^ = −83.8 ± 6.7 J mol^–1^ K^–1^, which indicates that the interconversion process has a significant entropy barrier. The activation free energy at 298.15 K and the rate constant at this temperature take values of Δ*G*_298_^‡^ = 58.8 ± 3.4 kJ mol^–1^ and *k*_ex_^298^ = 310 ± 18 s^–1^, respectively.

### Structural Characterization of the Complexes

The structures of the La^3+^ complexes were investigated using single crystal X-ray diffraction measurements. Single crystals with formula [La­(HTPAEN)]·12H_2_O were grown from a solution of the complex in methanol (Figure 3). Crystals with formula [LaCl­(H_2_O)_3_]­[La­(TPADAC)]­Cl·3H_2_O were obtained from an aqueous solution of the complex containing excess La^3+^. They contain the [La­(TPADAC)]^−^ complex and La^3+^ ions coordinated to three water molecules, a chloride anion and four oxygen atoms of carboxylate groups of four [La­(TPADAC)]^−^ entities (see Figures 3 and S20, Supporting Information).

**Figure 3:**
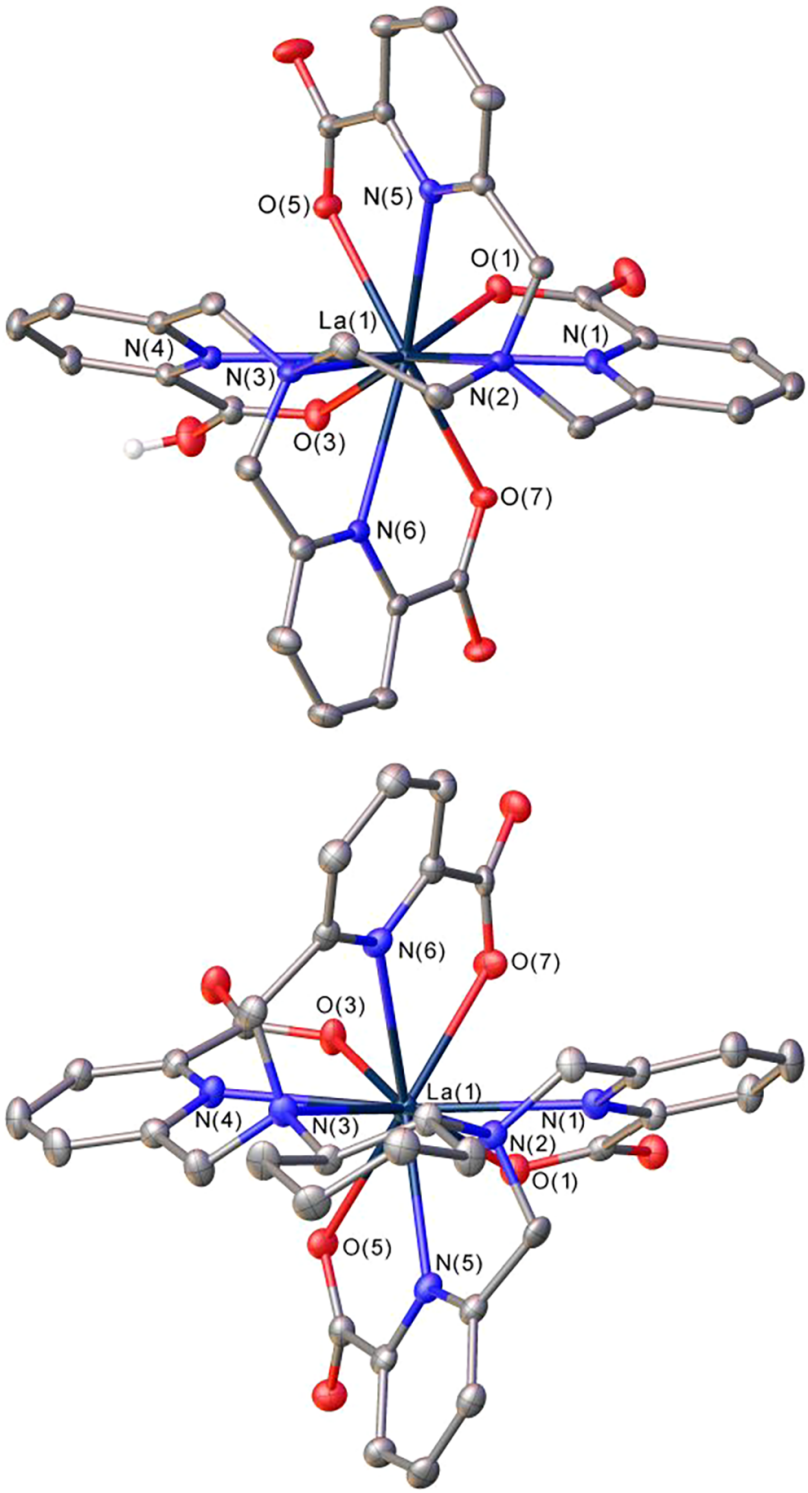
Views of the X-ray structures of the [La­(HTPAEN)] (top) and [La­(TPADAC)]^−^ (bottom) complexes with ellipsoids plotted at the 50% probability level.

The La^3+^ ions in both [La­(HTPAEN)] and [La­(TPADAC)]^−^ complexes are directly coordinated to the ten donor atoms of the ligand, which in the former is protonated on one of the carboxylate groups. The four picolinate groups of the ligand wrap around the pseudo *C*_2_ axis that contains the La^3+^ ion and bisects the C—C bond of the central ethylenediamine (or cyclohexyldiamine) spacer, giving rise to two possible helical forms that are often denoted as Δ and Λ.^52^ The Δ *c*onfiguration is associated with positive values of the four N—C—C—N dihedral angles involving the picolinate groups, while negative values indicate a Λ configuration.^53^ Furthermore, the formation of the five-membered chelate ring upon coordination of the two amine N atoms generates a second source of helicity, which can be described as δ or λ.^54^ The [La­(HTPAEN)] complex crystallizes in the centrosymmetric *P̅*1 triclinic space group, and thus the Δ­(λ) and Λ­(δ) enantiomers are present in the crystal lattice related by an inversion center. Compound [LaCl­(H_2_O)_3_]­[La­(TPADAC)]­Cl·3H_2_O crystallizes in the chiral monoclinic *P*2_1_ space group, and thus only the Δ­(λ) enantiomer is present in the crystal lattice, a situation imposed by the use of the enantiomerically pure (1*R*,2*R*)-cyclohexane-1,2-diamine precursor for the ligand synthesis.

The bond distances of the La^3+^ coordination environments in the [La­(HTPAEN)] and [La­(TPADAC)]^−^ complexes are presented in Table 2. The number of X-ray structures reported for La^3+^ complexes with picolinate ligands is rather scarce,^27,55,56^ with very few structures containing ten-coordinated La^3+^ ions.^26,57^ Thus, we analyzed the bond distances of the La^3+^ coordination environments by estimating standard values using the crystal radius of La^3+^ for coordination number ten (CR_La_ = 1.41 Å)^58^ and the donor radii reported recently for the rare earths (*r*_D_).^59^ This provides reference values calculated as *r*_D_ + CR_La_ that can be used to compare with experimental data. The differences between experimental and reference values (Δ*d*) obtained for [La­(HTPAEN)] are small, with absolute values being <0.05 Å for all donor atoms except O(3), which involves a protonated carboxylate group. The situation is very different for [La­(TPADAC)]^−^, which shows large positive values for the amine N atoms. This indicates that the TPAEN^4–^ ligand is better suited to adapt to the coordination requirements of the La^3+^ ion than TPADAC^4–^, a situation that appears to be related to the smaller bite distance (N(2)···N(3) = 2.972 Å) and bite angle (N(2)—La(1)—N(3) = 60.48(13)°) of the central chelate ring in [La­(TPADAC)]^−^ when compared with [La­(HTPAEN)] (N(2)···N(3) = 3.002 Å; N(2)—La(1)—N(3) = 63.16(9)°).

**Table 2:** Bond Distances (Å) of the La^3+^ Coordination Environments in the X-ray Structures of [La­(HTPAEN)] and [La­(TPADAC)]^−^ (*d*_La‑D_) Compared with Those Estimated from Donor Radii and Crystal Radii (*r*_D_ + CR_La_) and the Differences between Them Δ*d*

		*d*_La‑D_			Δ*d* ^a^	
donor type		TPAEN	TPADAC	*r*_D_ + CR_La_	TPAEN	TPADAC
carboxylate	La(1)—O(1)	2.498(3)	2.552(4)	2.542	–0.044	0.010
	La(1)—O(3)	2.617(3)	2.532(4)	2.542	0.075	–0.010
	La(1)—O(5)	2.547(3)	2.518(4)	2.542	0.005	–0.024
	La(1)—O(7)	2.537(3)	2.488(4)	2.542	–0.005	–0.054
amine	La(1)—N(2)	2.854(3)	2.968(5)	2.835	0.019	0.133
	La(1)—N(3)	2.878(3)	2.933(4)	2.835	0.043	0.098
pyridine	La(1)—N(1)	2.694(3)	2.699(5)	2.739	–0.045	–0.040
	La(1)—N(4)	2.770(3)	2.722(5)	2.739	0.031	–0.017
	La(1)—N(5)	2.734(3)	2.708(5)	2.739	–0.005	–0.031
	La(1)—N(6)	2.701(3)	2.691(5)	2.739	–0.038	–0.048

a Δ*d* = *d*_La‑D_ – (*r*_D_ + CR_La_), and thus negative values indicate that the observed distances (*d*_La‑D_) are shorter than the reference values (*r*_D_ + CR_La_).

The five-membered chelate rings formed upon coordination of the pyridine groups in rare-earth complexes display dihedral angles N—C—C—N with ideal values of 32.0°.^60^ The [La­(HTPAEN)] complex shows dihedral angles that are relatively close to the ideal value (30.7, 32.6, 24.7 and 35.8°), while rather large deviations are observed in [La­(TPADAC)]^−^ for two of the dihedral angles (46.3 and 43.2°). This again suggests that the ethylenediamine spacer is better suited for La^3+^ coordination than the rigid cyclohexane-1,2-diamine unit.

The structure of the [La­(TPAEN)]^−^ and [La­(TPADAC)]^−^ complexes in solution was investigated using ^1^H and ^13^C NMR spectroscopy (Figures S21–S29, Supporting Information). The ^1^H NMR spectrum of [La­(TPAEN)]^−^ displays 12 signals, a situation that is consistent with an effective *C*_2_ symmetry of the complex in solution (Figure 4). This is confirmed by the ^13^C NMR spectrum, which displays 15 signals for the 30 carbon nuclei of the ligand backbone. Both the ^1^H and ^13^C NMR spectra are well resolved, with the methylene protons observed as AB spin systems. This indicates that the complex is rather rigid in solution, with a single diastereoisomer being present. The ^1^H NMR spectrum recorded for [La­(TPADAC)]^−^ is also consistent with a *C*_2_ symmetry, although some broadening is observed. The full assignment of the ^1^H and ^13^C NMR spectra is presented in the Supporting Information (Tables S3–S4, Supporting Information).

**Figure 4:**
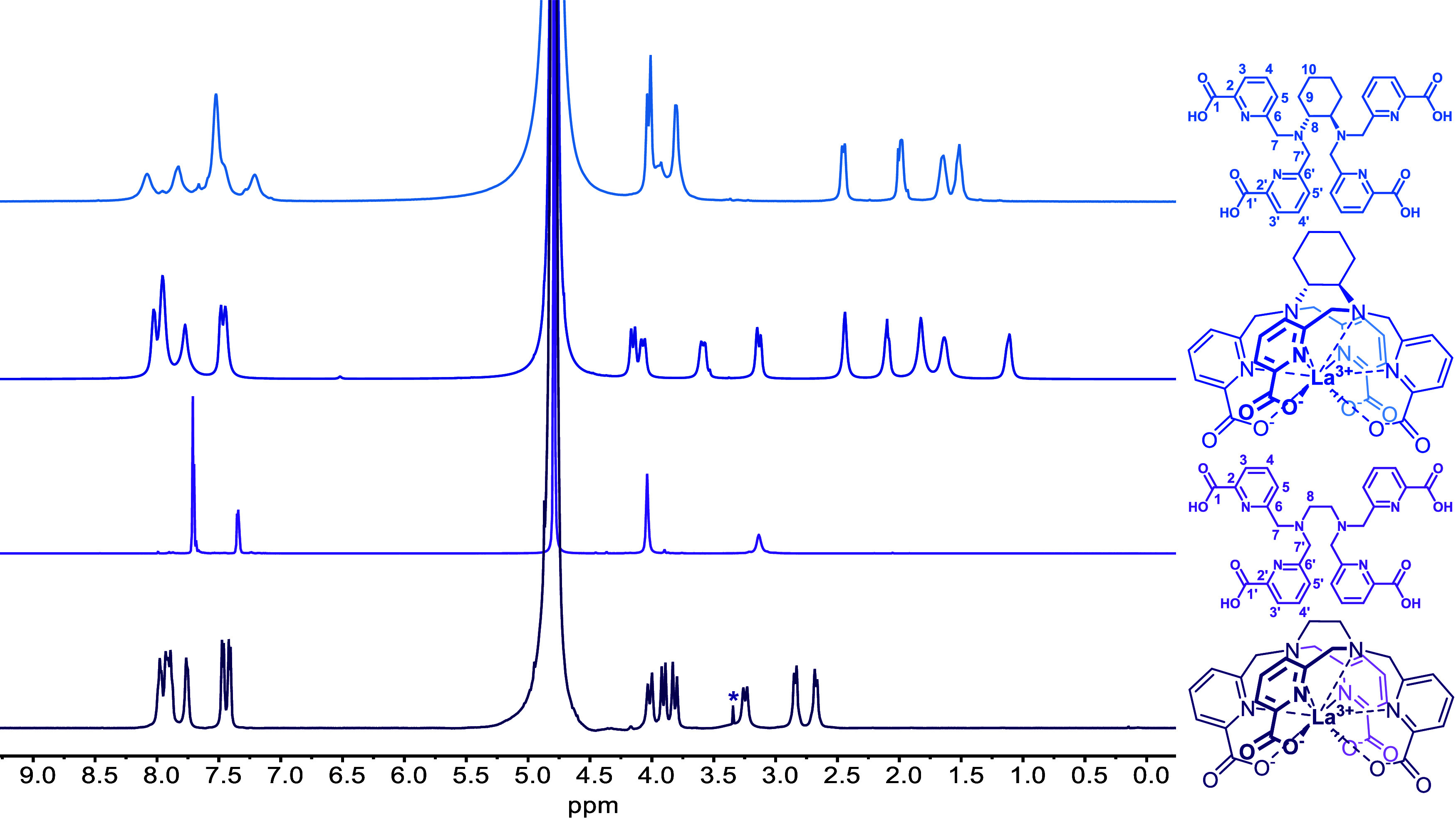
^1^H NMR spectra (500 MHz) of the H_4_TPAEN (pD = 7.4) and H_4_TPADAC (pD = 7.8) and their La^3+^ complexes recorded in D_2_O solution at 298 K. The asterisk denotes residual methanol.

### Stability Constants

The stability constants of the La^3+^ complexes were determined using pH potentiometry (*I* = 0.15 NaCl). A significant amount of the metal ion is not complexed by the TPADAC^4–^ ligand at the beginning of the titration at pH 1.7 (∼20%), which allows for stability constant determination using potentiometry. In the case of TPAEN^4–^ the amount of free La^3+^ was small (∼4%), but the stability constants could be determined by fitting the titration data using both 1:1 and 1:2 (TPAEN^4–^:La^3+^) stoichiometries (Figure S1, Supporting Information). The TPADAC^4–^ ligand also forms complexes with 1:1 and 1:2 stoichiometries (Figure S2, Supporting Information). The fits of the titration curves improved significantly by including a protonated form of the complexes and a hydroxo-complex in the equilibrium model. The fits of the potentiometric data afforded the equilibrium constants reported in Table 3.

**Table 3:** Stability Constants of the La^3+^ Complexes with TPAEN^4–^ and TPADAC^4–^ (*I* = 0.15 M NaCl, 25 °C) and Values Reported in the Literature for Related Ligands

	**TPAEN** ^**4‑**^	**TPADAC** ^**4‑**^	**OCTAPA** ^**4‑**^ ^a^	***CHX*** **OCTAPA** ^**4‑**^ ^b^	**DTPA** ^c^	**MACROPA** ^**2‑**^	**DO3Apic** ^**4‑**^ ^f^
log *K*_LaL_	19.16(8)	19.55(1)	19.92	17.82	19.36	14.99;^d^ 13.9^e^	21.17
log *K*_LaL_^H^	2.02(9)	2.26(1)		2.00		2.28^d^	2.55
log *K*_LaL_^OH^	12.62(9)	12.06(1)		12.75			
log *K*_LaL_^La^	2.31(13)	2.85(1)					
log *K*_La_2_L_^OH^	6.80(10)	6.99(4)					
log *K*_La_2_LOH_^OH^	9.57(10)	9.40(4)					
pLa^g^	19.5	17.1	19.7	16.6	16.2	15.6	16.6

a Data in 0.15 M NaCl from ref 43.

b Data in 0.15 M NaCl from ref 39.

c Data in 0.1 M Na­(ClO_4_) from ref 62.

d Data in 0.1 M KCl from ref^64^.

e Data in 0.1 M NaCl from ref 65.

f Data in 0.1 M KCl from ref 63.

g Calculated as −log­[La^3+^]_free_ for [L] = 10 μM and [La^3+^] = 1 μM at pH 7.4 and 25 °C.

The speciation diagrams obtained using the equilibrium data indicate that the dissociation of the complex with TPAEN^4–^ occurs at slightly lower pH (<∼2.5) than that of TPADAC^4–^ (<∼3.0). Hydroxo-complex formation takes place at rather high pH (>9.0) in both cases, with the negatively charged [La­(TPAEN)]^−^ and [La­(TPADAC)]^−^ complexes being the only species present in solution around physiological pH values (Figure 5). As mentioned above, the TPAEN^4–^ and TPADAC^4–^ ligands form weak 1:2 L:La^3+^ complex species that are characterized by the equilibrium constants shown in Table 3. Additional speciation diagrams for a 1:2 (L:La^3+^) stoichiometry are shown in Figure S30 (Supporting Information). The formation of these 1:2 L:La^3+^ complexes is likely related to the presence of bridging carboxylate units that connect La^3+^ ions inside and outside the cage of the ligand, as observed for phosphonate derivatives.^61^

**Figure 5:**
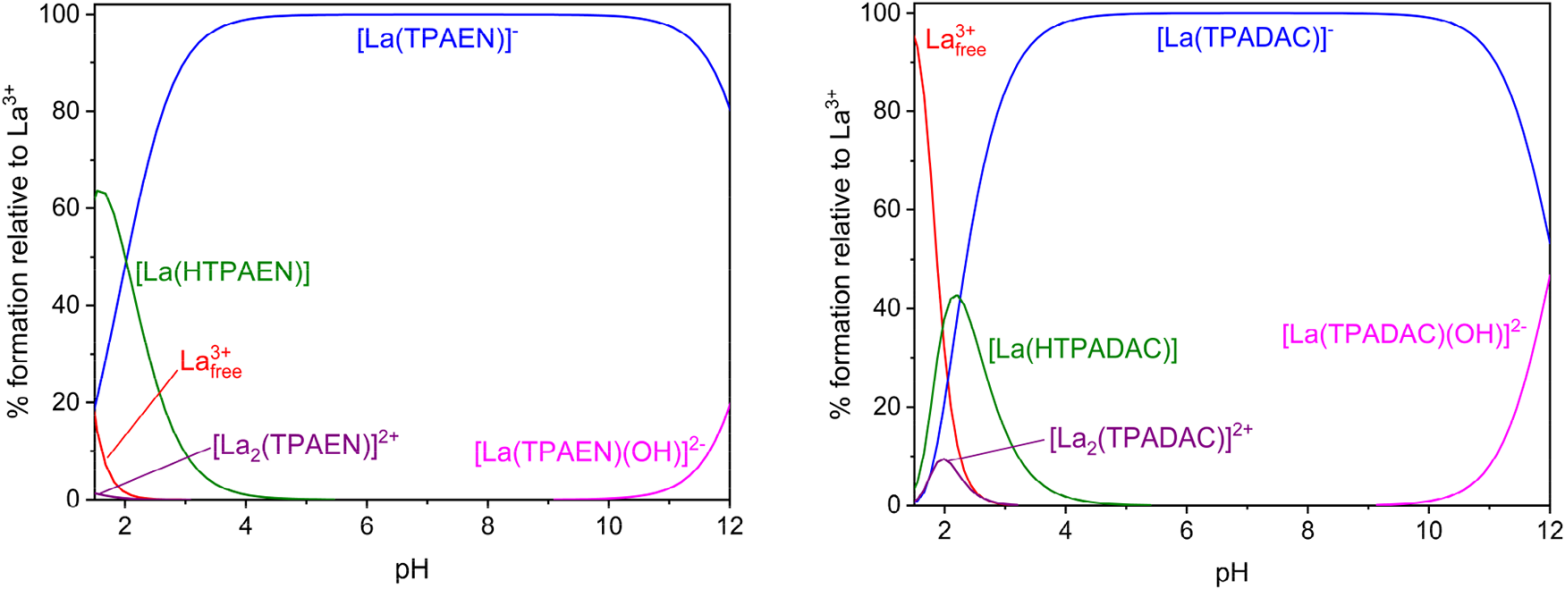
Speciation diagrams of the La^3+^:TPAEN^4–^ (left) and La^3+^:TPADAC^4–^ (right) systems, calculated for c_La3+_ = c_Lig_ = 10^–3^ M (*I* = 0.15 M NaCl, 25 °C).

The stability constants (log *K*_LaL_) determined for the La^3+^ complexes of TPAEN^4–^ and TPADAC^4–^ are comparable to those reported for OCTAPA^4–^,^43^ DTPA^5–^ ^62^ and DO3Apic^4–^ ^63^ and significantly higher than that of MACROPA^2–^ (Table 3).^64^ The log *K*_LaL_ value determined for TPADAC^4–^ is also significantly higher than that reported previously for *CHX*OCTAPA^4–^, while those of TPAEN^4–^ and OCTAPA^4–^ are very similar. Thus, increasing the denticity of the ligand in the cyclohexyl derivatives leads to a considerably improvement of the stability constant. This effect appears to be related to a poor fit of the “bite” of the 1,2-cyclohexyldiamine unit and the large La^3+^ ion (see above). The complexes of TPAEN^4–^ and TPADAC^4–^ form protonated complex species at low pH due to the protonation of a picolinate group (log *K*_LaL_^H^ ∼ 2), as evidenced by the X-ray structure of [La­(HTPAEN)] described above. Protonated forms characterized by similar protonation constants were also detected in the case of picolinate derivatives *CHX*OCTAPA^4–^, MACROPA^2–^ and DO3Apic^4–^. The complexes of TPAEN^4–^ and TPADAC^4–^, as well as that of *CHX*OCTAPA^4–^, form hydroxo-complexes at high pH (pH > 10.5 and 9.5, respectively).

The thermodynamic stability of the complexes at physiologically relevant pH was assessed by calculating pLa values, defined as −log­[La^3+^]_free_ for [L] = 10 μM and [La^3+^] = 1 μM at pH 7.4 and 25 °C following the recommendation of Raymond.^66^ TPAEN^4–^ and OCTAPA^4–^ display the highest pLa values among the ligands listed in Table 3. This is a result of the high stability constants (log *K*_LaL_) that characterize the formation of these complexes, as well as the relatively low basicity of these ligands. Indeed, cyclohexyl derivatives TPADAC^4–^ and *CHX*OCTAPA^4–^ display considerably higher basicity than the ethyl analogues, as indicated by the Σ log *K*_i_^H^ (*i* = 1–5) values shown in Table 1. Similarly, DTPA and DO3A derivatives are characterized by rather high basicities, which results in relatively low pLa values.^50^

### Dissociation Kinetics

The inertness of metal complexes for radiopharmaceutical applications is important to ensure that the radioisotope is not released *in vivo* off-target. Furthermore, the detailed study of the dissociation mechanism of the complexes provides useful information for ligand design. Thus, we performed a detailed investigation of the dissociation kinetics of the [La­(TPAEN)]^−^ and [La­(TPADAC)]^−^ complexes using Cu^2+^ as a scavenger. The reactions were studied spectrophotometrically in the approximate proton concentration range 1.1 × 10^–5^–1.6 × 10^–2^ M, which corresponds to a pH range of about 1.8–5.0. A large excess of Cu^2+^ was used to ensure pseudo-first order conditions. The observed rate constants (*k*_obs_) are shown in Figure 6.

**Figure 6:**
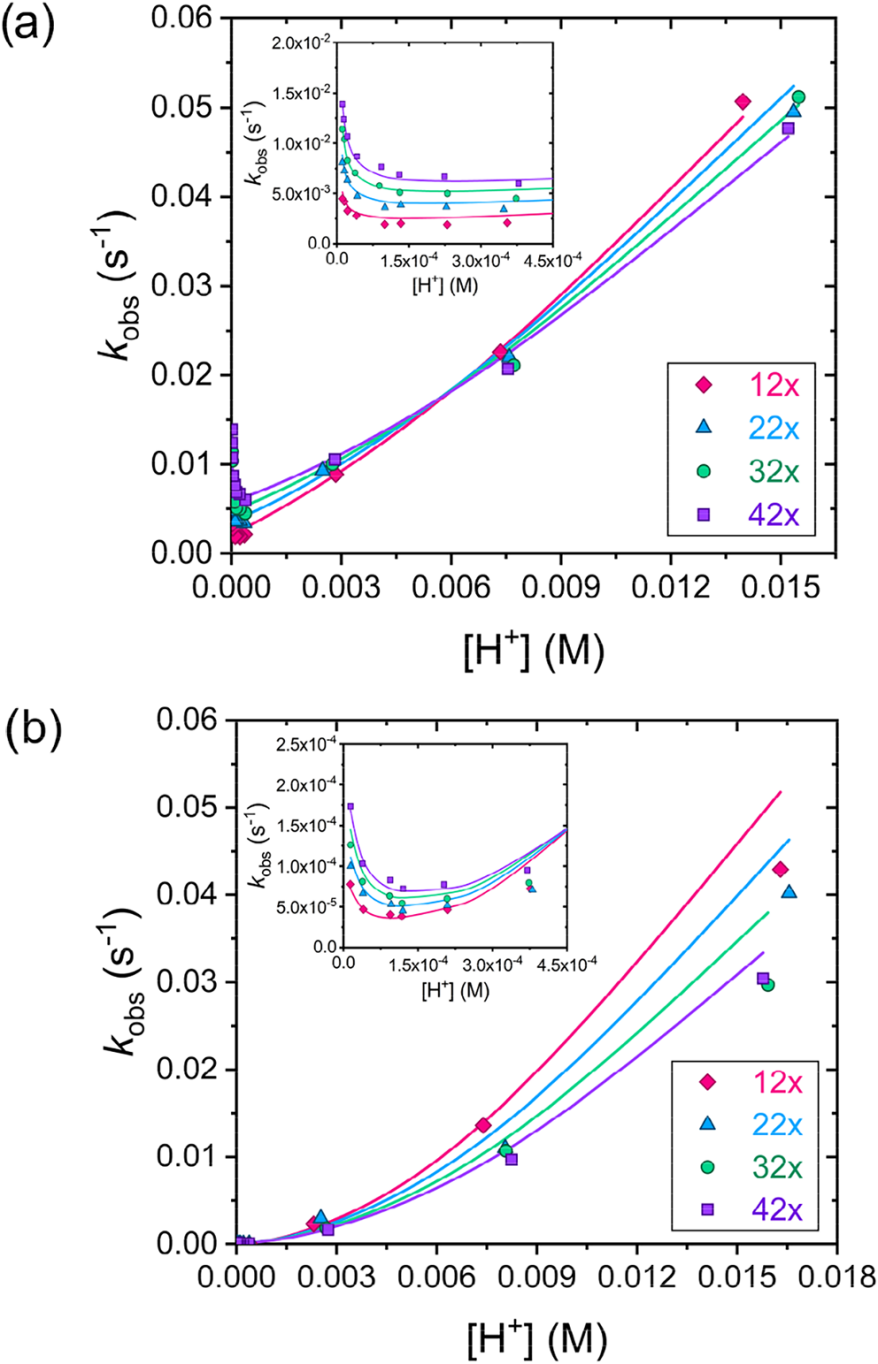
Pseudo-first order rate constants (*k*_obs_) obtained for the transmetalation reactions with Cu^2+^ of (a) [La­(TPAEN)]^−^ and (b) [La­(TPADAC)]^−^ as a function of the concentration of H^+^ ions (25 °C, 0.15 M NaCl, c_LaL_ = 0.25 mM, c_Cu2+_ = 2.98 mM (12×); 5.52 mM (22×), 7.98 mM (32×) and 10.52 mM (42×)). Insets: zoom of the proton concentration range 0–4.5 × 10^–4^ M. The solid lines represent the fits of the data (*R* ^2^ > 0.996).

The values of *k*_obs_ increase with increasing Cu^2+^ concentration in a proton concentration range of 1.1 × 10^–5^–3.7 × 10^–4^ M, indicating that the metal-assisted pathway, through the formation of a heterodinuclear (LaLCu) complex, contributes to the dissociation of the complexes characterized by rate constant *k*_3_^Cu^. Furthermore, the values of *k*_obs_ increase with decreasing [H^+^] below ∼0.1 mM (see insets in Figure 6), indicating that complex dissociation also occurs through the formation of a dinuclear hydroxo-complex, characterized by *k*_6_^Cu^. The dissociation rates increase with H^+^ concentration for [H^+^] > 0.1 mM, indicating dissociation through the proton-assisted pathway. The latter may proceed through the formation of mono- or diprotonated intermediates, with associated rate constants *k*_1_ and *k*_2_, respectively. The dissociation rates obtained under these conditions evidence a quadratic dependence on [H^+^], indicating that both *k*_1_ and *k*_2_ contribute significantly to the overall dissociation rates. We have recently observed a similar behavior for the [Lu­(*CHX*OCTAPA)]^−^ complex.^39^ Thus, we analyzed the kinetic data using the following expression:

1 $$k_{\mathrm{obs}}=\frac{k_{0}+k_{1}\left[H^{+}\right]+k_{2}{\left[H^{+}\right]}^{2}+k_{3}\left[{\mathrm{Cu}}^{2+}\right]+k_{6}\left[{\mathrm{Cu}}^{2+}\right]K_{w}/\left[H^{+}\right]}{1+K_{\mathrm{Cu}}\left[{\mathrm{Cu}}^{2+}\right]}$$

Here, *k*_1_, *k*_2_, *k*_3_^Cu^ and *k*_6_^Cu^ are the rate constants defined above, *k*_0_ is the rate constant characterizing the spontaneous dissociation mechanism, *K*_w_ is the ionic product of water (fixed at p*K*_w_ = 13.72) and *K*_Cu_ is the equilibrium constant for the formation of the ternary complex (*K*_Cu_= [LaLCu]/[Cu^2+^]­[LaL]). The least-squares fits of the kinetic data afforded the rate constants shown in Table 4 as well as the value of *K*_Cu_. The values of *k*_0_ obtained during the fitting procedure were negative or had very large errors, indicating that the spontaneous pathway does not contribute to complex dissociation under the conditions used for our kinetic experiments. This is not surprising considering that the dissociation through formation of a dinuclear hydroxo-complex plays an important role at the pH values in which spontaneous dissociation may contribute.

**Table 4:** Rate and Equilibrium Constants and Half-live Values Characterizing the Dissociation Reactions of [La­(TPAEN)]^−^ and [La­(TPADAC)]^−^ (25 °C, 0.15 M NaCl)

	**TPAEN** ^**4‑**^	**TPADAC** ^**4‑**^
*k*_1_ (M^–1^ s^–1^)	2.24 ± 0.13	0.097 ± 0.036
*k*_2_ (M^–2^ s^–1^)	110.93 ± 10.42	232.3 ± 36.8
*k*_3_^Cu^ (M^–1^ s^–1^)	0.74 ± 0.05	(7.8 ± 2.2)×10^–3^
*k*_6_^Cu^ (M^–2^ s^–1^)	(7.2 ± 0.5)×10^8^	(1.5 ± 0.3)×10^7^
*K*_Cu_ (M^–1^)	42.0 ± 4.5	74.3 ± 30.2
*k*_obs_ (s^–1^) for [Cu^2+^] = 1 μM^a^	3.45 × 10^–4^	7.381 × 10^–6^
*k*_obs_ (s^–1^) for [Cu^2+^] = 1 nM^a^	4.34 × 10^–7^	1.123 × 10^–8^
*t*_1/2_ (h) for [Cu^2+^] = 1 μM^a^	0.56	26.1
*t*_1/2_ (h) for [Cu^2+^] = 1 nM^a^	443.9	1.71 × 10^4^

a Calculated at pH= 7.4.

The rate constants shown in Table 4 evidence that [La­(TPAEN)]^−^ is more prone to dissociation than [La­(TPADAC)]^−^ following both the acid- and metal-assisted pathways. This is clearly reflected in the dissociation rates and half-lives calculated at pH 7.4. The half-live calculated using a 1 μM concentration of Cu^2+^ is ∼46 times longer for [La­(TPADAC)]^−^ than for [La­(TPAEN)]^−^, while this value is reduced to ∼38 using a [Cu^2+^] of 1 nM. However, it is important to note that Cu is strongly and inertly bound in blood, and even rather strong ligands cannot retrieve it from ceruloplasmin, to which copper is bound at ∼15 μM concentrations. A small fraction of the copper present in blood is bound to serum albumin (∼1 μM) with an association constant of ca. 10^13^, and thus strong chelators are required to release it.^67–69^ The low values of *K*_Cu_ determined from the fits of the kinetic data indicate a weak association of both [La­(TPADAC)]^−^ and [La­(TPAEN)]^−^, making the release of copper from blood proteins very unlikely. Thus, it is likely that the spontaneous and proton-assisted dissociation pathways are the most relevant mechanisms under physiological conditions.

### Radiolabeling Studies with ^135^La^3+^

H_4_TPAEN and H_4_TPADAC both demonstrated highly efficient incorporation of ^135^La^3+^, achieving radiochemical purities greater than 99% (*n* = 6) under optimized conditions. These conditions included using a 3 μM ligand concentration (H_4_TPAEN or H_4_TPADAC), a short reaction time of one minute, and maintaining the pH between 4 and 5 at room temperature. Under these settings, the reactions produced high molar activities of 299.4 MBq/nmol for H_4_TPAEN and 326.8 MBq/nmol for H_4_TPADAC. Adjustments such as increasing the ligand concentration or prolonging the reaction time at the same pH yielded comparable results (Figure 7), indicating that the process is robust. When reactions were carried out outside the optimal pH range, specifically at pH 3–4 and 5–6, the radiochemical purity dropped slightly to around 90% for both ligands. To reach the ideal pH window, the initial pH 2 ^135^La^3+^ solution was adjusted using a 1 M sodium acetate buffer at pH 9. Overall, these results confirm that both H_4_TPAEN and H_4_TPADAC enable rapid and effective radiolabeling under mild conditions, with minimal input material and time. The study highlights that pH, ligand concentration, and reaction time are key factors in radiolabeling efficiency, but that optimal labeling can be reliably achieved with simple, quick protocols. This represents a clear advantage of these acyclic chelators when compared with H_4_DOTA, which requires heating to 70 °C for 60 min to achieve a radiochemical purity of 97% (Figure S31, Supporting Information).

**Figure 7:**
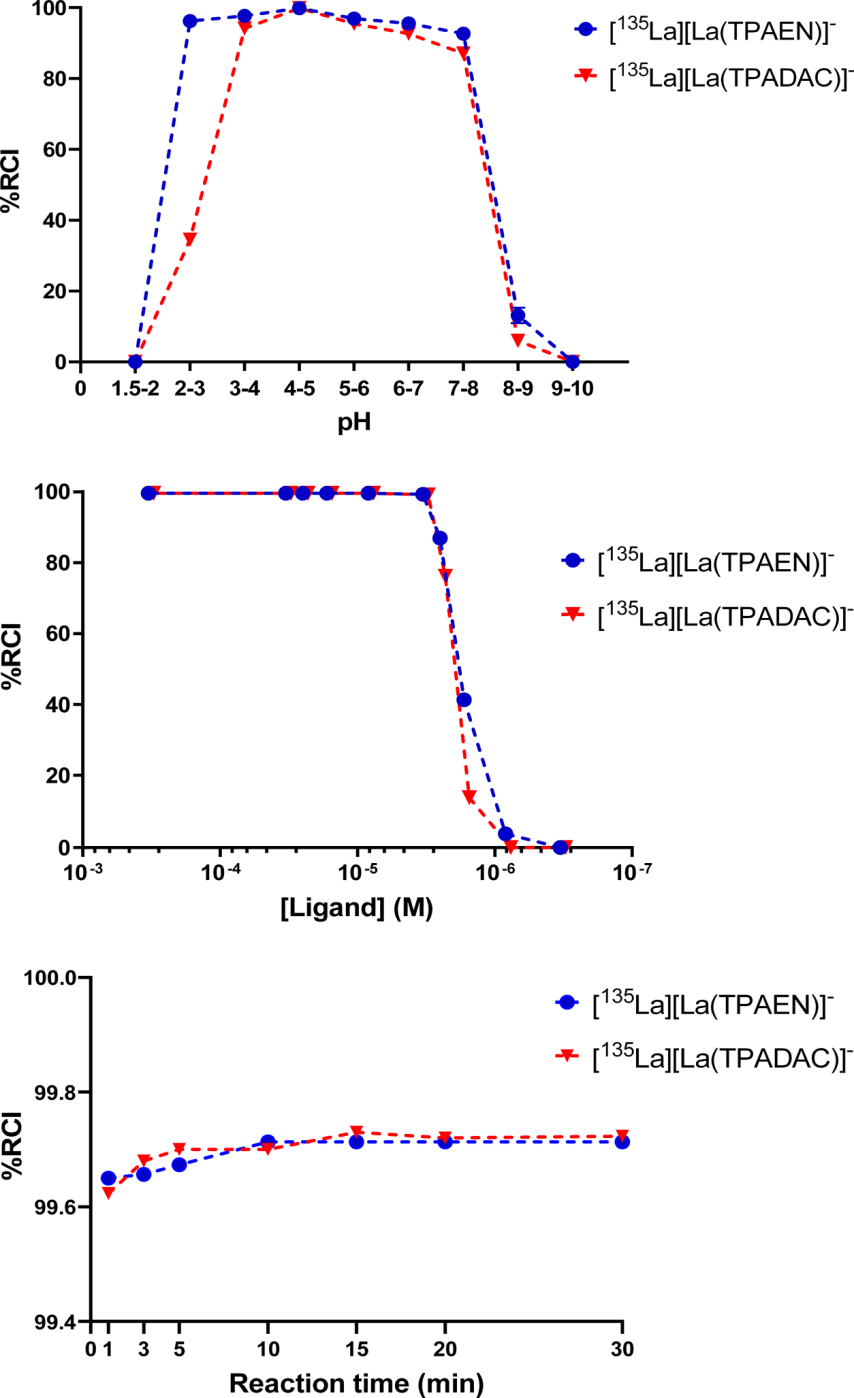
Labeling tests of both H_4_TPAEN and H_4_TPADAC with [^135^La]­La^3+^ carried out under different reaction conditions to set the “sweet spot”. Top: pH-dependency for 3 μM ligand concentration and 1 min of reaction time; Center: Concentration-dependency at 1 min of reaction time and pH 4–5; Bottom: Reaction time for a 3 μM ligand concentration and pH 4–5. (RCI: Radiochemical Incorporation).

### Stability Experiments

Both radiolanthanum complexes remained stable in whole mouse blood and human serum for up to 60 min. [^135^La]­[La­(TPAEN)]^−^ was stable across all tested buffer solutions. In contrast, [^135^La]­[La­(TPADAC)]^−^ showed reduced stability at pH 2.2, with radiochemical purity dropping to 60% after 1 h and to 8% after 48 h (Figure 8). Radio-TLC analysis of the degraded [^135^La]­[La­(TPADAC)]^−^ sample revealed two distinct peaks with different R_*f*_ values, indicating the formation of new radiolabeled compounds and degradation of the original complex.

**Figure 8:**
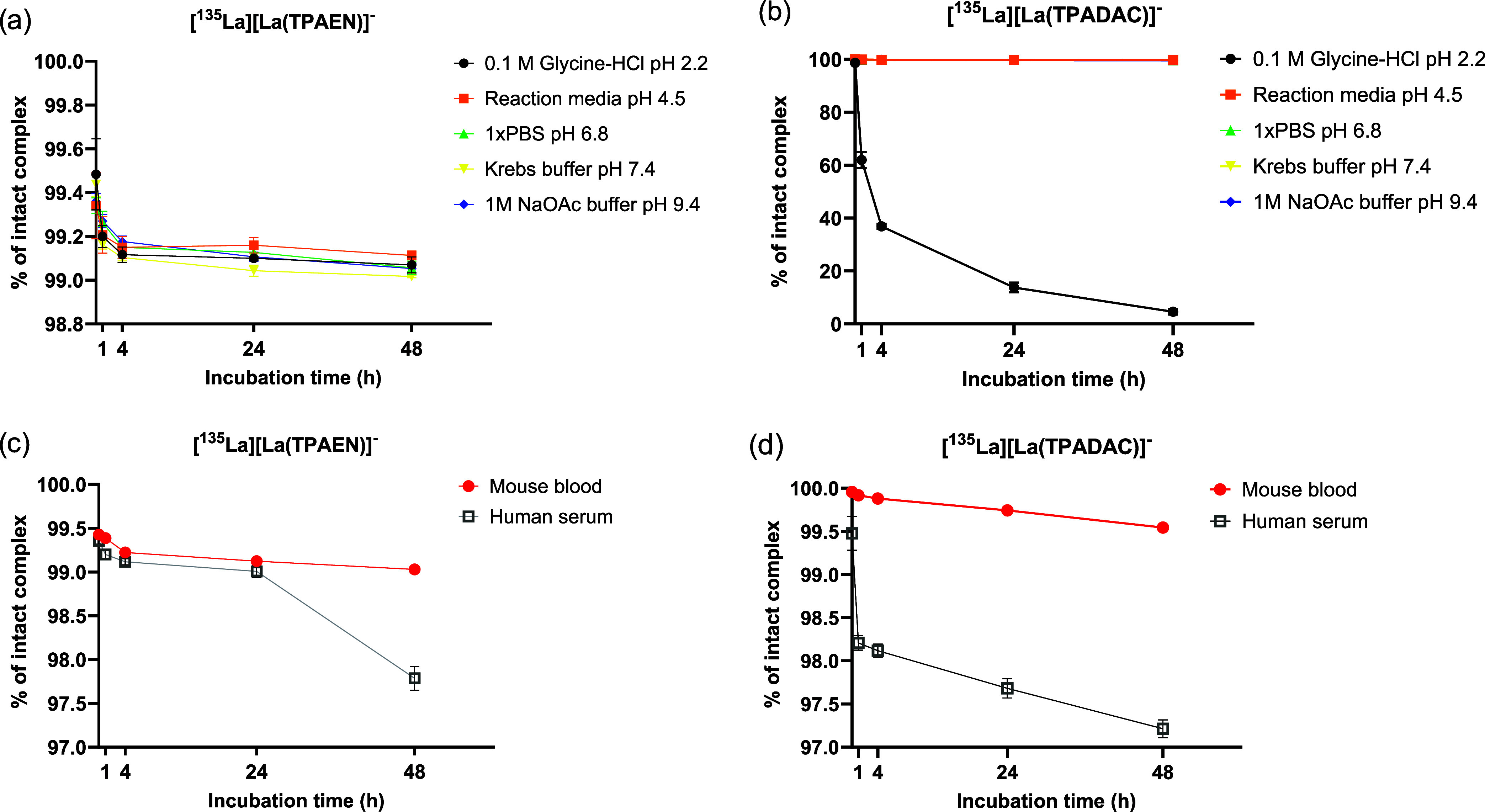
*In vitro* stability studies of both complexes in buffer solutions (a and b), and in human serum and mouse blood at 37 °C (c and d).

However, no free ^135^La was detected. The nature of these new compounds was not further investigated. These results suggest that while both complexes are stable in biological fluids, [^135^La]­[La­(TPADAC)]^−^ is sensitive to highly acidic conditions. This can be attributed to the lower thermodynamic stability of the TPADAC^4–^ complex when compared to [La­(TPAEN)]^−^, as indicated by the corresponding pLa values and the speciation diagrams shown in Figure 5.

### Challenge Experiments

To evaluate the stability of [^135^La]­[La­(TPAEN)]^−^ and [^135^La]­[La­(TPADAC)]^−^, both complexes were challenged with well-known macrocyclic and acyclic chelators: H_4_DOTA, H_3_NOTA, H_4_TETA, H_4_EDTA, DiAmSar, and H_2_MACROPA (Chart 1). H_4_DOTA and H_2_MACROPA have previously demonstrated efficient radiolabeling with radiolanthanum isotopes and served as benchmarks, while the other ligands were included to test thermodynamic stability. Radiolabeling results confirmed that both DOTA and MACROPA form stable complexes with ^135^La. [^135^La]­[La­(DOTA)] (R_*f*_ = 0.41) reached 90% radiochemical purity (*n* = 3) after 1 h at 37 °C, and 97% (*n* = 3) at 70 °C under the same time. [^135^La]­[La­(MACROPA)] (R_*f*_ = 0.347) achieved 99% purity (*n* = 3) after 5 min at 37 °C. In contrast, H_3_NOTA, H_4_EDTA, H_4_TETA, and DiAmSar did not form stable complexes with ^135^La under the tested conditions (Figure S31, Supporting Information). The radio-TLC system clearly distinguished the radiolabeled species, with [^135^La]­[La­(TPAEN)]^−^ and [^135^La]­[La­(TPADAC)]^−^ showing R_*f*_ values of 0.33 and 0.39, respectively—distinct from those of the DOTA and MACROPA analogues. In challenge experiments, both [^135^La]­[La­(TPAEN)]^−^ and [^135^La]­[La­(TPADAC)]^−^ maintained high radiochemical purity (up to 98%) after 120 min in the presence of excess competing ligands. No transchelation was observed, confirming the high kinetic and thermodynamic stability of these complexes in competitive environments (Figure 9).

**Figure 9:**
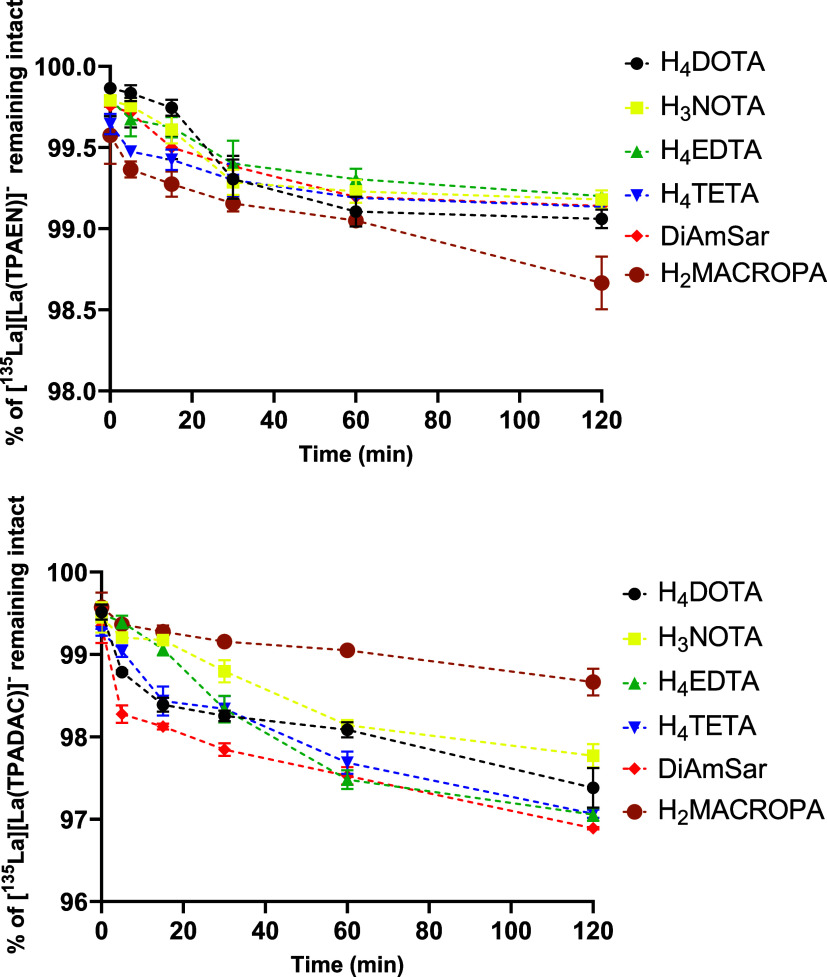
Challenge experiments with [^135^La]­[La­(TPAEN)]^−^ (top) and [^135^La]­[La­(TPADAC)]^−^ (bottom) using H_4_DOTA, H_3_NOTA, H_4_EDTA, H_4_TETA, DiAmSar and MACROPA.

### *In Vivo* Studies

*In vivo* stability of complexes [^135^La]­[La­(TPAEN)]^−^ and [^135^La]­[La­(TPADAC)]^−^ was studied in healthy mice. Blood compartment analysis (blood cells, plasma proteins, and supernatant) showed that both [^135^La]­[La­(TPAEN)]^−^ and [^135^La]­[La­(TPADAC)]^−^ were largely bioavailable in the supernatant, ranging from 60 to 80% similarly to [^135^La]­[La­(MACROPA)]^+^ (Figure 10). However, stability differed significantly between the two complexes.

**Figure 10:**
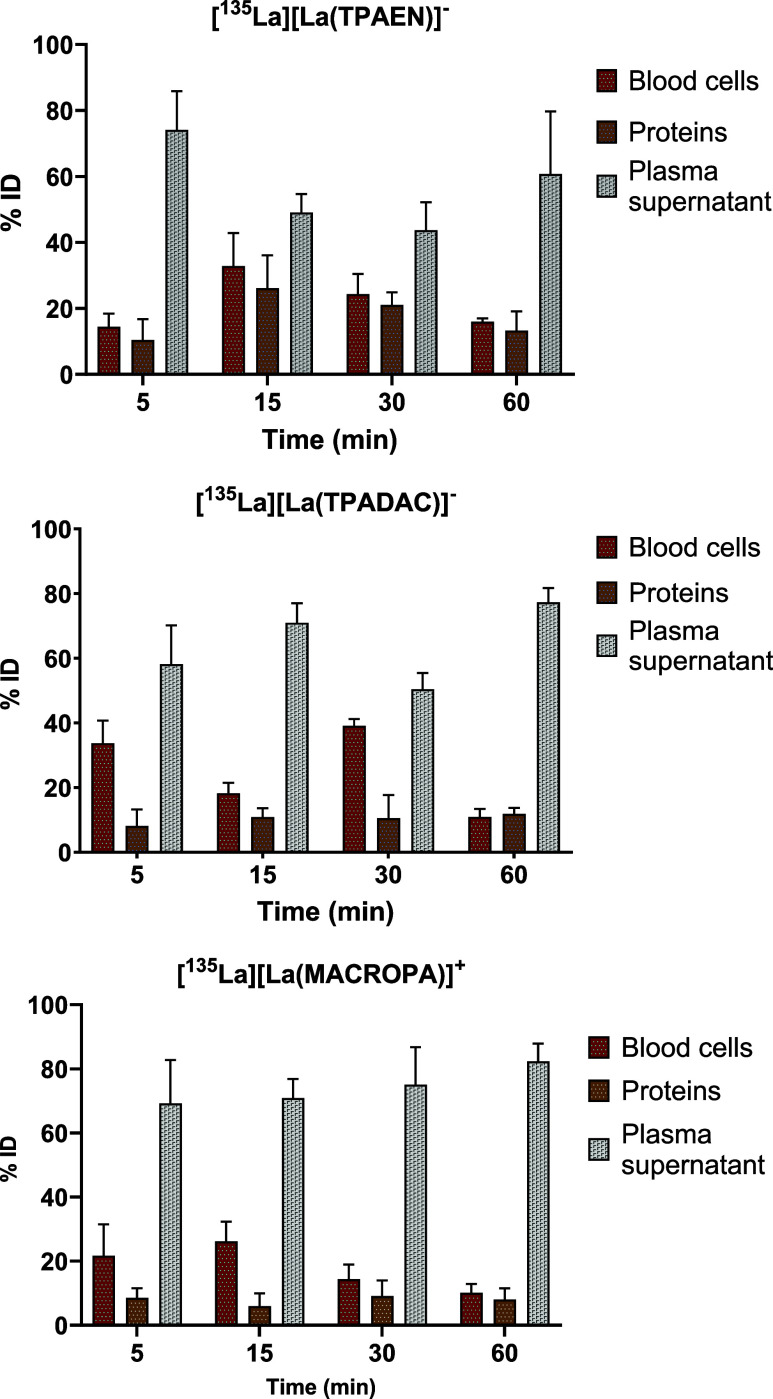
*In vivo* metabolic profiling of [^135^La]­[La­(TPAEN)]^−^ (top), [^135^La]­[La­(TPADAC)]^−^ (center) and [^135^La]­[La­(MACROPA)]^+^, and blood compartments (blood cells, plasma proteins, and supernatant).

Radio-TLC analysis of the supernatant revealed that [^135^La]­[La­(TPAEN)]^−^ remained intact and stable even after 60 min *in vivo*, equally to [^135^La]­[La­(MACROPA)]^+^. In contrast, [^135^La]­[La­(TPADAC)]^−^ showed rapid degradation, with around 80% of the complex breaking down within the first 5 min and approximately 95% degraded by 60 min postadministration (Figure 11). These results indicate that while both complexes circulate in the plasma, [^135^La]­[La­(TPAEN)]^−^ demonstrates significantly greater *in vivo* stability than [^135^La]­[La­(TPADAC)]^−^.

**Figure 11:**
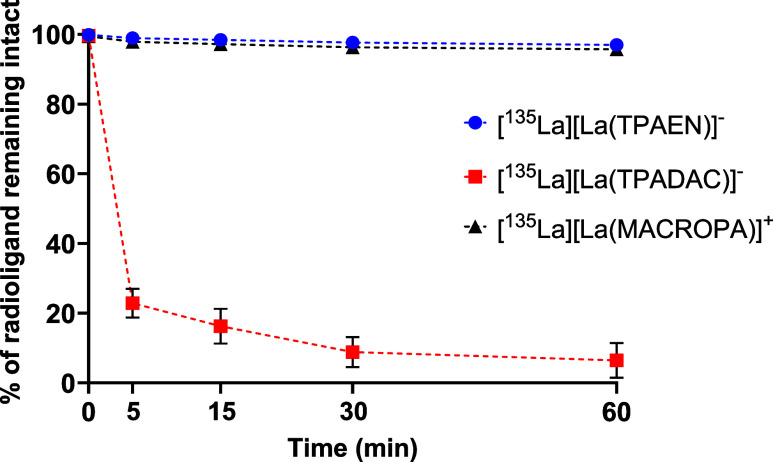
Radio-TLC analysis of the supernatant after *in vivo* administration of [^135^La]­[La­(TPAEN)]^−^, [^135^La]­[La­(TPADAC)]^−^ and [^135^La]­[La­(MACROPA)]^+^.

The higher *in vivo* stability of [^135^La]­[La­(TPAEN)]^−^ compared with [^135^La]­[La­(TPADAC)]^−^ is somewhat surprising considering previous studies, in which chelator rigidification using cyclohexyl spacers was found to improve complex stability of [^177^Lu]­Lu^3+^, [^90^Y]­Y^3+^ and [^67^Ga]­Ga^3+^ complexes.^34,70^ Furthermore, the dissociation kinetics studies presented above indicate a slower dissociation of [^135^La]­[La­(TPADAC)]^−^ compared with [^135^La]­[La­(TPAEN)]^−^ following the proton- and copper-assisted dissociation pathways. These results highlight the difficulties in predicting the stability of radio-complexes *in vivo* using kinetic data generated *in vitro*. The complexity of the environment in which the complex is dissolved *in vivo* makes predictions particularly difficult, as competition of cations, anions and proteins may facilitate complex dissociation.^71–73^ The higher thermodynamic stability of the complex with TPAEN^4–^ compared with the cyclohexyl analogue (see above) may be a factor contributing to an increased *in vivo* stability.^74^ The Log P values were determined to be −1.78 ± 0.09 and −1.26 ± 0.06 for [^135^La]­[La­(TPAEN)]^−^ and [^135^La]­[La­(TPADAC)]^−^, respectively. Thus, even when the latter is slightly more lipophilic, the negative value of the logarithm of the partition coefficient indicates that both complexes have a higher affinity for the aqueous phase compared to the organic (lipid) phase. As a result, no major differences in clearance kinetics are expected for the two complexes.^75^

## Conclusions

The H_4_TPAEN and H_4_TPADAC ligands form ten-coordinate complexes both in the solid state and in solution endowed with high thermodynamic stability. The structural and thermodynamic data indicate that H_4_TPAEN provides a more efficient binding of the large La^3+^ ion than the cyclohexyl analogue H_4_TPADAC, in contrast to the trends observed for complexes with smaller metal ions.

The acyclic ligands H_4_TPAEN and H_4_TPADAC were efficiently radiolabeled with ^135^La, achieving high radiochemical yields within short reaction times. Stability studies, both *in vitro* and *in vivo*, showed that [^135^La]­[La­(TPAEN)]^−^ displayed significantly higher stability compared to [^135^La]­[La­(TPADAC)]^−^. Among the two, H_4_TPAEN stood out as the most promising ligand, forming a stable complex with radiolanthanum under mild conditions and maintaining its integrity in biological environments. Our results indicate that the stability of [^135^La]­[La­(TPAEN)]^−^ *in vivo* is comparable to that of the [^135^La]­[La­(MACROPA)]^+^, as no signs of significant dissociation were observed for any of the complexes. Both TPAEN^4–^ and the leading chelator for large metal ions MACROPA^2–^, can be radiolabeled with [^135^La]­La^3+^ under very mild conditions, in contrast to the ubiquitous DOTA^4–^,^21^ which displays slow radiolabeling kinetics at 37 °C. Radiolabeling under mild conditions is critical for the use of radio-conjugates containing temperature sensitive targeting units like antibodies. Thus, the results reported here support H_4_TPAEN as a strong candidate for further development. Given its performance, H_4_TPAEN should be explored as a bifunctional chelating agent for conjugation to biomolecules such as peptides and proteins, potentially expanding its use in targeted radiopharmaceutical applications. Studies involving other large f-block radiometals (i.e., ^225^Ac) are currently underway.

## Experimental Section

### General Information

Solvents and reagents were purchased from commercial sources and were directly used without further purification. Medium performance liquid chromatography (MPLC) was performed in a Puriflash XS 420 InterChim Chromatographer equipped with a UV-DAD detector in reverse phase, using a 20 g BGB Aquarius C18AQ reversed-phase column (100 Å, spherical, 15 μm) with a 0.1% TFA aqueous solution (phase A) and CH_3_CN with 20% of phase A (phase B) as the mobile phases (flow rate 15 mL/min). Preparative high performance liquid chromatography (HPLC) was performed using an Agilent 1260 Infinity II instrument equipped with an UV variable wavelength detector, in manual injection and collection mode, using an Agilent InfinityLab ZORBAX 5 Eclipse Plus C18 (5 μm, 21.2 × 250 mm) and 10 mM ammonium acetate aqueous solution (phase A) and CH_3_CN with 10% of phase A (phase B) as the mobile phases, operating at a flow rate of 20 mL/min. Analytical HPLC analysis of the pure ligands were performed in a Thermo Scientific UltiMate 3000 connected to a photodiode array detector using a Polar-C18 Luna Omega analytical column from Phenomenex (100 Å, 3 μm, 150 × 2.1 mm), with H_2_O and CH_3_CN + 0.04% TFA as the mobile phases (flow rate of 0.3 mL/min). All compounds are >97% pure by HPLC analysis. High-resolution electrospray-ionization time-of-flight ESI-TOF mass spectra were recorded in positive mode using a LTQ-Orbitrap Discovery Mass Spectrometer coupled to a Thermo Accela HPLC. Aqueous solutions were lyophilized using a Biobase BK-FD10 Series apparatus. ^1^H and ^13^C NMR spectra of the ligands and their precursors were recorded on a Bruker AVANCE 500 or 400 MHz. Elemental analyses of the ligands were performed using a ThermoQuest Flash EA 1112 elemental analyzer.

#### Synthesis of *N,N,N′N′*-tetrakis­[(6-carboxypyridin-2-yl)­methyl]-ethylenediamine (H_4_TPAEN)

To a solution of 6-chloromethylpyridine-2-carboxylic acid ethyl ester (892.8 mg, 4.46 mmol) in acetonitrile (10 mL), K_2_CO_3_ (609.6 mg, 4.40 mmol) and ethylenediamine (67 μL, 1 mmol) were consecutively added under argon atmosphere. The reaction mixture was refluxed for 48 h, followed by the filtration and evaporation of the solvent to give an orange oil. The crude product was then refluxed for 4h in a 6 M HCl aqueous solution (30 mL). After evaporation of the solvent, the resulting oil was dissolved in MeOH (2 mL), to which acetone (80 mL) was added to precipitate the product as an off-white solid. Upon filtration, washing with acetone and then drying under vacuum, the pure hydrochloric salt of the ligand was obtained with a global yield of 57% (386.4 mg). ^1^H NMR (500 MHz, D_2_O, pD 2.8, 298 K) δ (ppm): 8.05–7.95 (m, 8H), 7.64 (d, *J* = 7.2 Hz, 4H), 4.54 (s, 8H), 3.82 (s, 4H). ^13^C NMR (126 MHz, D_2_O, pD 2.8, 298 K) δ (ppm): 166.18, 151.38, 146.43, 141.21, 128.17, 125.37, 58.18, 50.90. Elemental analysis calcd (%) for C_30_H_28_N_6_O_8_·HCl·1.65H_2_O: C 54.04, H 4.88, N 12.60; found C 54.06, H 4.77, N 12.28. Experimental MS (ESI^+^, H_2_O): *m*/*z* 601.2046, 623.1864; calculated for [C_30_H_29_N_6_O_8_]^+^ 601.2042, calculated for [C_30_H_28_N_6_NaO_8_]^+^ 623.1861.

#### Synthesis of (1*R*,2*R*)-*N,N,N′,N′*-tetrakis­[6-carboxypyridin-2-yl)­methyl]­diaminocyclohexane (H_4_TPADAC)

To a solution of 6-chloromethylpyridine-2-carboxylic acid ethyl ester (898.4 mg, 4.5 mmol) in acetonitrile (10 mL), K_2_CO_3_ (615 mg, 4.45 mmol) and enantiomerically pure (1*R*,2*R*)-cyclohexane-1,2-diamine (114.2 mg, 1 mmol) were consecutively added under argon atmosphere. The reaction mixture was refluxed for 72 h, followed by the filtration and evaporation of the solvent to give an orange oil. The crude product was refluxed for 4 h in a 6 M HCl aqueous solution (30 mL), after which the solvent was evaporated to give a brown oil. This oil was dissolved in water (2 mL) and was purified by reverse phase MPLC (Table S5, Supporting Information). The desired compound eluted at 12% CH_3_CN (15% phase B, retention time 25.6 min). The fraction of interest was lyophilized to obtain an off-white solid (211.6 mg, 32% yield). ^1^H NMR (500 MHz, D_2_O, pD 2.2, 298 K) δ (ppm): 8.35–7.14 (m, 12H), 4.39 (m, 6H), 4.14 (d, *J* = 8.8 Hz, 2H), 3.96 (s, 2H), 2.51 (d, *J* = 11.7 Hz, 2H), 2.03 (d, *J* = 9.4 Hz, 2H), 1.77 (q, *J* = 11.8, 8.9 Hz, 2H), 1.53 (p, *J* = 11.8, 10.0 Hz, 2H).^13^C NMR (126 MHz, D_2_O, pD 2.2, 298 K) δ (ppm): 166.04, 153.80, 152.29, 146.52, 142.66, 140.09, 129.21, 126.79, 125.29, 62.28, 56.14, 52.38, 23.88, 23.55. Elemental analysis calcd (%) for C_34_H_34_N_6_O_8_·2C_2_F_3_O_2_H·1.5H_2_O: C 52.00, H 4.48, N 9.57; found C 51.95, H 4.43, N 9.62. Experimental MS (ESI^+^, H_2_O): *m*/*z* 655.2576; calculated for [C_34_H_35_N_6_O_8_]^+^ 655.2511.

Both chelators were further purified by preparative HPLC before performing the radiolabeling experiments to ensure high purity (Table S6, Figures S34–S35, Supporting Information).

### General Procedure for the Synthesis of the Lanthanum Complexes

The La^3+^ complexes were prepared *in situ* by adding 1.1 equiv of LaCl_3_·6H_2_O to a solution of the corresponding chelator in deuterated water (0.4 mL, pD 6.6) and stirring for 5 min. Since the complexation process lowers the pD of the mixture, a diluted solution of NaOD was used to adjust de pD above pD 7. The formation of the complexes was monitored using NMR.

#### Synthesis of [La­(TPAEN)]^−^

Following the general procedure the desired compound was obtained. ^1^H NMR (500 MHz, D_2_O, pD 7.4, 298 K) δ (ppm): 7.98 (t, *J* = 7.8 Hz, 2H), 7.95–7.86 (m, 4H), 7.76 (d, *J* = 7.6 Hz, 2H), 7.47 (d, *J* = 7.8 Hz, 2H), 7.41 (d, *J* = 7.7 Hz, 2H), 4.02 (d, *J* = 17.1 Hz, 2H), 3.91 (d, *J* = 15.5 Hz, 2H), 3.81 (d, *J* = 17.3 Hz, 2H), 3.25 (d, *J* = 15.7 Hz, 2H), 2.84 (d, *J* = 10.4 Hz, 2H), 2.67 (d, *J* = 10.3 Hz, 2H). ^13^C NMR (126 MHz, D_2_O, pD 7.4, 298 K) δ (ppm): 173.49, 171.51, 160.35, 155.31, 151.74, 150.94, 141.15, 140.39, 126.10, 124.65, 123.70, 123.57, 63.37, 62.43, 59.36. Experimental MS (ESI^+^, H_2_O): *m*/*z* 737.0875, 369.0476, 759.0606; calculated for [C_30_H_26_LaN_6_O_8_]^+^ 737.0871, calculated for [C_30_H_27_LaN_6_O_8_]^2+^ 369.0472, calculated for [C_30_H_25_LaN_6_NaO_8_]^+^ 759.0690.

#### Synthesis of [La­(TPADAC)]^−^

Following the general procedure the desired compound was obtained. ^1^H NMR (500 MHz, D_2_O, pD 7.8, 298 K) δ (ppm): 8.13–7.87 (m, 6H), 7.77 (s, 2H), 7.46 (d, *J* = 17.5 Hz, 4H), 4.15 (d, *J* = 15.0 Hz, 2H), 4.07 (d, *J* = 13.9 Hz, 2H), 3.59 (d, *J* = 13.9 Hz, 2H), 3.13 (d, *J* = 15.1 Hz, 2H), 2.44 (s, 2H), 2.17–2.03 (m, 2H), 1.83 (s, 2H), 1.64 (s, 2H), 1.21–1.00 (m, 2H).^13^C NMR (126 MHz, D_2_O, pD 7.8, 298 K) δ (ppm): 173.28, 171.21, 159.39, 155.22, 151.82, 150.68, 141.69, 140.58, 126.51, 124.99, 123.75, 123.39, 66.05, 59.91, 53.68, 24.30, 24.00. Experimental MS (ESI^–^, H_2_O): *m*/*z* 789.1205; calculated for [C_34_H_30_LaN_6_O_8_]^−^ 789.1194.

### Equilibrium and Kinetic Measurements

The chemicals used in the studies were of the highest analytical grade. The LaCl_3_ solution was prepared by dissolving La_2_O_3_ (Merck, 99.99%) in 6.0 M HCl and evaporating the excess of acid. The concentrations of the metal chloride solutions were determined by complexometric titration with the use of a standardized Na_2_H_2_EDTA solution and xylenol orange (LnCl_3_) and murexid (CuCl_2_) indicators.

The pH-potentiometric titrations were carried out with a Methrohm 785 DMP titrator using a Metrohm-6.0233.100 combined electrode. The titrated solutions (5.000 mL) were thermostated at 25 °C and stirred and kept under inert gas atmosphere (N_2_) to avoid the effect of CO_2_. The pH-calibration of the electrode was performed using KH-phthalate (pH = 4.005) and borax (pH = 9.177) buffers. The concentrations of the ligand stock solutions were determined by pH-potentiometric titration. The protonation constants of the TPAEN^4–^ (in the pH range of 2.19–12.00 a total of 213 data pairs by titrating a 2.72 mM a ligand solution) and TPADAC^4–^ (in the pH range of 1.88–11.85 a total of 208 data pairs by titrating a 2.32 mM ligand solution), the stability and protonation constants of the complexes formed with La^3+^ and the formation constants of their dinuclear complexes were determined by pH-potentiometric titration. The metal-to-ligand concentration ratios were 1:1 and 2:1. For the 1:1 ratio a total of 240 data pairs were recorded for a H_4_TPAEN concentration of 2.29 mM in the pH range of 1.77–11.86, while a total of 222 data pairs were collected in the pH range 1.72–11.85 for a 2.72 mM solution of H_4_TPADAC. For the 2:1 ratio we collected 259 data pairs for a 2.31 mM solution of H_4_TPAEN in the pH range of 1.73–11.82, and 259 data pairs for a 2.71 mM concentration of H_4_TPADAC in the pH range of 1.70–11.85. The calculation of [H^+^] from the measured pH values was performed with the use of the method proposed by Irving et al.^76^ by titrating a 0.01 M HCl solution (*I* = 0.15 M NaCl) with a standardized NaOH solution. The differences between the measured and calculated pH values were used to obtain the [H^+^] concentrations from the pH-data obtained in the titrations. The ionic product of water was determined from the same experiment in the pH range 11.25–11.85. The protonation and stability constants were calculated from the titration data with the PSEQUAD program.^77^ The quality of the fits was assessed with the fitting parameter (FP) calculated by PSEQUAD, which is defined as

2 $$\mathrm{FP}=\sqrt{\frac{\sum_{i=1}^{n}{\left(V_{i,\mathrm{calc}}-V_{i,\mathrm{meas}}\right)}^{2}}{n-k}}$$

In eq 2, *V*_*i*,calc_ and *V*_*i*,meas_ are the calculated and experimental volumes of base added at a given pH, *n* is the number of data pairs and k is the number of fitted parameters.

The rates of the metal exchange reactions of the [La­(TPAEN)]^−^ and [La­(TPADAC)]^−^ complexes with Cu­(II) were studied by using UV–vis spectrophotometry following the formation of corresponding Cu­(II) complexes. The metal exchange reactions of [La­(TPAEN)]^−^ and [La­(TPADAC)]^−^ were examined by conventional spectrophotometric methods tracking the changes in absorption at 305 nm within the pH range of 1.84 to 4.94 (for [La­(TPAEN)]^−^). The concentration of the complexes was 0.252 mM, while the Cu­(II) ion was applied in high excess (12 to 42 times) to ensure pseudo-first-order conditions. The temperature was maintained at 25 °C, and the ionic strength of the solutions was kept constant at 0.15 M NaCl. Dimethylpiperazine (DMP, 50 mM) buffer was used to maintain the pH constant (log *K*_2_^H^ = 4.18) in the pH range of 3.40 to 4.89 (for [La­(TPADAC)]^−^). At lower pH, the data were supplemented by following the reactions in the range pH of 2.57 to 3.23 using chloroacetic acid (log *K*_2_^H^ = 2.87) at the same concentration. The pseudo-first-order rate constants (*k*_obs_) were calculated by fitting the absorbance vs time data to eq 3:

3 $$A_{t}=\left(A_{0}-A_{e}\right)e^{-k_{\mathrm{obs}}t}+A_{e}$$

where *A*_*t*_, *A*_*0*_ and *A*_*e*_ are the absorbances recorded at time *t*, at the start of the reaction and upon reaching equilibrium, respectively. The fittings were performed with the computer program Micromath Scientist, version 2.0 (Salt Lake City, UT) by using a standard least-squares procedure.

### Crystal Structure Determinations

Single crystals of [La­(HTPAEN)] were obtained by adding LaCl_3_·6H_2_O (12.9 mg, 0.037 mmol) to a saturated solution of H_4_TPAEN (20 mg, 0.033 mmol) with triethylamine (9.28 μL, 0.066 mmol) in methanol (0.4 mL). Slow evaporation of the solvent afforded colorless prisms that were analyzed by X-ray diffraction. Single crystals of [La­(TPADAC)]^−^ were grown by slow evaporation of a concentrated aqueous solution of the complex, yielding colorless prisms that were analyzed by X-ray diffraction.

Crystallographic data and the structure refinement parameters corresponding to [H_5_TPAEN]­Cl·3H_2_O, [La­(HTPAEN)]·12H_2_O and [LaCl­(H_2_O)_3_]­[La­(TPADAC)]­Cl·3H_2_O are given in Table S7, Supporting Information. Measurements were performed on a Bruker D8 Venture diffractometer with a Photon 100 CMOS detector at 100 K and Mo–Kα radiation (λ = 0.71073 Å) generated by an Incoatec high brillance microfocus source equipped with Incoatec Helios multilayer optics. The software or APEX4 ^78^ was used for collecting frames of data, indexing reflections, and the determination of lattice parameters, SAINT^79^ for integration of intensity of reflections, and SADABS^80^ for scaling and empirical absorption correction. The SHELXT^81^ program was used for solving the structure by dual-space methods. All non-hydrogen atoms were refined with anisotropic thermal parameters by full-matrix least-squares calculations on F^2^ using the program SHELXL-2014.^82^ Hydrogen atoms of the compound were inserted at calculated positions and constrained with isotropic thermal parameters. For 32109002, highly disordered solvent molecules were removed using the Solvent Mask routine from OLEX2.^83^ CCDC 2450303, 2450304, and 2449869 contain the supplementary crystallographic data, which can be obtained free of charge from the Cambridge Crystallographic Data Centre via www.ccdc.ac.uk/data_request/cif.

### Radiolabeling Studies with ^135^La

H_4_TPAEN and H_4_TPADAC were dissolved in ultrapure deionized water and then diluted to 10^–2^ M stock solutions; subsequent dilutions from 10^–3^ to 10^–6^ M of both ligands were prepared. ^133/135^La was produced following the procedure previously reported using ^nat^BaCO_3_ target,^2^ allowing the decay of coproduced ^133^La to leave highly pure ^135^La available. To assess the optimal conditions for radiolabeling both H_4_TPAEN and H_4_TPADAC, ^135^LaCl_3_ aliquots (30–40 MBq, ∼pH 1.5) were adjusted from pH 1.5 to 10 using HCl, NaOH or pH 9 1 M NaOAc buffer; different H_4_TPAEN/H_4_TPADAC masses (0.01–10 μg) and reaction times (1–60 min) were tested at room temperature. Percentage of radiochemical conversion was evaluated by reverse phase radio-TLC using Methanol/10% Ammonium acetate (3/1) as mobile phase and TLC Aluminum sheets Nano silica gel 60 RP-18W as stationary phase. All radiolabeling experiments were conducted in triplicate.

### Stability Experiments

*In vitro* stability studies were performed with both [^135^La]­[La­(TPAEN)]^−^ and [^135^La]­[La­(TPADAC)]^−^ at 37 °C with incubation times 0, 1, 4, 24, and 48 h: (a) in whole mouse blood; (b) buffers 0.1 M glycine-HCl pH 2.2, reaction media (pH 4–5), 1 × PBS pH 6.8, Krebs buffer pH 7.4, 1 M NaOAc pH 9.4 and (c) in human serum, and analyzed by radio-TLC.

### Challenge Experiments

H_4_DOTA, H_3_NOTA, H_4_EDTA, H_4_TETA, DiAmSar and H_2_MACROPA (10× molar excess of each compared to initial “cold” 0.1 μg of H_4_TPAEN/H_4_TPADAC) were incubated with [^135^La]­[La­(TPAEN)]^−^ and [^135^La]­[La­(TPADAC)]^−^ at 37 °C, and the percentage of [^135^La]­[La­(TPAEN)]^−^ and ^135^La–H_4_T remaining intact was assessed by radio-TLC at 5, 15, 30, 60, and 120 min of incubation. To assess the R_*f*_ differences between [^135^La]­[La­(TPAEN)]^−^/ [^135^La]­[La­(TPADAC)]^−^ and radio-complexes [^135^La]­[La­(DOTA)]^−^, [^135^La]­[La­(NOTA)], [^135^La]­[La­(EDTA)]^−^, [^135^La]­[La­(TETA)]^−^, [^135^La]­[La­(DiAmSar)]^3+^ and [^135^La]­[La­(MACROPA)]^+^, a labeling experiment was carried out to radiolabel H_4_DOTA, H_3_NOTA, H_4_EDTA, H_4_TETA, DiAmSar and H_2_MACROPA with ^135^La. In short, H_4_DOTA, H_3_NOTA, H_4_EDTA, H_4_TETA, DiAmSar and H_2_MACROPA (10 μg) were added to a 4.5 pH-previously buffered ^135^La solution (50 μL, ∼ 40 MBq) and incubated at 37 and/or 70 °C. Percentage of radiochemical conversion was evaluated by radio-TLC as stated before at 5, 15, 30, and 60 min of reaction. All experiments were carried out in triplicate.

### Partition Coefficients

[^133/135^La]­[La­(TPAEN)]^−^ and [^133/135^La]­[La­(TPADAC)]^−^ (90–100 MBq) were dissolved in 500 μL of distilled H_2_O in 1.5 mL Eppendorf tubes, and 500 μL of octan-1-ol (Sigma-Aldrich (St. Louis, MO)) was added. The tubes were capped and shaken vigorously for 2–3 min and subsequently centrifuged for 30 min (5000 rpm; Rotina 35R, Hettich Zentrifugen, Tuttlingen, Germany). Aliquots (300 μL) of the resulting organic top layer (representing [^133/135^La]­[La­(TPAEN)]^−^ or [^133/135^La]­[La­(TPADAC)]^−^ dissolved in octanol), and bottom layer ([^133/135^La]­[La­(TPAEN)]^−^ or [^133/135^La]­[La­(TPADAC)]^−^ dissolved in H_2_O) were taken and the radioactivity in the samples was measured using a GEM35P4–70-SMP high-purity germanium detector (ORTEC, Oak Ridge, TN) with ORTEC GammaVision software. Five octanol – H_2_O mixtures were analyzed (n = 5), and the octanol – water partition coefficient (*P*) was calculated by dividing the octanol-containing radioactivity by the water-containing radioactivity:

4 $$P=\frac{A_{\mathrm{org}}}{A_{\mathrm{aq}}}$$

Here *A*_org_ is the activity (Bq) in the organic phase (n-octanol) and *A*_aq_ is the activity in the aqueous phase.

### *In Vivo* Studies

All animal experiments followed the guidelines of the Canadian Council on Animal Care (CCAC) and were approved by the local animal care committee of the Cross Cancer Institute (animal protocol # AC22264). Control nu/nu Nude mice were used for analyzing *in vivo* metabolic stability of both [^135^La]­[La­(TPAEN)]^−^ and [^135^La]­[La­(TPADAC)]^−^. The animals were anesthetized through inhalation of isoflurane in 100% oxygen (gas flow, 1 L/min). 100–120 MBq of [^135^La]­[La­(TPAEN)]^−^ or [^135^La]­[La­(TPADAC)]^−^ in ∼180 μL saline was injected into the tail vein through a needle catheter. At 5-, 15-, 30- and 60 min postinjection blood samples of ∼20 μL were collected. Blood cells were separated by immediate centrifugation (5 min at 13,000 rpm). Subsequently, proteins were precipitated by adding 200 μL of methanol to the supernatant following a second centrifugation step (5 min at 13,000 rpm). The radioactivity present in each blood fraction was measured using a HIDEX automated γ counter (Hidex Oy, Turku, Finland). For the evaluation of the *in vivo* metabolic stability of both radiolanthanum-labeled complexes, the supernatant plasma fractions collected at 5, 15, 30, and 60 min were analyzed by reverse phase radio-TLC as stated before.

## Supplementary Material





## Notes

NMR spectra and data, potentiometric curves, speciation diagrams, mass spectra, HPLC conditions and traces, additional radiolabeling data and crystallographic data (PDF)
Compound names, molecular formula strings (CSV)

## Notes

This paper is dedicated to Prof. Ernő Brücher on the occasion of his 90th birthday.

## Abreviations Used

H_4_CDTA: 2,2′,2″,2‴-((*trans*-cyclohexane-1,2-diyl)­bis­(azanetriyl))­tetraacetic acid
H_4_ *CHX*OCTAPA: 6,6′-((((1*R*,2*S*)-cyclohexane-1,2-diyl)­bis­((carboxymethyl)­azanediyl))­bis­(methylene))­dipicolinic acid
CR: crystal radius
DAD: diode array detector
DiAmSar: 3,6,10,13,16,19-hexaazabicyclo­[6.6.6]­icosane-1,8-diamine
DMP: dimethylpiperazine
H_3_DO3A: 1,4,7,10-tetrazacyclononane-1,4,7-triacetic acid
H_4_DO3Apic: 2,2′,2″-(10-((6-carboxypyridin-2-yl)­methyl)-1,4,7,10-tetraazacyclododecane-1,4,7-triyl)­triacetic acid
H_4_DOTA: 1,4,7,10-tetraazacyclododecane-1,4,7,10-tetraacetic acid
H_5_DTPA: diethylenetriamine-*N*,*N*,*N*′,*N*″,*N*″-pentaacetic acid
H_4_EDTA: ethylenediamine-*N*,*N*,*N*′,*N*′-tetraacetic acid
H_2_MACROPA: *N*,*N*’-bis­[(6-carboxy-2-pyridil)­methyl]-4,13-diaza-18-crown-6
MPLC,: medium performance liquid chromatography
H_3_NOTA: 2,2′,2″-(1,4,7-triazonane-1,4,7-triyl)­triacetic acid
H_4_OCTAPA: 6,6′-((ethane-1,2-diylbis­((carboxymethyl)­azanediyl))­bis­(methylene))­dipicolinic acid
H_4_PYTA: 2,2′,2″,2‴-(3,6,10,13-tetraaza-1,8­(2,6)-dipyridinacyclotetradecaphane-3,6,10,13-tetrayl)­tetraacetic acid
H_4_TETA: 1,4,8,11-tetraazacyclotetradecane-1,4,8,11-tetraacetic acid
H_4_TPADAC: (1*R*,2*R*)-*N,N,N′*,*N*′-tetrakis­[6-carboxypyridin-2-yl)­methyl]­diaminocyclohexane
H_4_TPAEN: *N,N,N′N′*-tetrakis­[(6-carboxypyridin-2-yl)­methyl]-ethylenediamine
