# Supplementary material for: Decadentate Acyclic Chelators for Lanthanum Radiopharmaceuticals
Source: J Med Chem. 2025 Aug 19;68(16):17823–39. doi: 10.1021/acs.jmedchem.5c01558 (PMC12406198; doi:10.1021/acs.jmedchem.5c01558)
Supplement: Supplementary file 1 [file jm5c01558_si_001.pdf]

Supporting Information for:

Decadentate Acyclic Chelators for Lanthanum Radiopharmaceuticals

Antía Freire-García,[a,†] Yasniel Babí Araujo,[b,†] Melinda Wuest,[b] Balázs Szilágyi,[c,d] Enikő Madarasi,[c,d] Laura Valencia,[e] Saray Argibay-Otero,[a,e] Aurora Rodríguez-Rodríguez,[a] David Esteban-Gómez,[a] Gyula Tircsó,*,[c] Frank Wuest,*,[b] and Carlos Platas-Iglesias.*,[a]

[a] Universidade da Coruña, Centro Interdisciplinar de Química e Bioloxía (CICA) and Departamento de Química, Facultade de Ciencias, 15071, A Coruña, Galicia, Spain.

[b] Department of Oncology, University of Alberta, Edmonton, Alberta T6G 1Z2, Canada.

[c] Department of Physical Chemistry, Faculty of Science and Technology, University of Debrecen, H-4032 Debrecen, Hungary.

[d] Doctoral School of Chemistry, University of Debrecen, H-4032 Debrecen, Hungary.

[e]Departamento de Química Inorgánica, Facultad de Ciencias, Universidade de Vigo, As Lagoas, Marcosende, 36310 Pontevedra, Spain.

*E-mail: carlos.platas.iglesias@udc.es
*E-mail: wuest@ualberta.ca
*E-mail: gyula.tircso@science.unideb.hu

Summary:

Scheme S1: Synthesis of the ligands H_4TPAEN and $H_4TPADAC$. S5

Figure S1: pH-potentiometric titration curves of TPAEN ligand in the absence (purple) and in the presence of one (green) and two (blue) equivalents of La^{3+} (25 °C, I = 0.15 M NaCl, c_{Lig}=2.7 mM). Fitting parameters: 8.71×10^{-4} cm^3 (TPAEN) and 5.30216×10^{-3} cm^3 (simultaneous fit of 1:1 and 2:1 La:TPAEN) S5

Figure S2: pH-potentiometric titration curves of the TPADAC ligand in the absence (purple) and in the presence of one (green) and two (blue) equivalents of La^{3+} (25 °C, I = 0.15 M NaCl, c_{Lig}=2.3 mM). Fitting parameters: 8.50×10^{-4} cm^3 (TPADAC) and 2.605×10^{-3} cm^3 (simultaneous fit of 1:1 and 2:1 La:TPADAC) S6

Figure S3: Speciation diagrams of the protonation of $TPAEN^{4-}$ (left) and $TPADAC^{4-}$ (right) calculated for c_{Lig}=10^{-3} M (I = 0.15 M NaCl, 25 ºC). S6

Table S1: Experimental ^1H NMR chemical shifts (ppm) of H_4TPAEN and $H_4TPADAC$ (500 MHz, D_2O, 298K). S7

Table S2: Experimental ^{13}C NMR chemical shifts (ppm) of H_4TPAEN and $H_4TPADAC$ (500 MHz, D_2O, 298K). S7

Figure S4: ^1H NMR spectrum (500 MHz, D_2O, pD 2.8, 298K) of compound H_4TPAEN. S8

Figure S5: ^{13}C NMR spectrum (126 MHz, D_2O, pD 2.8, 298K) of compound H_4TPAEN. S8

Figure S6: DEPT-135 spectrum (126 MHz, D_2O, pD 2.8, 298K) of compound H_4TPAEN. S9

Figure S7: COSY spectrum (500 MHz, D_2O, pD 2.8, 298K) of compound H_4TPAEN. S9

Figure S8: HSQC spectrum (500 MHz, D_2O, pD 2.8, 298K) of compound H_4TPAEN. S10

Figure S9: HMBC spectrum (500 MHz, D_2O, pD 2.8, 298K) of compound H_4TPAEN. S10

Figure S10: ^1H NMR spectrum (500 MHz, D_2O, pD 2.2, 298K) of compound $H_4TPADAC$. The asterisk denotes a signal due to residual acetone. S11

Figure S11: ^{13}C NMR spectrum (126 MHz, D_2O, pD 2.2, 298K) of compound $H_4TPADAC$. The asterisk denotes signals due to residual trifluoroacetic acid. S11

Figure S12: DEPT-135 spectrum (126 MHz, D_2O, pD 2.2, 298K) of compound $H_4TPADAC$. The asterisk denotes signals due to residual trifluoroacetic acid. S12

Figure S13: COSY spectrum (500 MHz, D_2O, pD 2.2, 298K) of compound $H_4TPADAC$. S12

Figure S14: HSQC spectrum (500 MHz, D_2O, pD 2.2, 298K) of compound $H_4TPADAC$. S13

Figure S15: HMBC spectrum (500 MHz, D_2O, pD 2.2, 298K) of compound $H_4TPADAC$. S13

Figure S16: ^1H NMR spectrum (500 MHz, D_2O, 298K) of compound $H_4TPADAC$ at different pD values (indicated in the figure). S14

Figure S17: Variable temperature ^{1}H NMR spectrum (400 MHz, D_2O, pD 2.2) of compound H_4TPADAC. S14

Figure S18: Eyring plot of ln(k_{ex}/T) vs 1/T for the interconversion process studied by NMR for H_4TPADAC. Inset: values obtained from the linear fit used to determine the activation parameters of the interconversion process, following Eyring's equation. ...S15

Figure S19: Views of the X-ray crystal structures of the [La(HTPAEN)] (left) and $[La(TPADAC)]^-$ (right) complexes with ellipsoids plotted at the 50% probability level. S15

Figure S20: View of the X-ray crystal structure of $[LaCl(H_2O)_3][La(TPADAC)]Cl\cdot 3H_2O$ depicting the excess La^{3+} coordinating four oxygen atoms of carboxylate groups of four different $[La(TPADAC)]^-$ entities (ellipsoids plotted at the 50% probability level).....S16

Table S3: Experimental ^{1}H NMR chemical shifts (ppm) of $[La(TPAEN)]^-$ and $[La(TPADAC)]^-$ (500 MHz, D_2O, 298K)...S16

Table S4: Experimental ^{13}C NMR chemical shifts (ppm) of $[La(TPAEN)]^-$ and $[La(TPADAC)]^-$ (500 MHz, D_2O, 298K)...S17

Figure S21: ^{13}C NMR spectrum (126 MHz, D_2O, pD 7.4, 298K) of compound $[La(TPAEN)]^-$...S18

Figure S22: DEPT-135 spectrum (126 MHz, D_2O, pD 7.4, 298K) of compound $[La(TPAEN)]^-$...S18

Figure S23: COSY spectrum (500 MHz, D_2O, pD 7.4, 298K) of compound $[La(TPAEN)]^-$...S19

Figure S24: HSQC spectrum (500 MHz, D_2O, pD 7.4, 298K) of compound $[La(TPAEN)]^-$...S19

Figure S25: HMBC spectrum (500 MHz, D_2O, pD 7.4, 298K) of compound $[La(TPAEN)]^-$...S20

Figure S26: ^{13}C NMR spectrum (126 MHz, D_2O, 298K) of compound $[La(TPADAC)]^-$. S20

Figure S27: DEPT-135 spectrum (126 MHz, D_2O, 298K) of compound $[La(TPADAC)]^-$ S21

Figure S28: COSY spectrum (500 MHz, D_2O, pD 7.8, 298K) of compound $[La(TPADAC)]^-$. S21

Figure S29: HSQC spectrum (500 MHz, D_2O, pD 7.8, 298K) of compound $[La(TPADAC)]^-$. S22

Figure S30: Species distribution curves calculated for the 2:1 La^{3+}:$TPAEN^{4-}$ (top) and La^{3+}:$TPADAC^{4-}$ (bottom) systems ($c_{Lig}=10^{-3}$ M, $c_{La3+}=2\cdot 10^{-3}$ M; I = 0.15 M NaCl, 25 ºC). S23

Figure S31: Radiolabeling tests results of H_4DOTA, H_3NOTA, H_4TETA, H_4EDTA, DiAmSar and H_2MACROPA at 37ºC (top) and/or 70ºC (bottom) and pH 4.5 with ^{135}La. S24

Figure S32: Experimental high resolution mass spectrum (ESI^+) of compound H_4TPAEN..S25

Figure S33: Experimental high resolution mass spectrum (ESI$^+$) of compound H_4TPADAC.......S25

Table S5: MPLC separation method (with mobile phases A= 0.1% TFA aqueous solution and B= CH_3CN + 20% A).......S26

Table S6: HPLC separation method for the purification of both chelators (with mobile phases A= ammonium acetate 10 mM aqueous solution and B= CH_3CN + 10% A)...S26

Figure S34: HPLC analysis (272 nm) of H_4TPAEN, retention time 11.08 min. Inset: separation method with A= H_2O + 0.04% TFA, B= CH_3CN + 0.04% TFA.......S26

Figure S35: HPLC analysis (272 nm) of H_4TPADAC, retention time 11.93 min. Inset: separation method with A= H_2O + 0.04% TFA, B= CH_3CN + 0.04% TFA.......S27

Figure S36: Experimental high resolution mass spectrum (ESI$^+$) of compound [La(TPAEN)]$^-$.......S27

Figure S37: Experimental high resolution mass spectrum (ESI$^-$) of compound [La(TPADAC)]$^-$.......S28

Figure S38: Absorption spectra of the free ligands and their La^{3+} and Cu^{2+} complexes (M:L = 1:1) recorded in H_2O solutions: (a) [$TPAEN^{4-}$] = 0.487 mM, pH = 4.57; (b) [$TPADAC^{4-}$] = 0.517 mM, pH = 4.57. The vertical dashed lines indicate the wavelength used for kinetic experiments (305 nm).......S29

Figure S39: CD spectra recorded from aqueous solutions of the H_4TPADAC ligand (0.170 mM, pH 6.35) and the [La(TPADAC)]$^-$ complex (0.28 mM, pH 4.72).......S30

Table S7: Crystal data and structure refinement [H_5TPAEN]Cl·3H_2O, [La(HTPAEN)]·12H_2O and [LaCl(H_2O)$_3$][La(TPADAC)]Cl·3H_2O.......S30

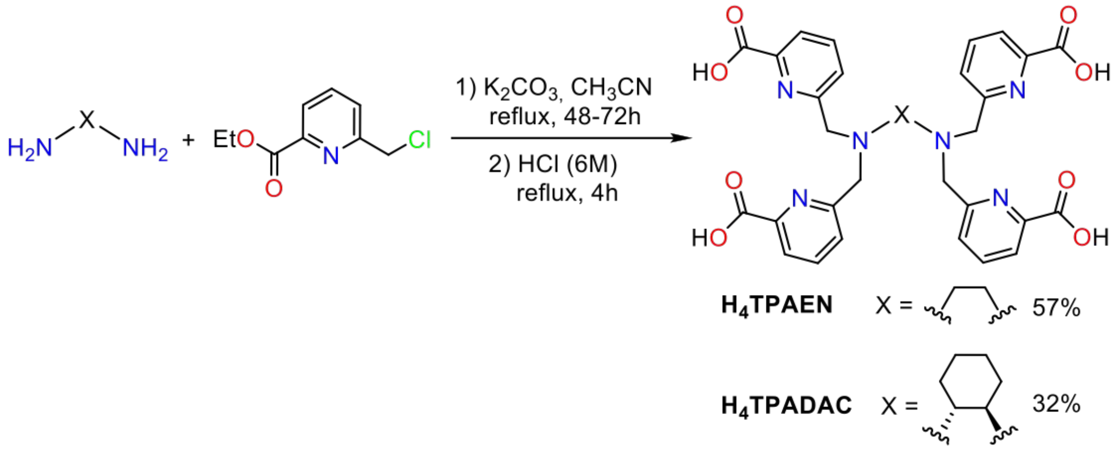

Scheme S1: Synthesis of the ligands H_4TPAEN and H_4TPADAC.

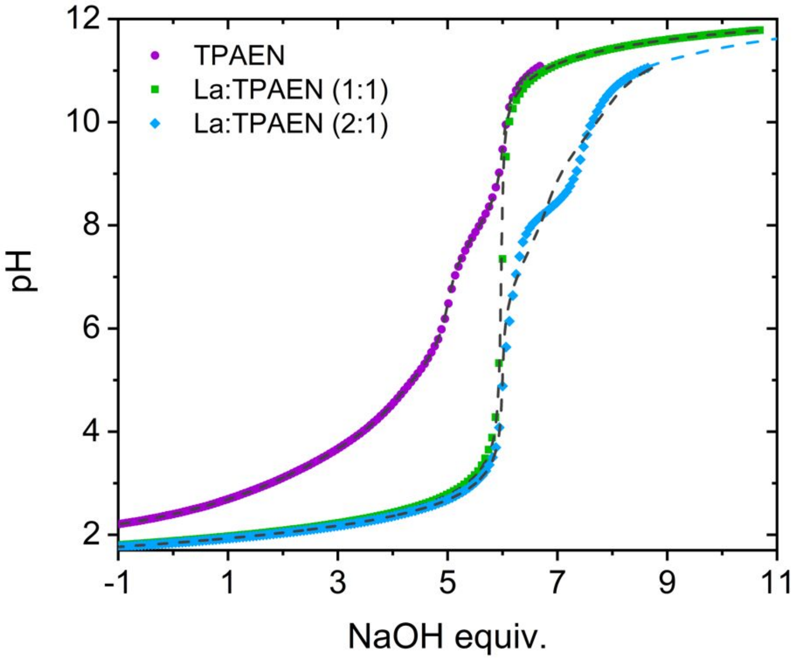

Figure S1: pH-potentiometric titration curves of TPAEN ligand in the absence (purple) and in the presence of one (green) and two (blue) equivalents of La^{3+} (25 °C, I = 0.15 M NaCl, c_{Lig}=2.7 mM). Fitting parameters: 8.71×10^{-4} cm^3 (TPAEN) and 5.30216×10^{-3} cm^3 (simultaneous fit of 1:1 and 2:1 La:TPAEN)

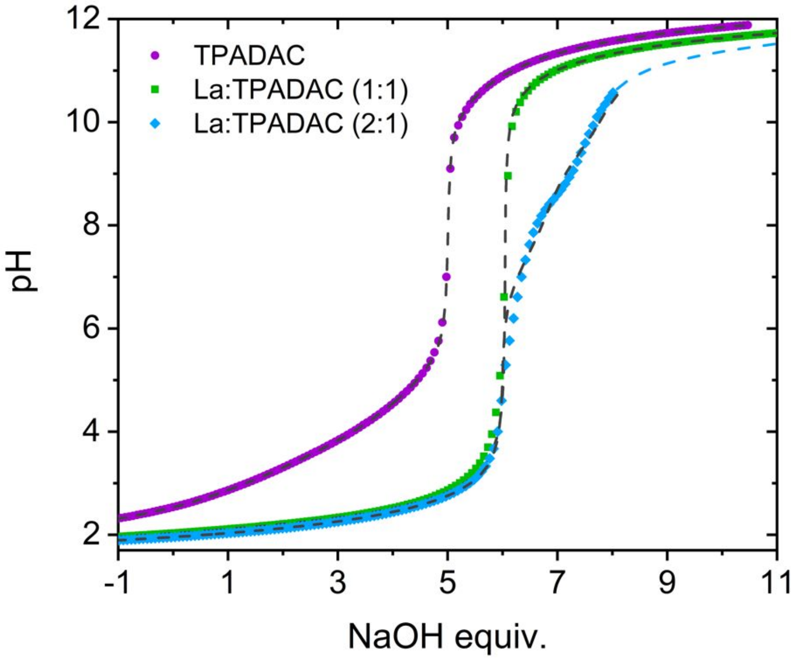

Figure S2: pH-potentiometric titration curves of the TPADAC ligand in the absence (purple) and in the presence of one (green) and two (blue) equivalents of La^{3+} (25 °C, I = 0.15 M NaCl, c_{Lig}=2.3 mM). Fitting parameters: 8.50×10^{-4} cm^3 (TPADAC) and 2.605×10^{-3} cm^3 (simultaneous fit of 1:1 and 2:1 La:TPADAC)

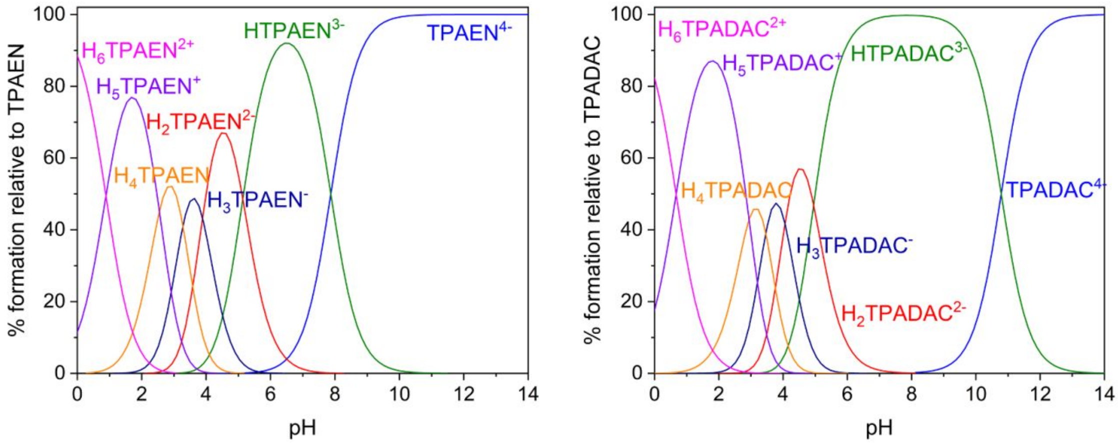

Figure S3: Speciation diagrams of the protonation of $TPAEN^{4-}$ (left) and $TPADAC^{4-}$ (right) calculated for c_{Lig}=10^{-3} M (I = 0.15 M NaCl, 25 ºC).

Table S1: Experimental ^{1}H NMR chemical shifts (ppm) of H_4TPAEN and H_4TPADAC (500 MHz, D_2O, 298K).

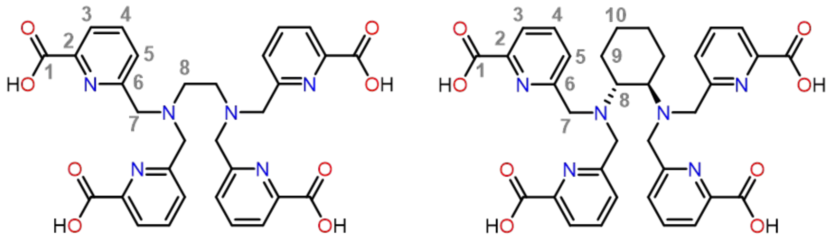

	**H_4TPAEN (pD 2.8)**	**H_4TPADAC (pD 2.2)**
**H3**	7.99 (m, 8H)	7.92 (m, 12H)
**H4**		
**H5**	7.64 (d, 4H), J = 7.2	
**H7 eq**	4.54 (s, 8H)	4.39 (m, 6H)
**H8**	3.82 (s, 4H)	4.14 (d, 2H), J = 8.8
**H7 ax**	-	3.96 (s, 2H)
**H9 eq**	-	2.51 (d, 2H), J = 11.7
**H10 eq**	-	2.03 (d, 2H), J = 9.4
**H9' ax**	-	1.77 (q, 2H), J = 11.8, 8.9
**H10' ax**	-	1.53 (p, 2H), J = 11.8, 10.0

Table S2: Experimental ^{13}C NMR chemical shifts (ppm) of H_4TPAEN and H_4TPADAC (500 MHz, D_2O, 298K).

	**Type**	**H_4TPAEN (pD 2.8)**	**H_4TPADAC (pD 2.2)**
**C1**	C	166.18	166.04
**C6**	C	151.38	153.80
**C6’**	C		152.29
**C2**	C	146.43	146.52
**C4**	CH	141.21	142.66
**C4’**	CH		140.09
**C5**	CH	128.17	129.21
**C5’**	CH		126.79
**C3**	CH	125.37	125.29
**C8**	CH_2/CH	50.90	62.28
**C7**	CH_2	58.18	56.14
**C7’**	CH_2		52.38
**C10**	CH_2	-	23.88
**C9**	CH_2	-	23.55

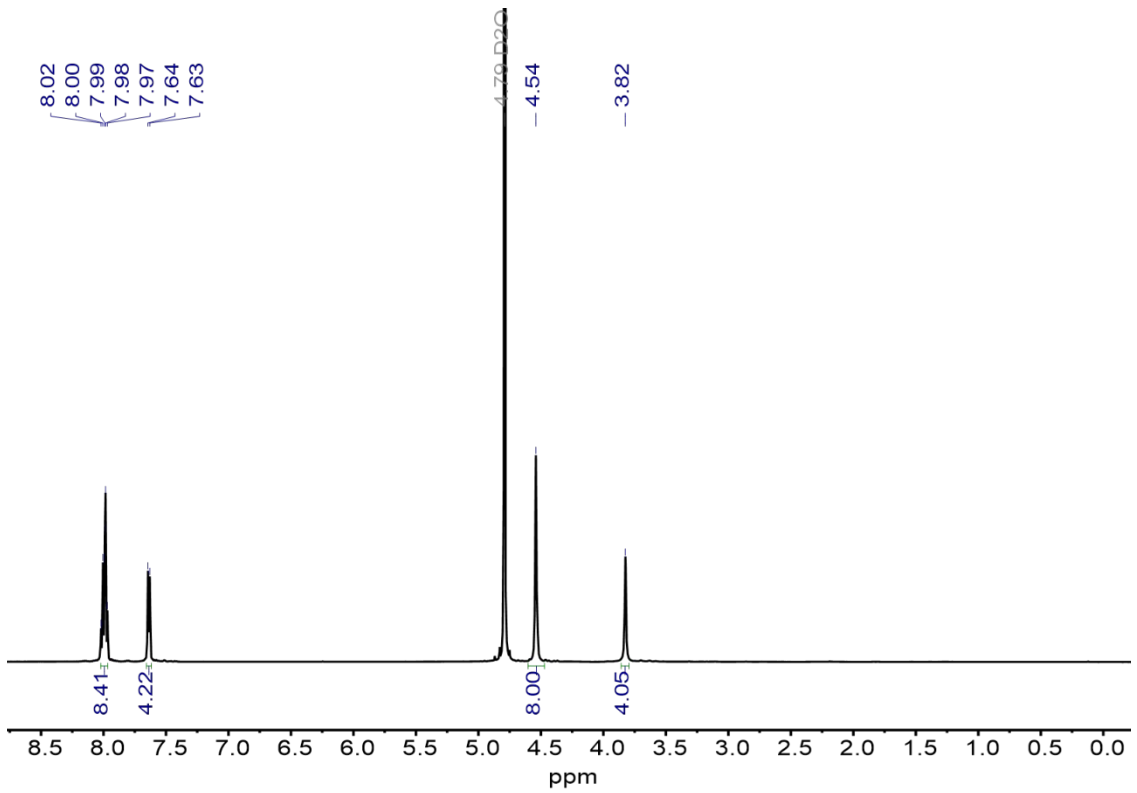

Figure S4: ^{1}H NMR spectrum (500 MHz, D_2O, pD 2.8, 298K) of compound H_4TPAEN.

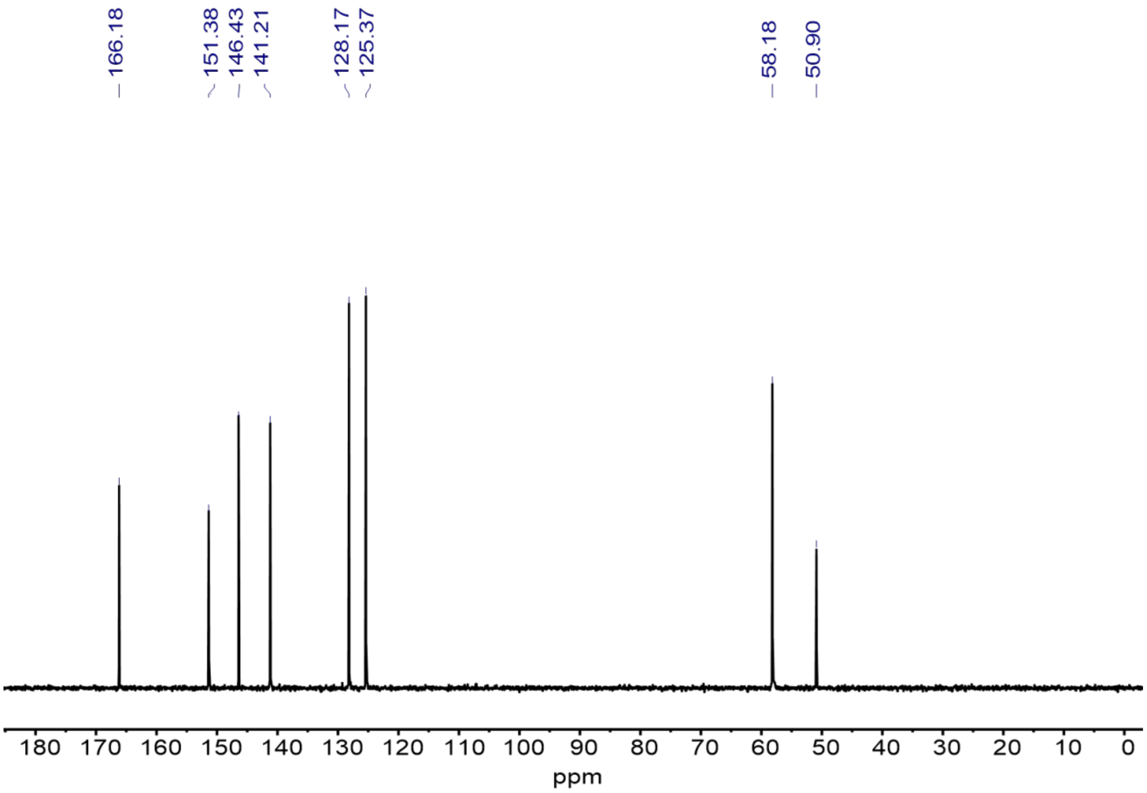

Figure S5: ^{13}C NMR spectrum (126 MHz, D_2O, pD 2.8, 298K) of compound H_4TPAEN.

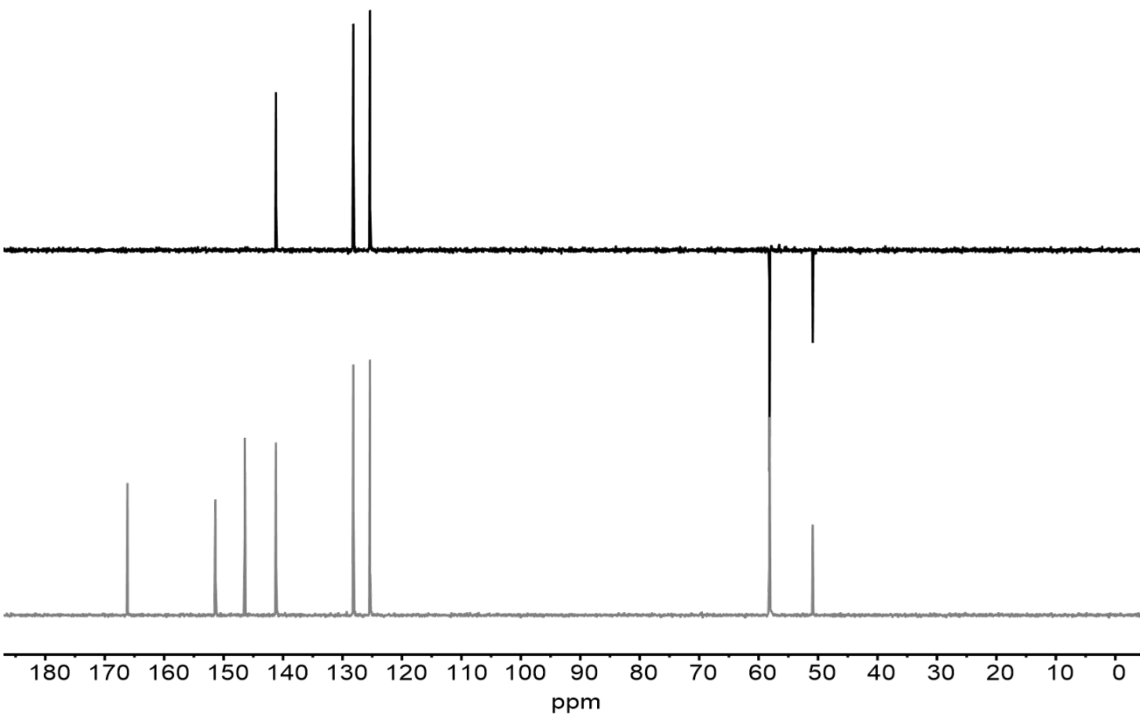

Figure S6: DEPT-135 spectrum (126 MHz, D_2O, pD 2.8, 298K) of compound H_4TPAEN.

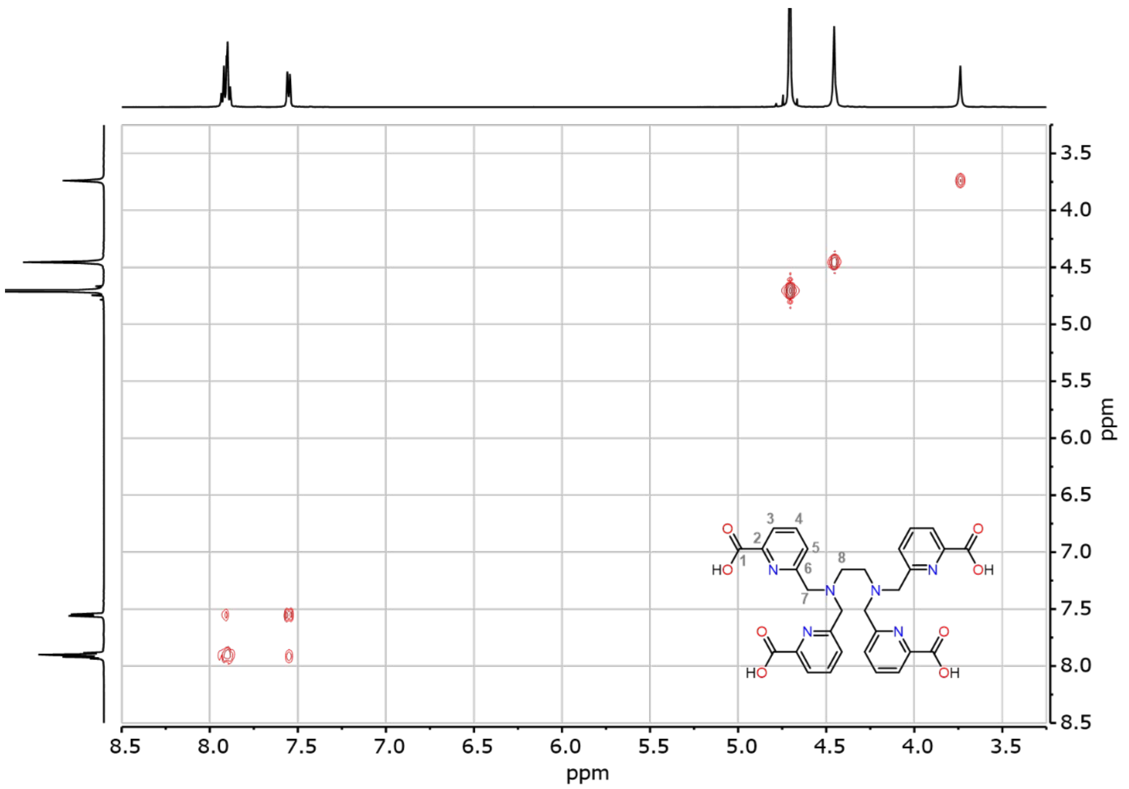

Figure S7: COSY spectrum (500 MHz, D_2O, pD 2.8, 298K) of compound H_4TPAEN.

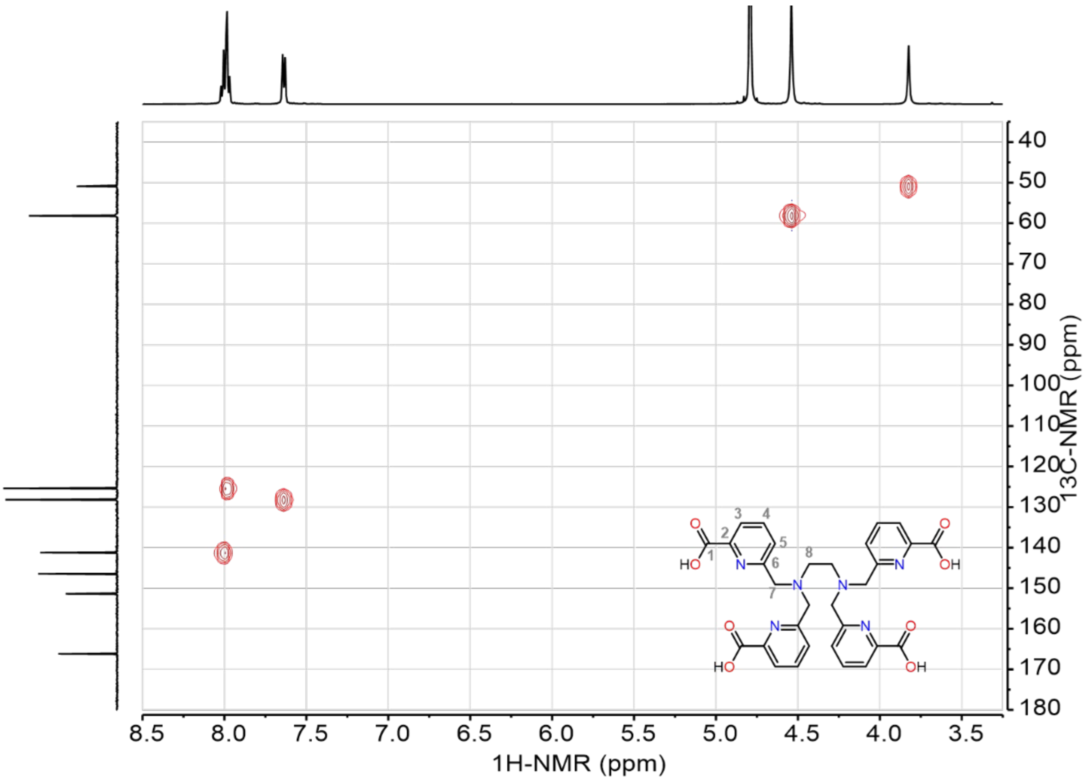

Figure S8: HSQC spectrum (500 MHz, D_2O, pD 2.8, 298K) of compound H_4TPAEN.

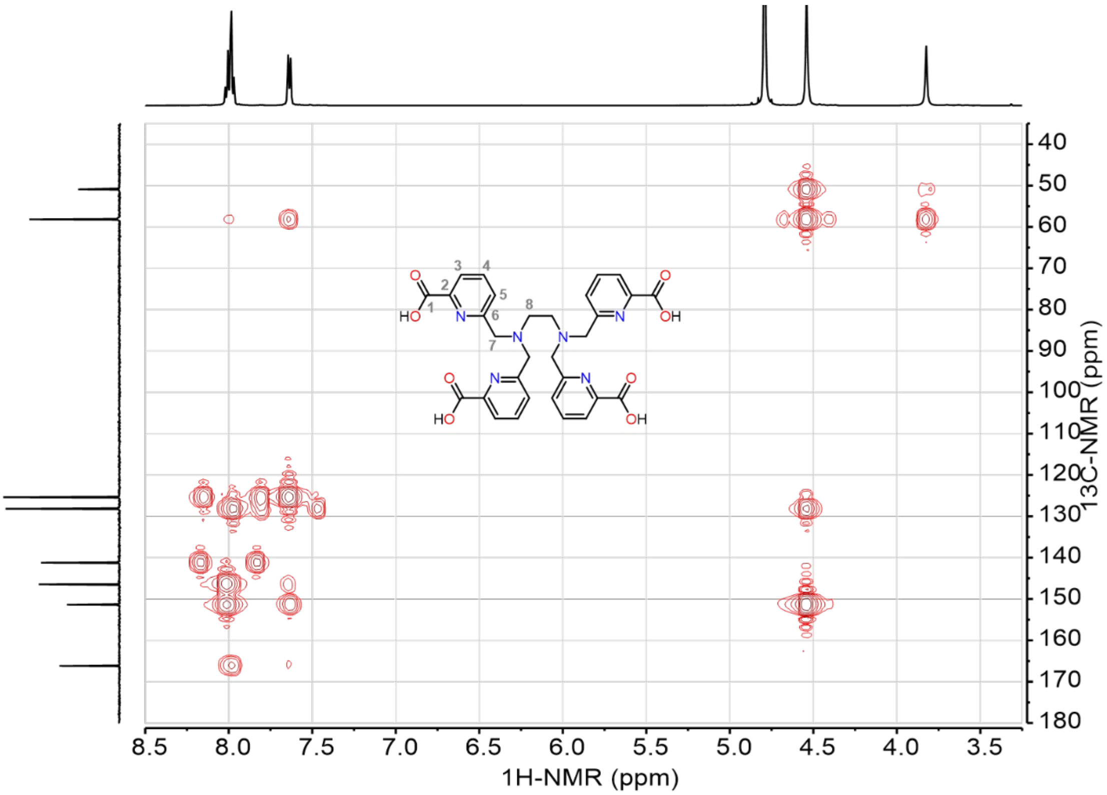

Figure S9: HMBC spectrum (500 MHz, D_2O, pD 2.8, 298K) of compound H_4TPAEN.

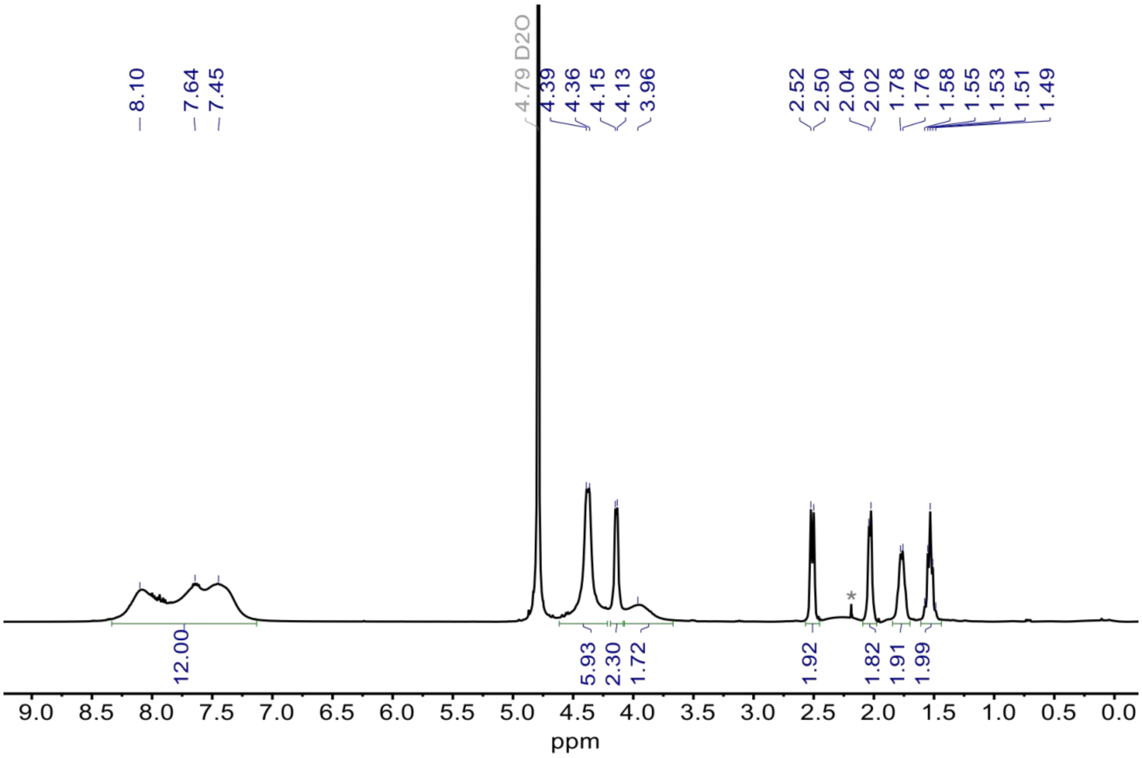

Figure S10: ^{1}H NMR spectrum (500 MHz, D_2O, pD 2.2, 298K) of compound $H_4TPADAC$. The asterisk denotes a signal due to residual acetone.

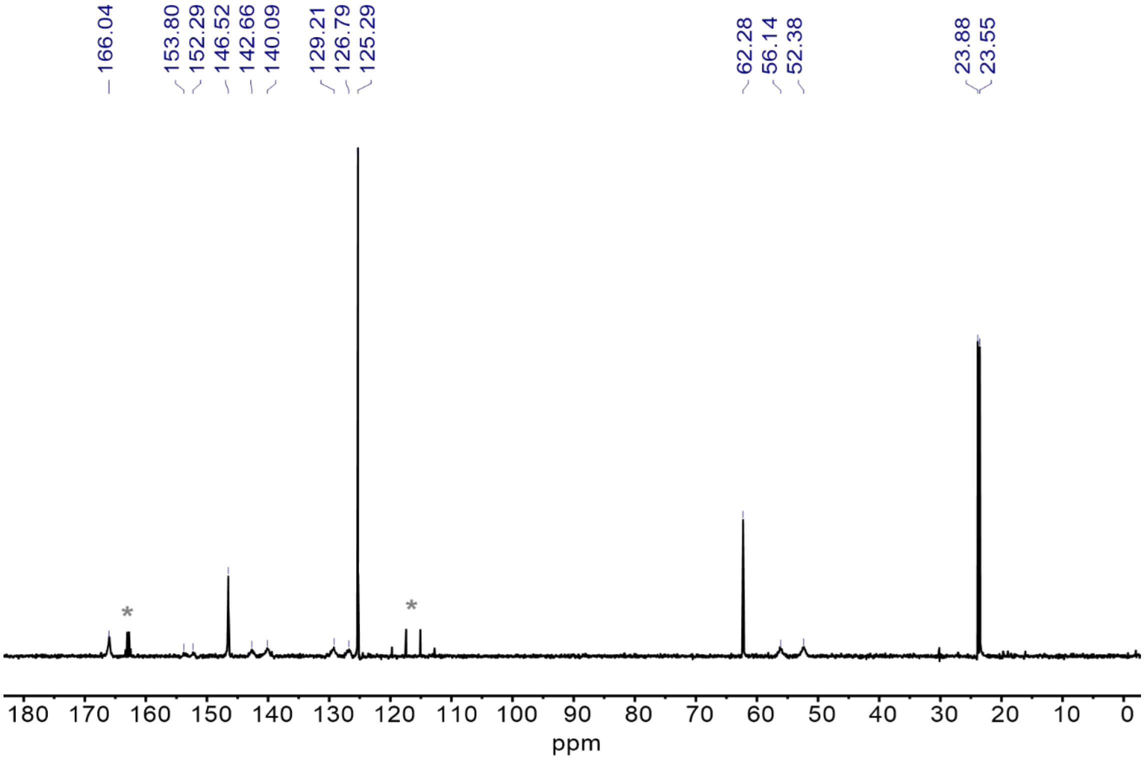

Figure S11: ^{13}C NMR spectrum (126 MHz, D_2O, pD 2.2, 298K) of compound $H_4TPADAC$. The asterisk denotes signals due to residual trifluoroacetic acid.

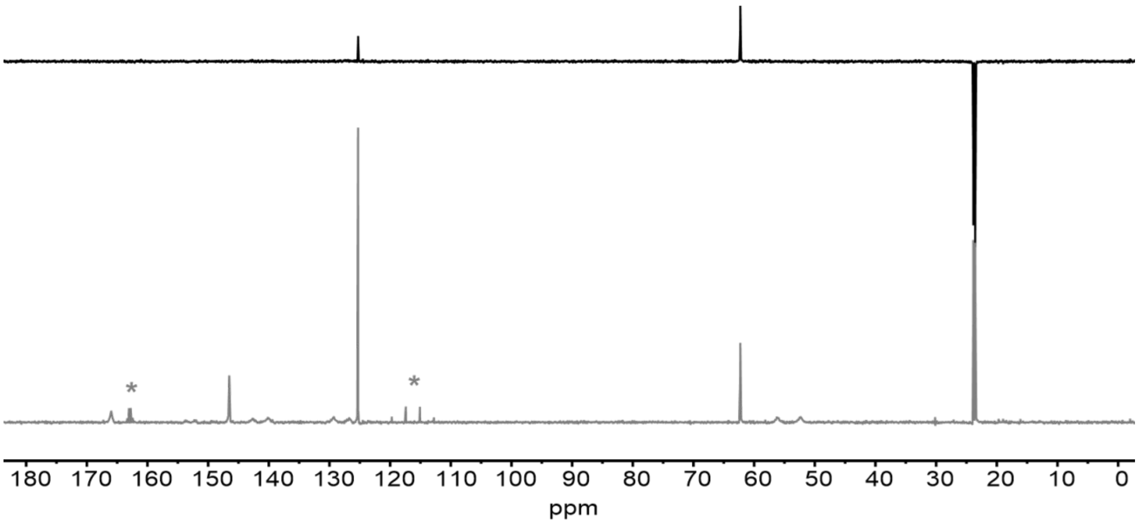

Figure S12: DEPT-135 spectrum (126 MHz, D_2O, pD 2.2, 298K) of compound H_4TPADAC. The asterisk denotes signals due to residual trifluoroacetic acid.

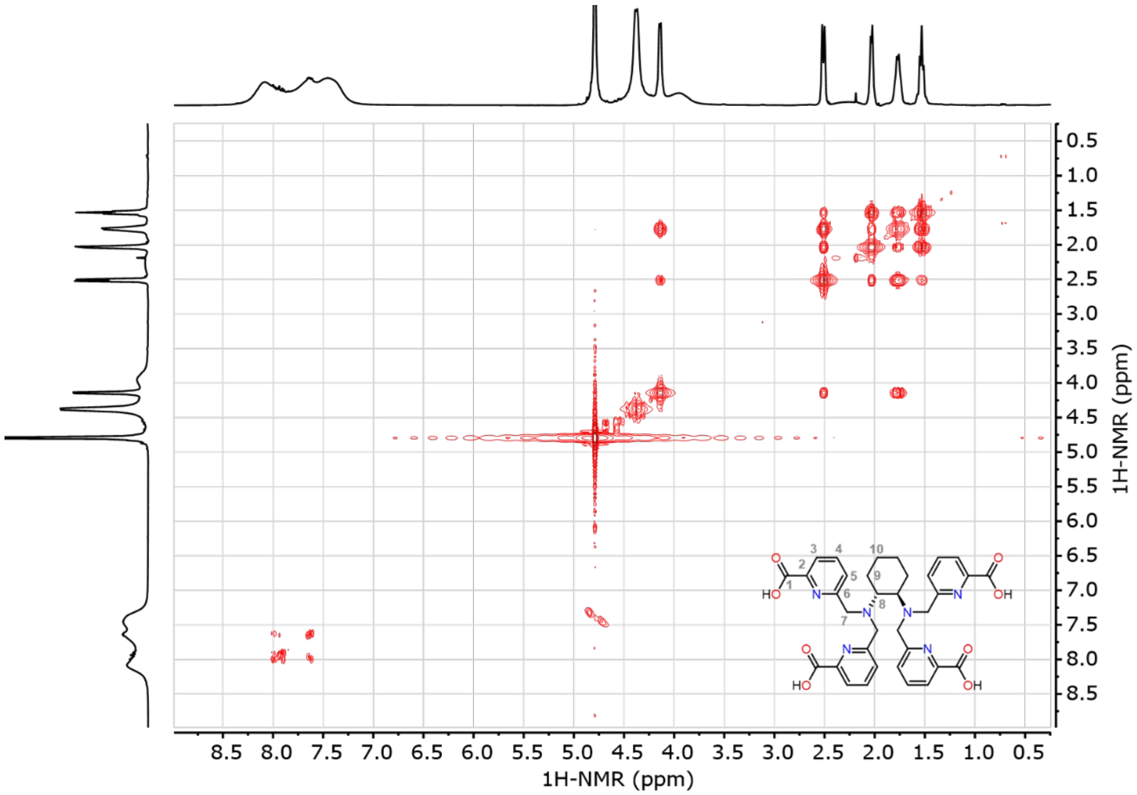

Figure S13: COSY spectrum (500 MHz, D_2O, pD 2.2, 298K) of compound H_4TPADAC.

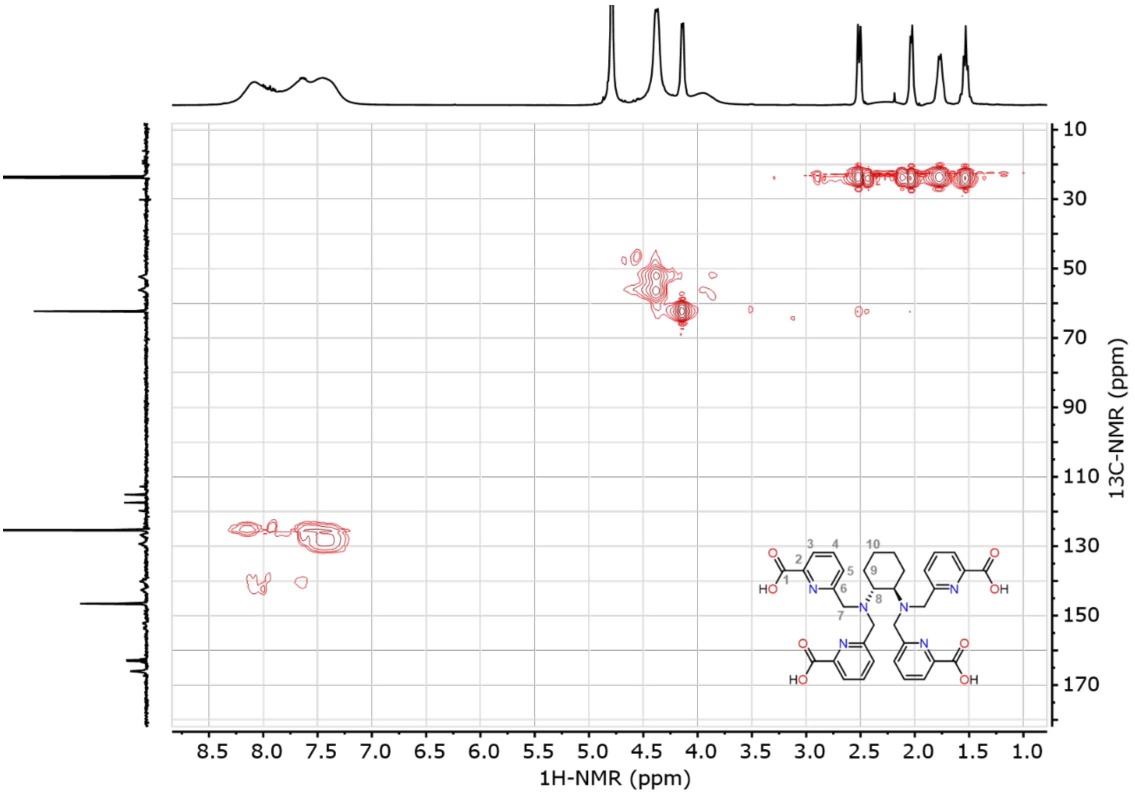

Figure S14: HSQC spectrum (500 MHz, D_2O, pD 2.2, 298K) of compound H_4TPADAC.

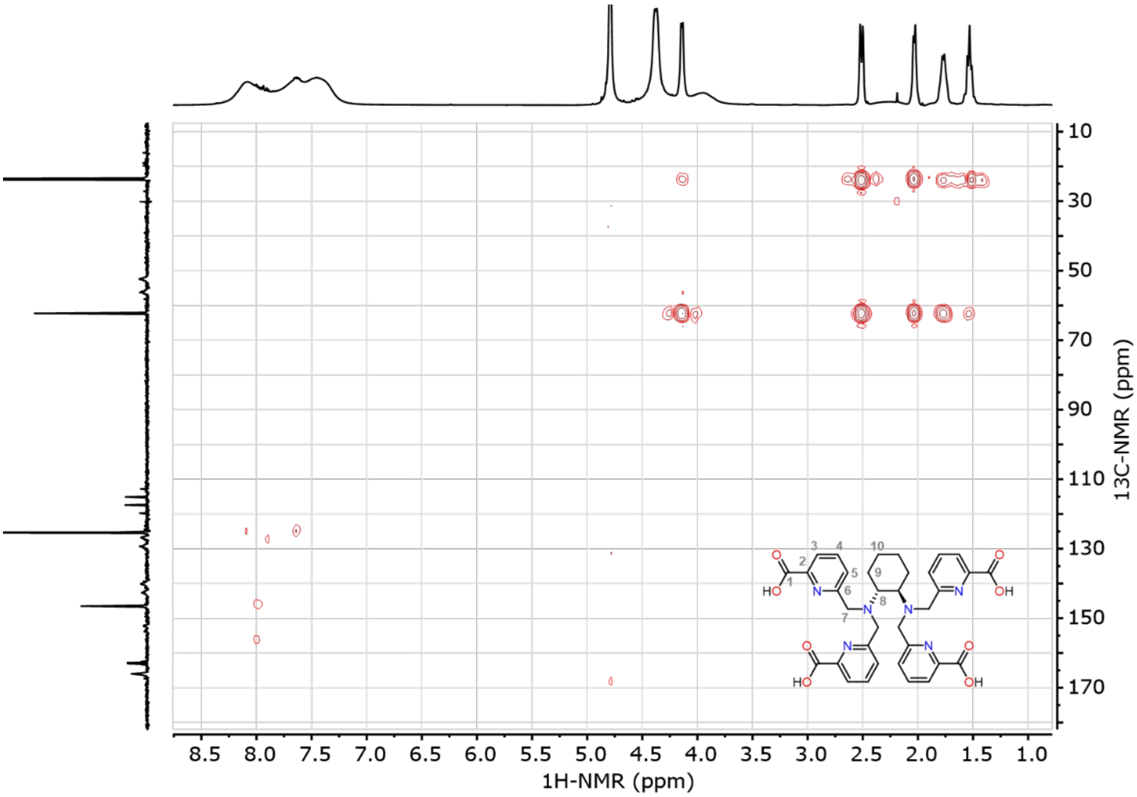

Figure S15: HMBC spectrum (500 MHz, D_2O, pD 2.2, 298K) of compound H_4TPADAC.

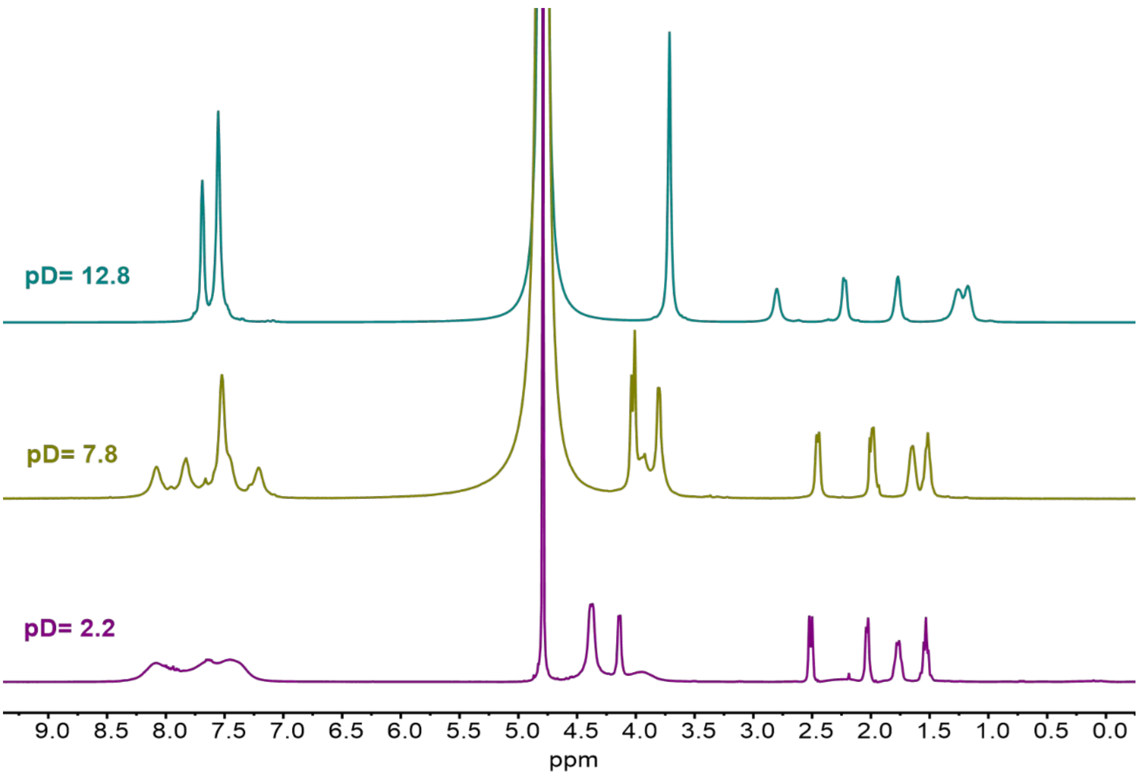

Figure S16: ^{1}H NMR spectrum (500 MHz, D_2O, 298K) of compound H_4TPADAC at different pD values (indicated in the figure).

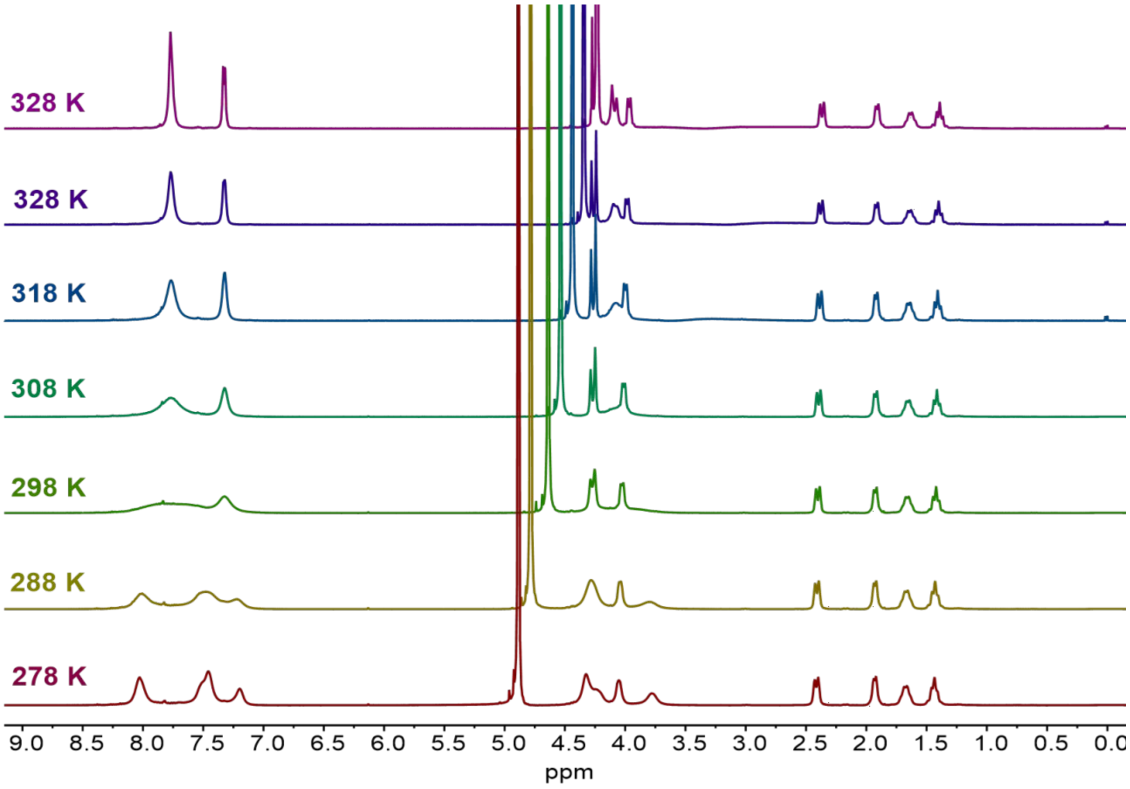

Figure S17: Variable temperature ^{1}H NMR spectrum (400 MHz, D_2O, pD 2.2) of compound H_4TPADAC.

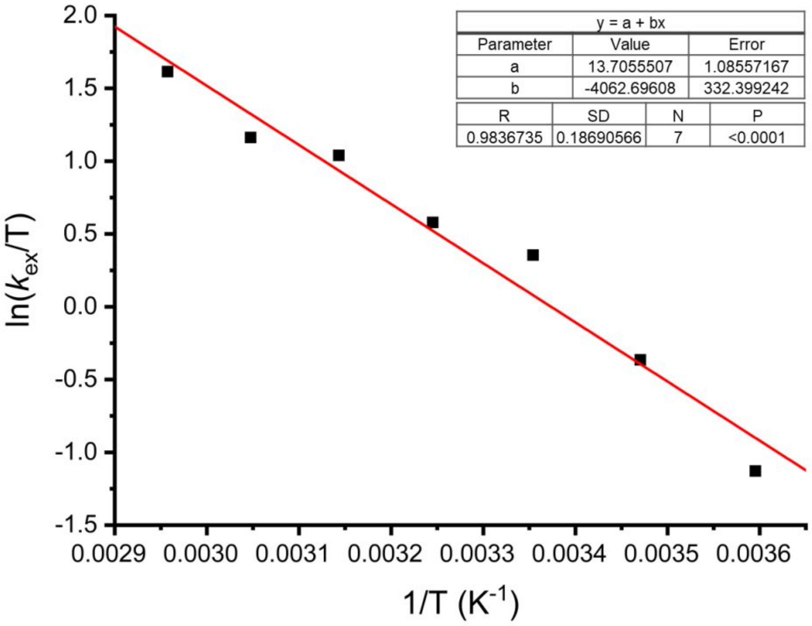

Figure S18: Eyring plot of ln(k_{ex}/T) vs 1/T for the interconversion process studied by NMR for H_4TPADAC. Inset: values obtained from the linear fit used to determine the activation parameters of the interconversion process, following Eyring's equation.

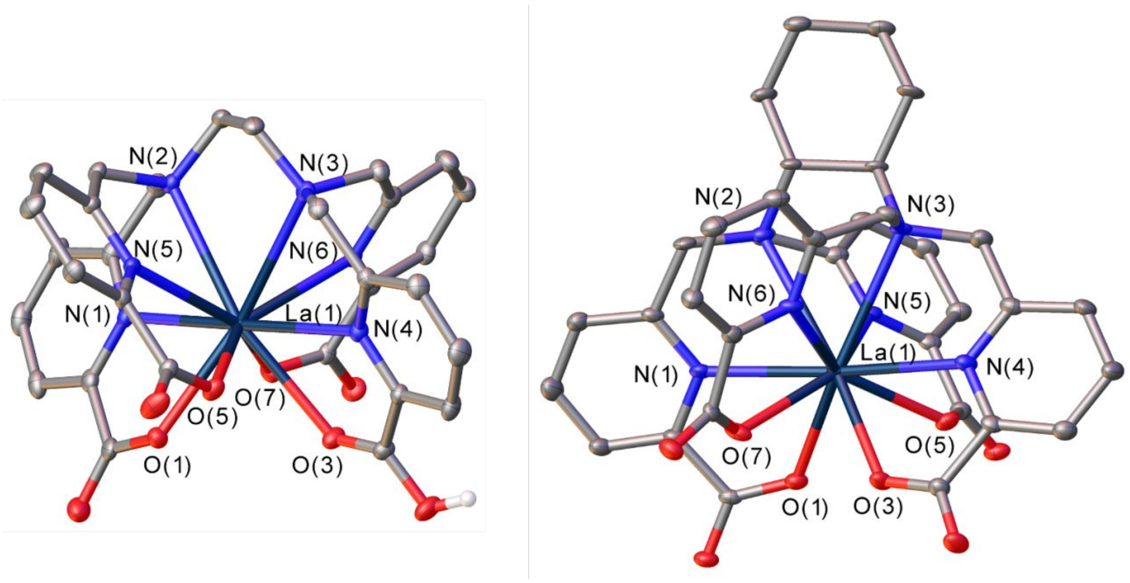

Figure S19: Views of the X-ray crystal structures of the [La(HTPAEN)] (left) and [La(TPADAC)]- (right) complexes with ellipsoids plotted at the 50% probability level.

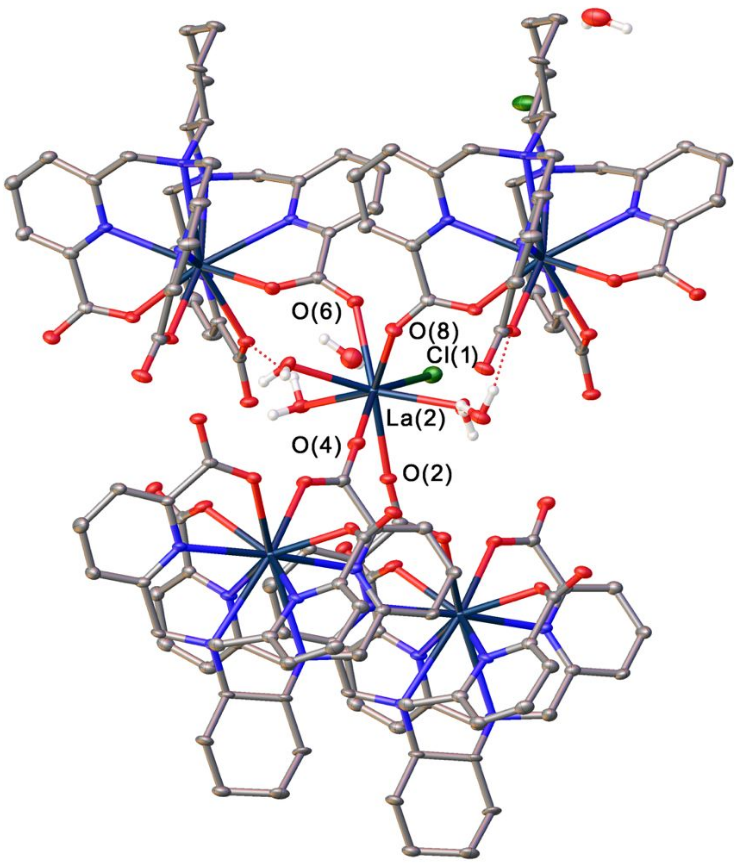

Figure S20: View of the X-ray crystal structure of $[LaCl(H_2O)_3][La(TPADAC)]Cl\cdot 3H_2O$ depicting the excess La^{3+} coordinating four oxygen atoms of carboxylate groups of four different $[La(TPADAC)]^-$ entities (ellipsoids plotted at the 50% probability level).

Table S3: Experimental ^1H NMR chemical shifts (ppm) of $[La(TPAEN)]^-$ and $[La(TPADAC)]^-$ (500 MHz, D_2O, 298K).

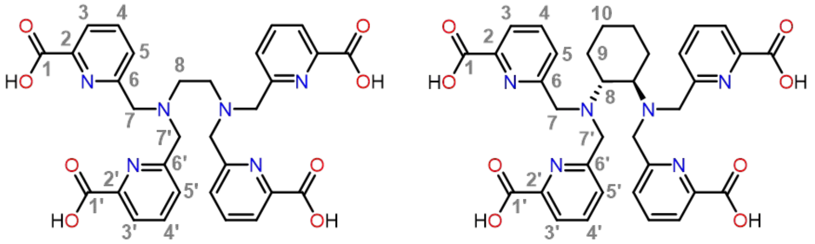

	**$[La(TPAEN)]^-$ (pD 7.4)**	**$[La(TPADAC)]^-$ (pD 7.8)**
**H4'**	7.98 (t, 2H), J = 7.78 Hz	7.99 (m, 6H)
**H4**	7.90 (m, 4H)	
**H3'**		
**H3**	7.76 (d, 2H), J = 7.6 Hz	7.77 (s, 2H)
**H5'**	7.47 (d, 2H), J = 7.8 Hz	7.46 (d, 4H), J = 17.5 Hz
**H5**	7.41 (d, 2H), J = 7.7 Hz	
**H7 eq**	4.02 (d, 2H), J = 17.1 Hz	4.15 (d, 2H), J = 15.0 Hz
**H7' eq**	3.91 (d, 2H), J = 15.5 Hz	4.07 (d, 2H), J = 13.9Hz
**H7 ax**	3.81 (d, 2H), J = 17.3 Hz	3.13 (d, 2H), J = 15.1 Hz

**H7' ax**	3.25 (d, 2H), J = 15.7 Hz	3.59 (d, 2H), J = 13.9 Hz
**H8 eq**	2.84 (d, 2H), J = 10.4 Hz	2.44 (m, 2H)
**H8 ax**	2.67 (d, 2H), J = 10.3 Hz	
**H9 eq**	-	2.10 (m, 2H)
**H10 eq**	-	1.83 (s, 2H)
**H9 ax**	-	1.64 (s, 2H)
**H10 ax**	-	1.11 (m, 2H)

Table S4: Experimental ^{13}C NMR chemical shifts (ppm) of $[La(TPAEN)]^-$ and $[La(TPADAC)]^-$ (500 MHz, D_2O, 298K).

	**Type**	**$[La(TPAEN)]^-$ (pD 7.4)**	**$[La(TPADAC)]^-$ (pD 7.8)**
**C1/C1'**	C	173.49	173.28
**C1/C1'**	C	171.51	171.21
**C6**	C	160.35	155.22
**C6'**	C	155.31	159.39
**C2**	C	151.74	151.82, 150.68
**C2'**	C	150.94	
**C4**	CH	141.15	141.69, 140.58
**C4'**	CH	140.39	
**C5'**	CH	126.10	126.51, 124.99
**C5**	CH	124.65	
**C3'**	CH	123.70	123.75, 123.42
**C3**	CH	123.57	
**C8**	CH_2/CH	59.36	66.05
**C7**	CH_2	63.37	59.91
**C7'**	CH_2	62.43	53.68
**C9**	CH_2	-	24.30
**C10**	CH_2	-	24.00

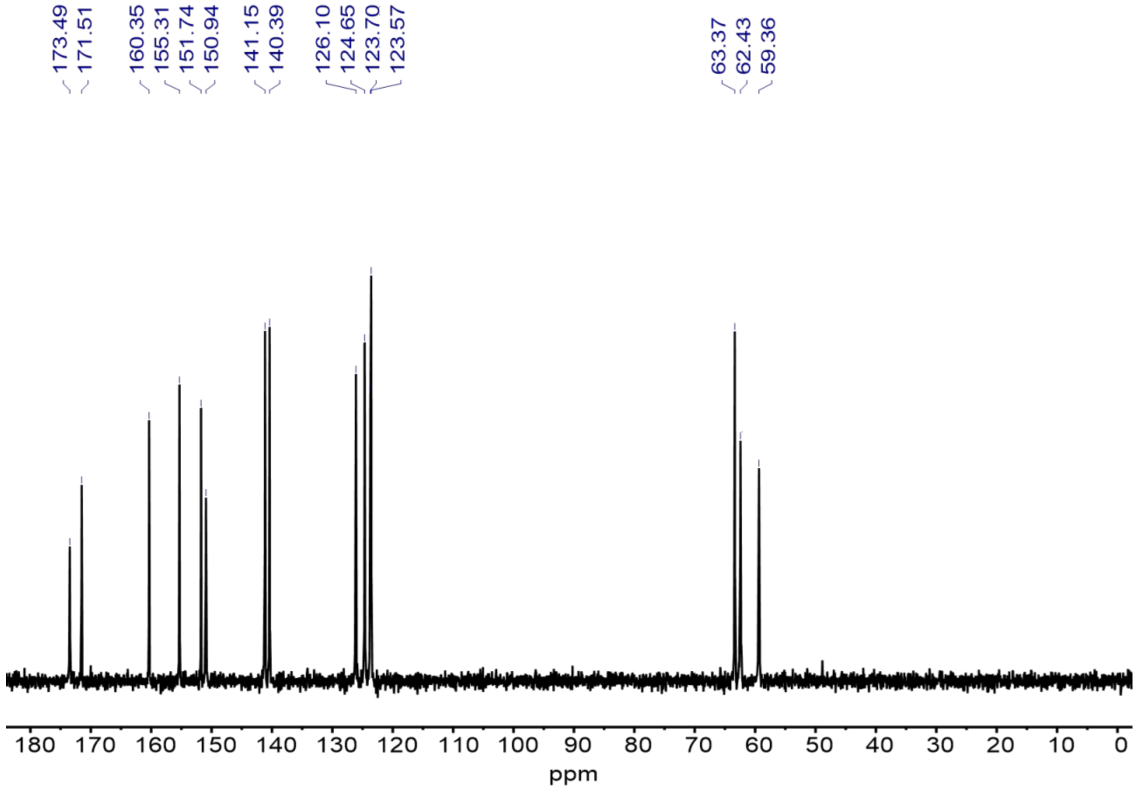

Figure S21: ^{13}C NMR spectrum (126 MHz, D_2O, pD 7.4, 298K) of compound $[La(TPAEN)]^-$.

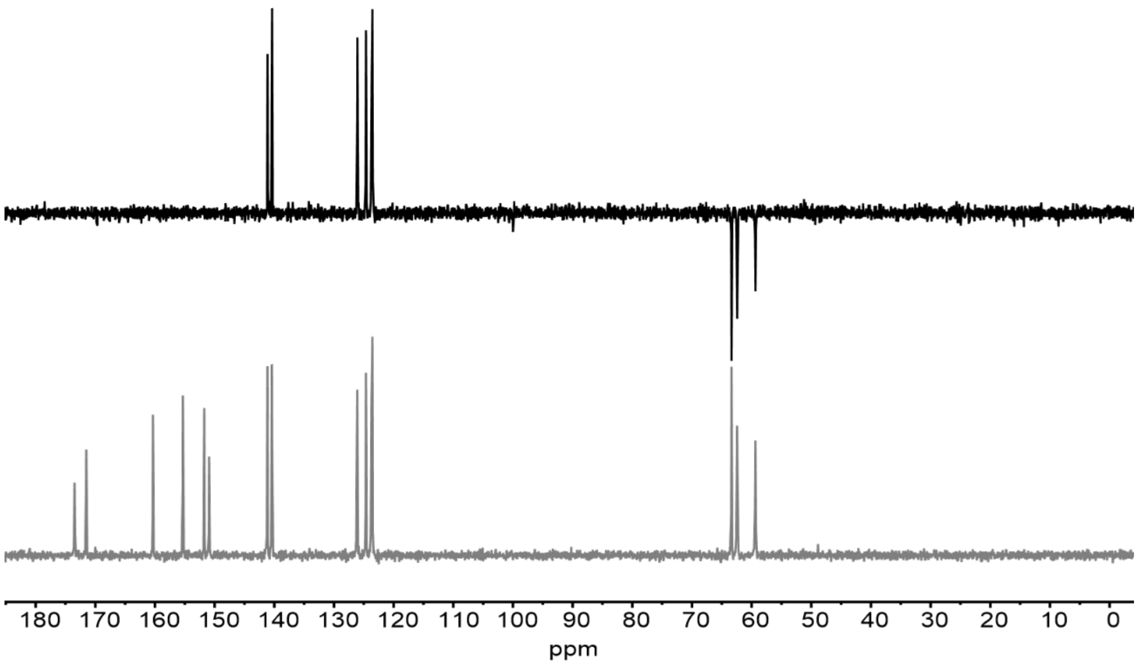

Figure S22: DEPT-135 spectrum (126 MHz, D_2O, pD 7.4, 298K) of compound $[La(TPAEN)]^-$.

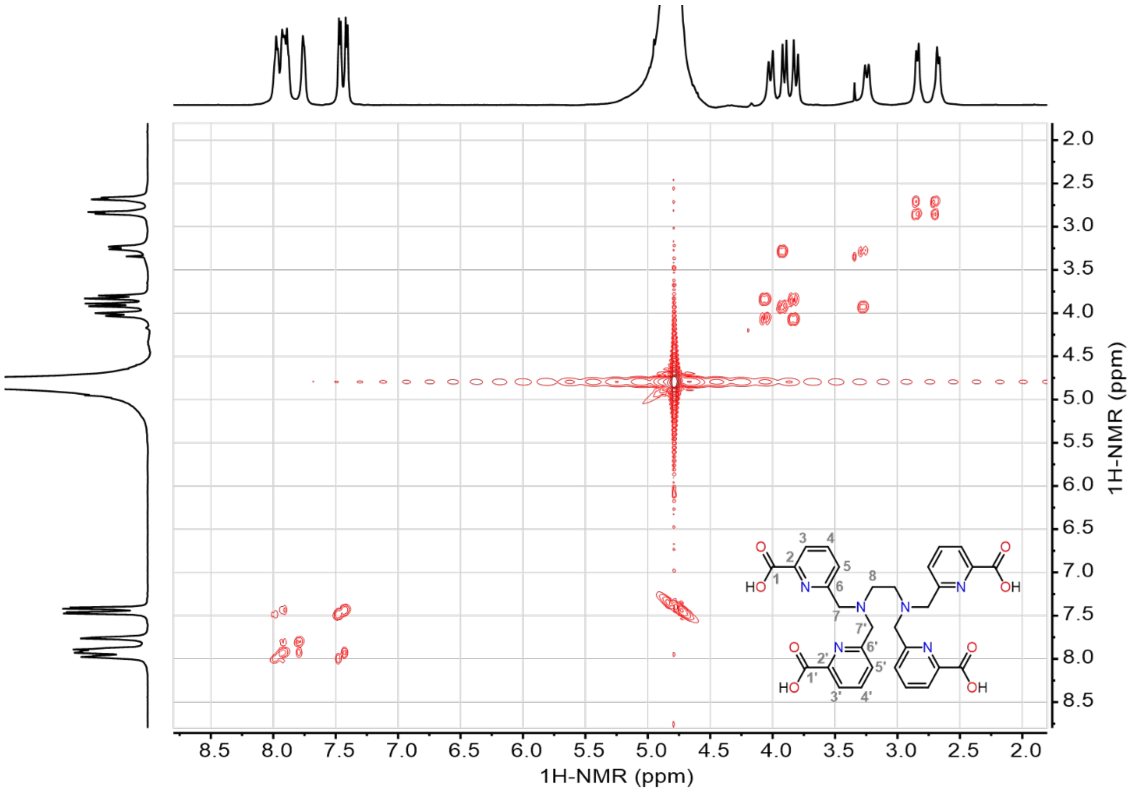

Figure S23: COSY spectrum (500 MHz, D_2O, pD 7.4, 298K) of compound $[La(TPAEN)]^-$.

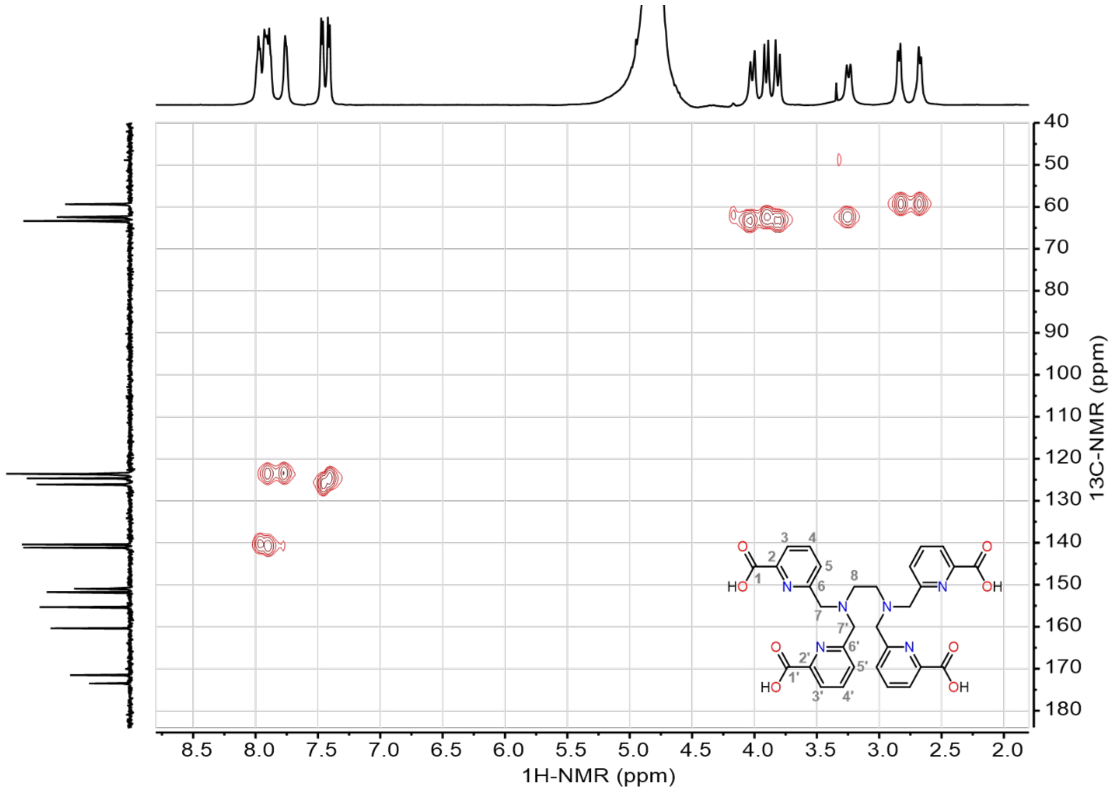

Figure S24: HSQC spectrum (500 MHz, D_2O, pD 7.4, 298K) of compound $[La(TPAEN)]^-$.

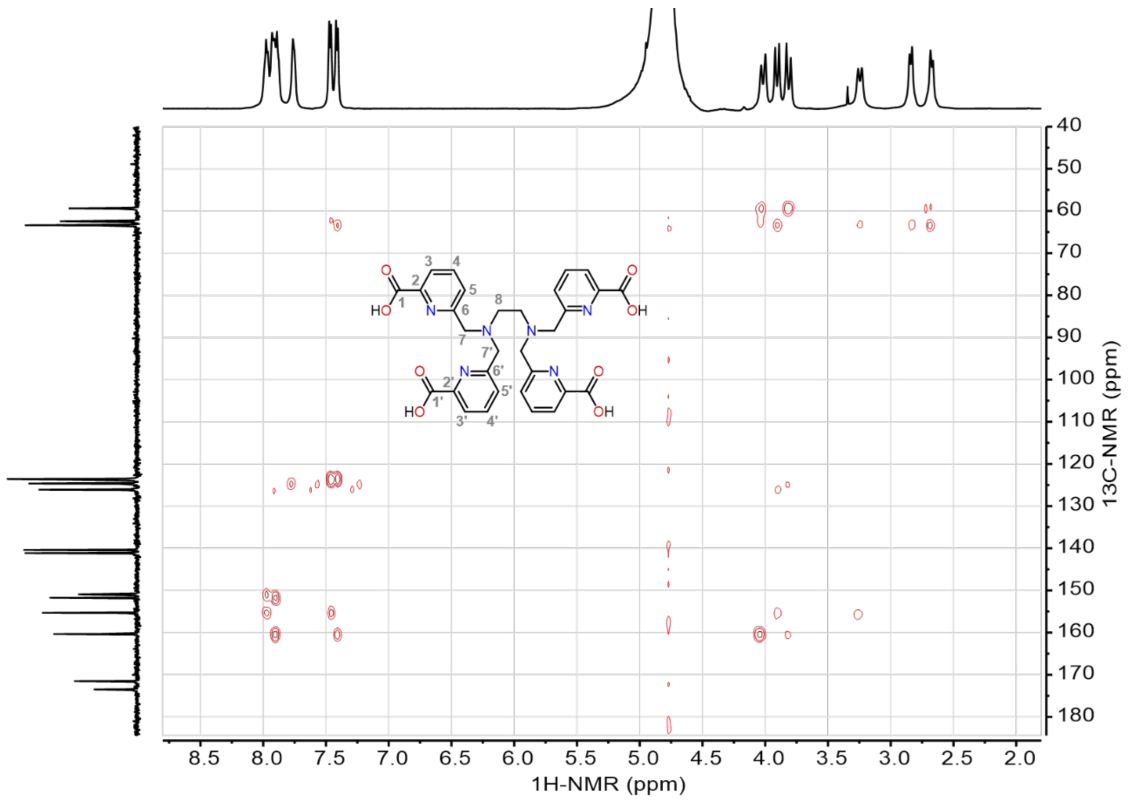

Figure S25: HMBC spectrum (500 MHz, D_2O, pD 7.4, 298K) of compound $[La(TPAEN)]^-$.

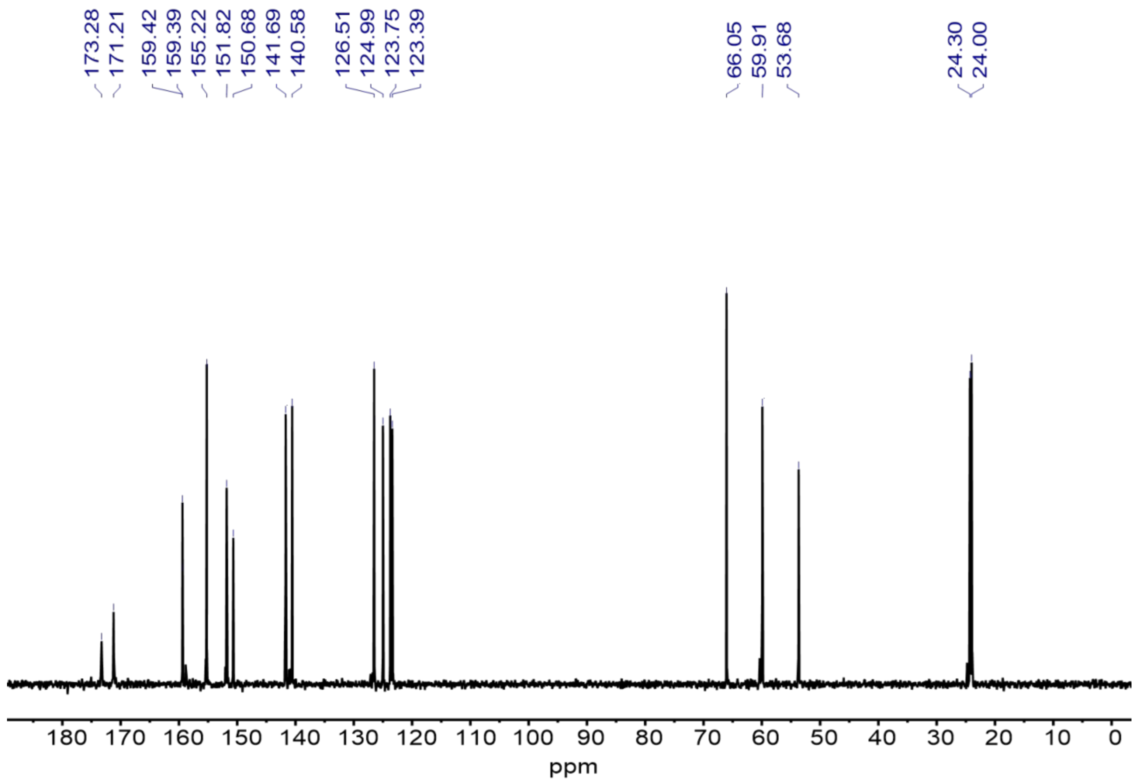

Figure S26: ^{13}C NMR spectrum (126 MHz, D_2O, 298K) of compound $[La(TPADAC)]^-$.

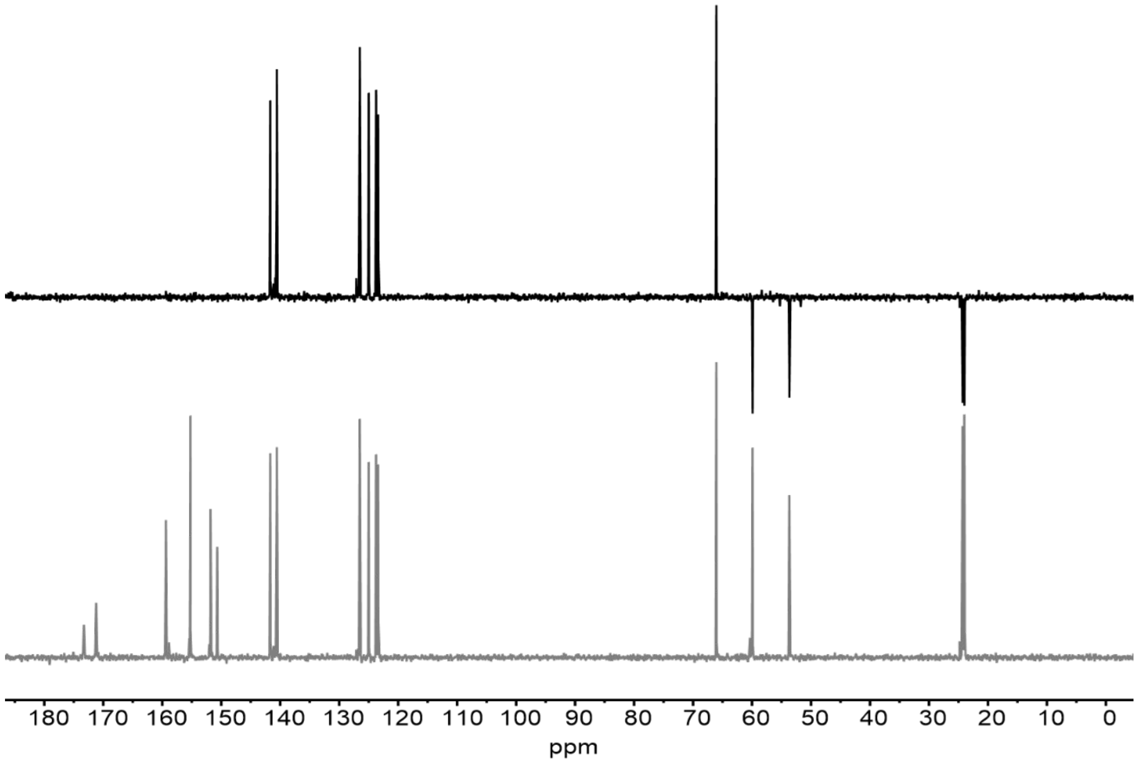

Figure S27: DEPT-135 spectrum (126 MHz, D_2O, 298K) of compound [La(TPADAC)]$^-$.

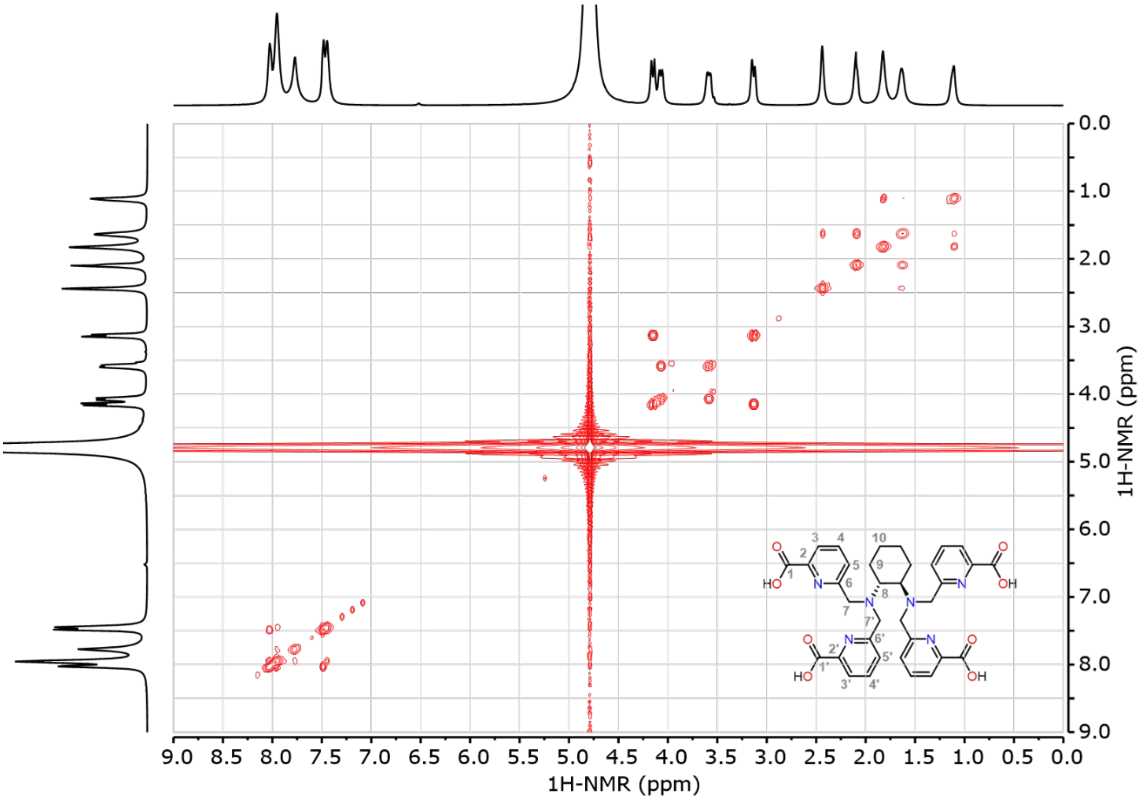

Figure S28: COSY spectrum (500 MHz, D_2O, pD 7.8, 298K) of compound [La(TPADAC)]$^-$.

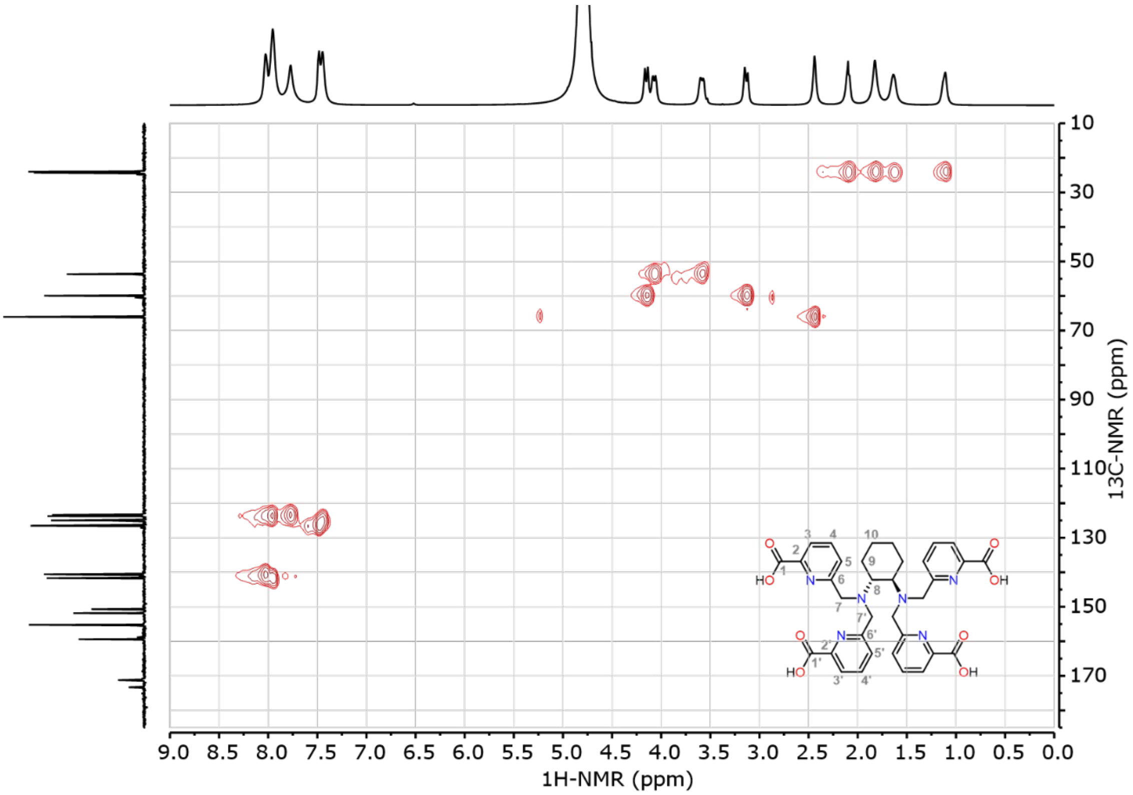

Figure S29: HSQC spectrum (500 MHz, D_2O, pD 7.8, 298K) of compound $[La(TPADAC)]^-$.

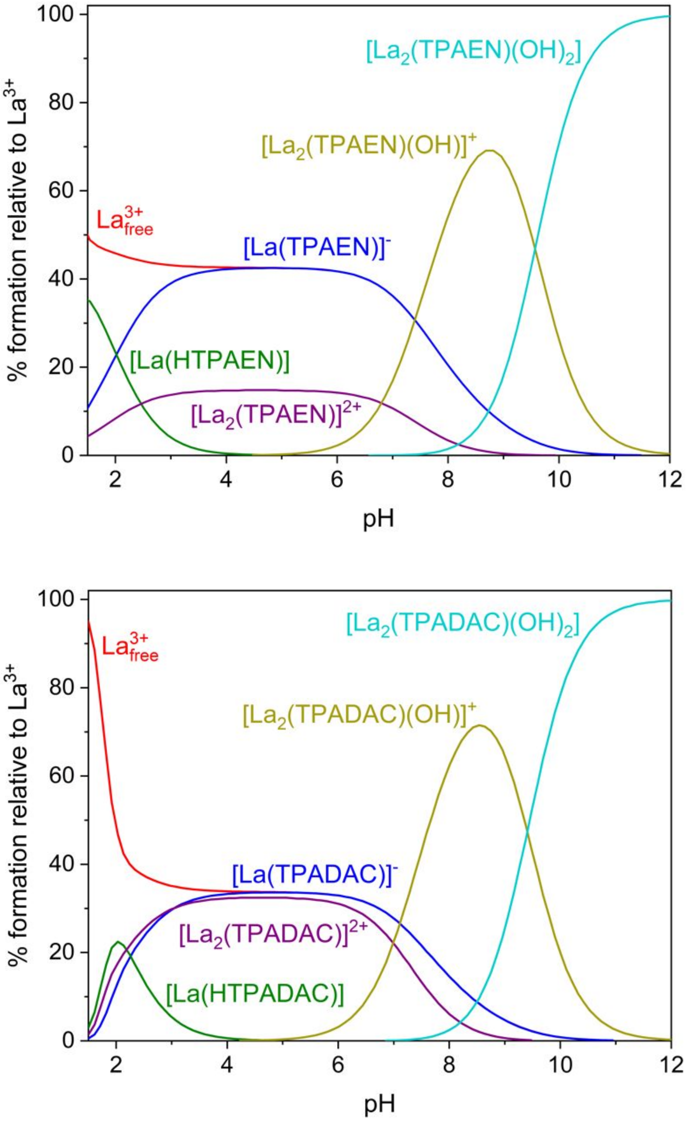

Figure S30: Species distribution curves calculated for the 2:1 La^{3+}:$TPAEN^{4-}$ (top) and La^{3+}:$TPADAC^{4-}$ (bottom) systems ($c_{Lig}=10^{-3}$ M, $c_{La3+}=2\cdot10^{-3}$ M; I = 0.15 M NaCl, 25 ºC).

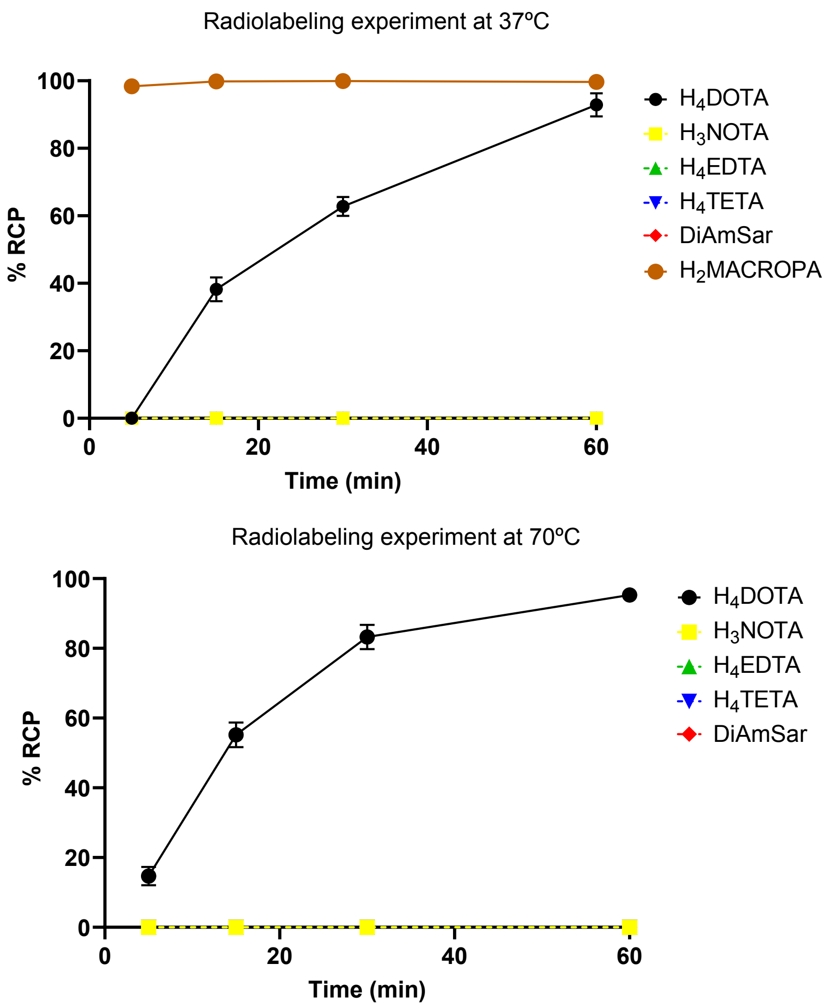

Figure S31: Radiolabeling tests results of H_4DOTA, H_3NOTA, H_4TETA, H_4EDTA, DiAmSar and $H_2MACROPA$ at 37ºC (top) and/or 70ºC (bottom) and pH 4.5 with ^{135}La.

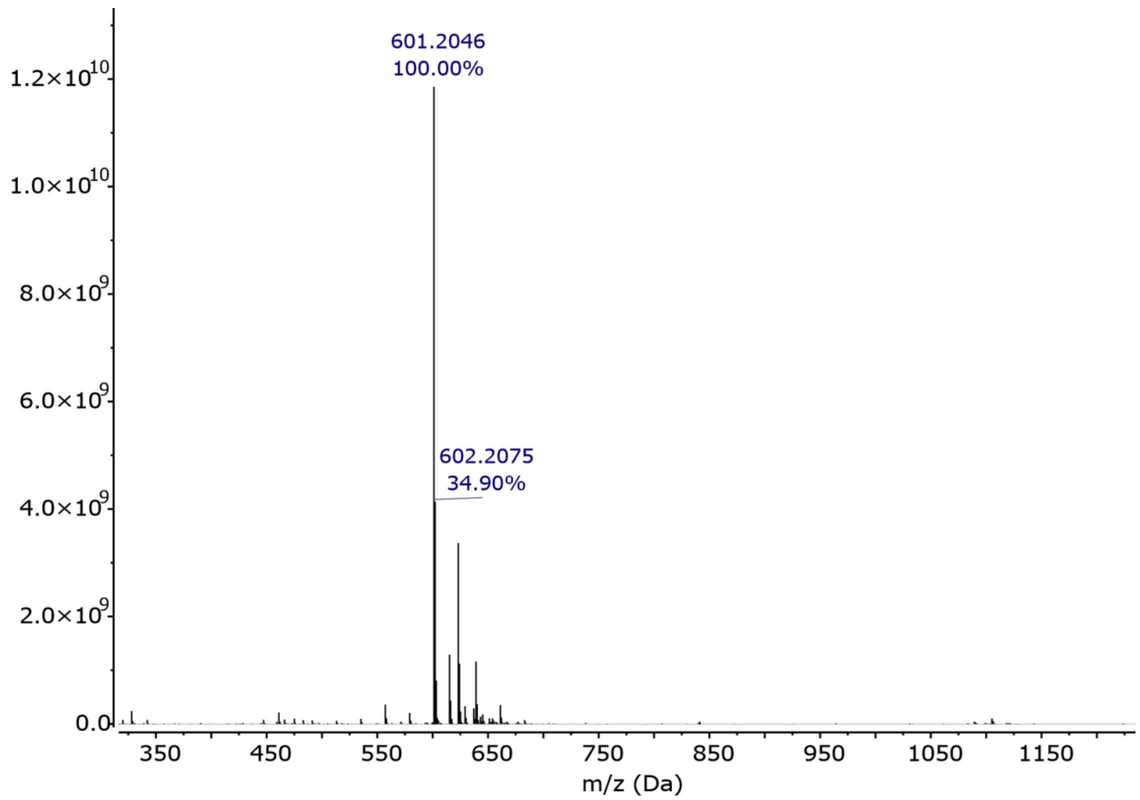

Figure S32: Experimental high resolution mass spectrum (ESI$^+$) of compound H_4TPAEN.

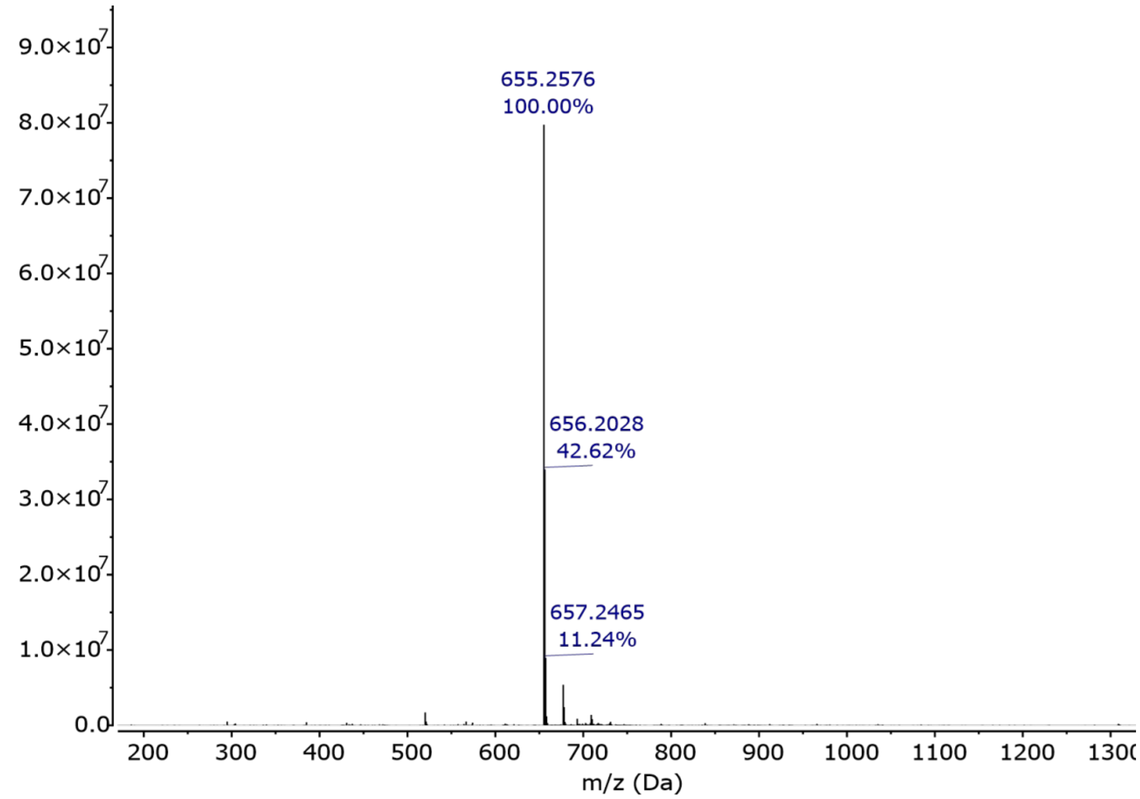

Figure S33: Experimental high resolution mass spectrum (ESI$^+$) of compound H_4TPADAC.

Table S5: MPLC separation method (with mobile phases A= 0.1% TFA aqueous solution and B= CH_3CN + 20% A).

Column Volumes (CV)	Time (min)	B (%)	Flow (mL/min)
0.00	0,00	0	15
5.00	6.75	0	15
16.00	21.75	15	15
24.90	33.75	15	15
36.00	48.75	100	15
42.00	56.85	100	15

Table S6: HPLC separation method for the purification of both chelators (with mobile phases A= ammonium acetate 10 mM aqueous solution and B= CH_3CN + 10% A).

Time (min)	B (%)	Flow (mL/min)	Retention time (min)	
0	5	20	**H_4TPAEN**	**H_4TPADAC**
15	5	20	3.74	8.53
25	95	20		
35	95	20		

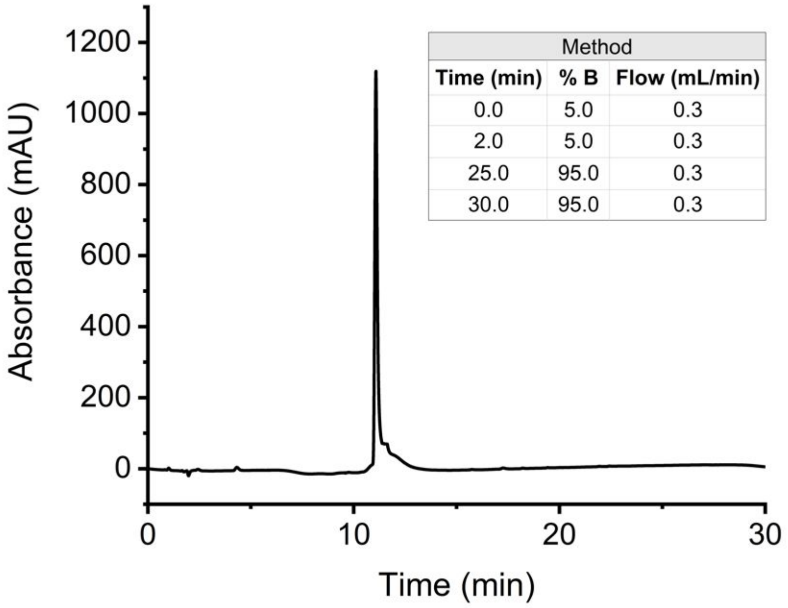

Figure S34: HPLC analysis (272 nm) of H_4TPAEN, retention time 11.08 min. Inset: separation method with A= H_2O + 0.04% TFA, B= CH_3CN + 0.04% TFA.

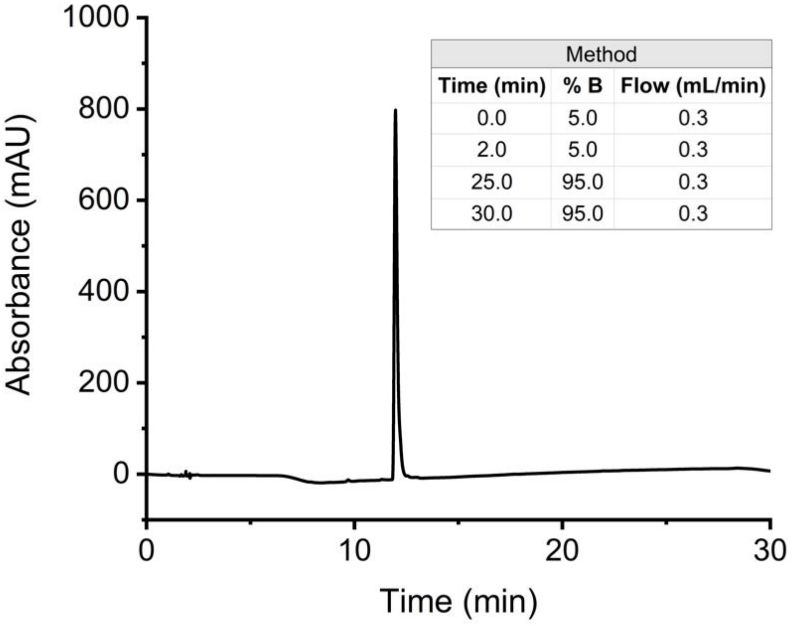

Figure S35: HPLC analysis (272 nm) of H_4TPADAC, retention time 11.93 min. Inset: separation method with A= H_2O + 0.04% TFA, B= CH_3CN + 0.04% TFA.

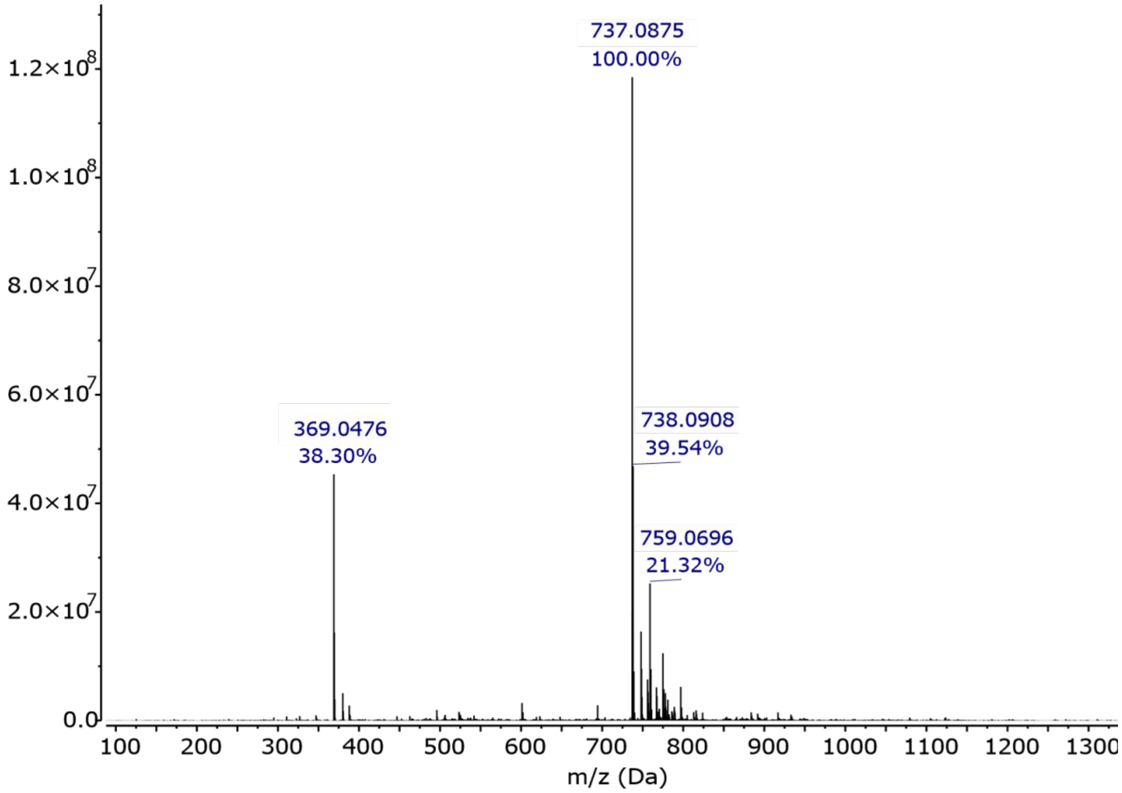

Figure S36: Experimental high resolution mass spectrum (ESI$^+$) of compound [La(TPAEN)]$^-$.

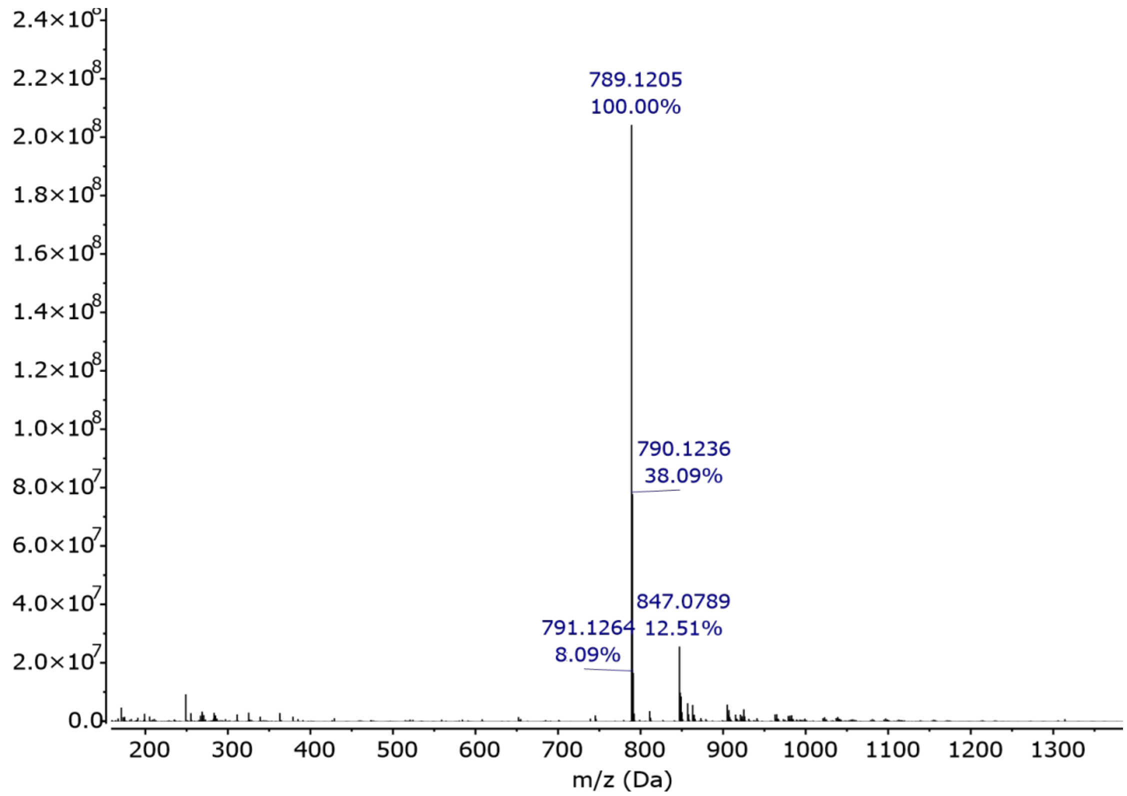

Figure S37: Experimental high resolution mass spectrum (ESI$^-$) of compound [La(TPADAC)]$^-$.

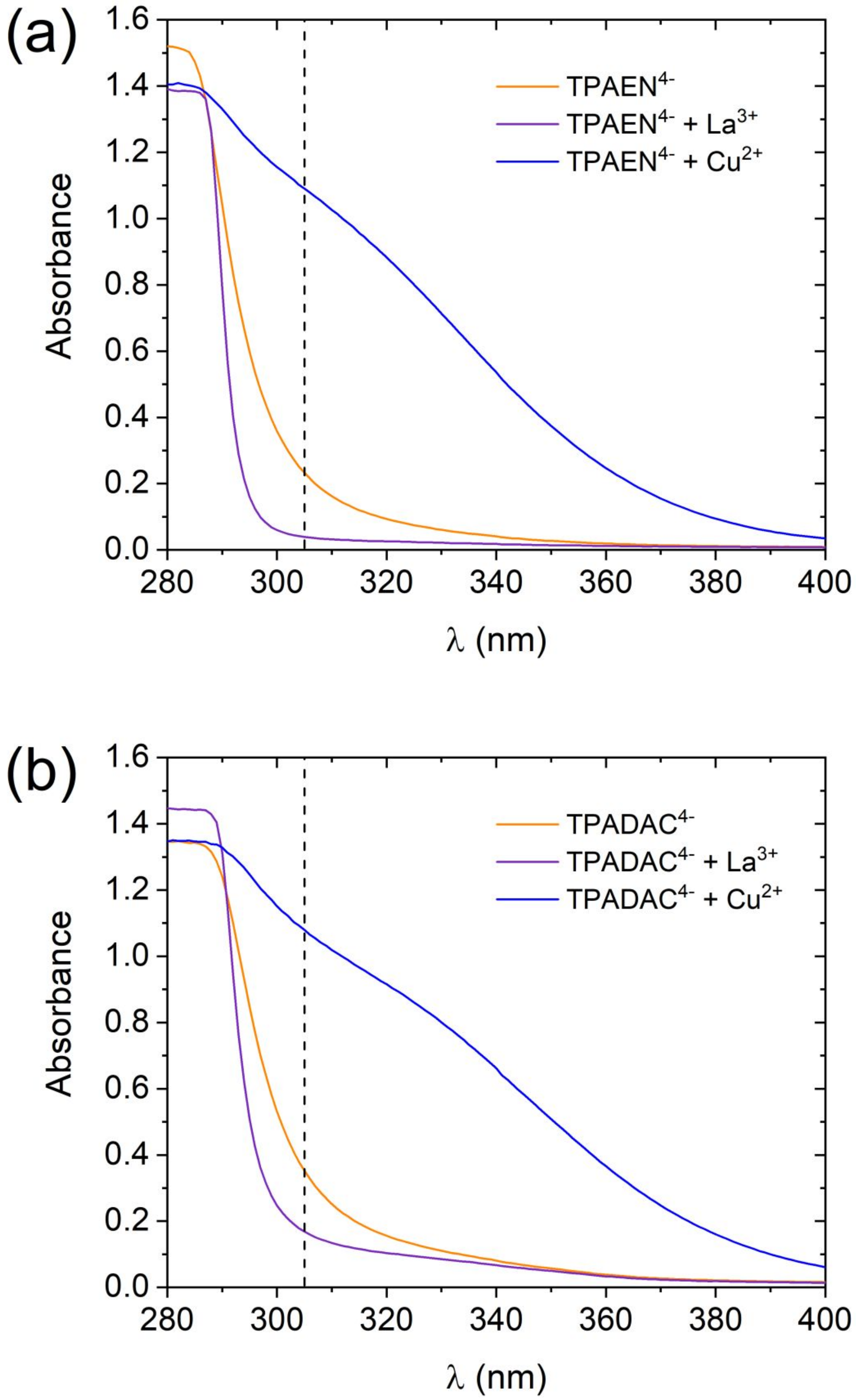

Figure S38: Absorption spectra of the free ligands and their La^{3+} and Cu^{2+} complexes (M:L = 1:1) recorded in H_2O solutions: (a) [$TPAEN^{4-}$] = 0.487 mM, pH = 4.57; (b) [$TPADAC^{4-}$] = 0.517 mM, pH = 4.57. The vertical dashed lines indicate the wavelength used for kinetic experiments (305 nm).

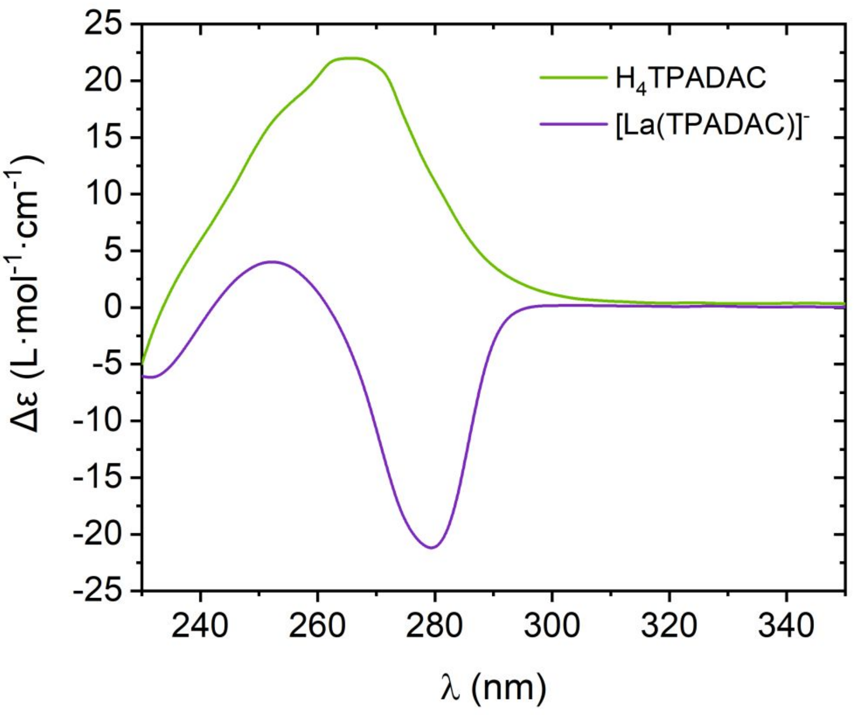

Figure S39: CD spectra recorded from aqueous solutions of the H_4TPADAC ligand (0.170 mM, pH 6.35) and the $[La(TPADAC)]^-$ complex (0.28 mM, pH 4.72).

Table S7: Crystal data and structure refinement $[H_5TPAEN]Cl\cdot 3H_2O$, $[La(HTPAEN)]\cdot 12H_2O$ and $[LaCl(H_2O)_3][La(TPADAC)]Cl\cdot 3H_2O$.

	**$[H_5TPAEN]Cl\cdot 3H_2O$**	**$[La(HTPAEN)]\cdot 12H_2O$**	**$[LaCl(H_2O)_3]$ $[La(TPADAC)]Cl\cdot 3H_2O$**
Empirical formula	$C_{30}H_{35}ClN_6O_{11}$	$C_{30}H_{49}LaN_6O_{20}$	$C_{34}H_{42}Cl_2La_2N_6O_{14}$
Molecular weight MW	691.09	952.66	1107.45
Crystal system	Monoclinic	Triclinic	Monoclinic
Space group	$P2_1/n$	P-1	$P2_1$
a/Å	8.3094(3)	12.3868(8)	9.3631(5)
b/Å	18.1723(6)	12.7323(8)	99.526(2)
c/Å	20.4505(7)	12.8633(9)	19.9086(9)
α/°	90	86.741(2)	90
β/°	92.2180(10)	90.033(2)	10.7302(5)
γ/°	90	86.685(2)	90
Volume (Å^3)	3085.73(18)	2022.0(2)	1972.59(17)
Z	4	2	2
ρ_{calc} (g/cm^3)	1.488	1.565	1.865
μ (mm^{-1})	0.197	1.142	2.347
θ range	1.993° - 28.339°	2.232° - 28.328°	2.074° - 28.332°

R_{int}	0.0500	0.0466	0.0472
Measured reflections	101825	125469	63920
Independent reflections / unique (I >2σ (I)	7680 / 6903	10072 / 9929	9797/9639
Goodness-of-fit on F^2	1.046	1.222	1.092
R_1	0.0343	0.0414	0.0351
wR_2 (all data)	0.0910	0.1143	0.0916
Larg. diff. peak and hole (eÅ^{-3})	0.41 and -0.30	2.12 and -0.77	2.849 and -1.78
